# Cetuximab plus FOLFOXIRI versus cetuximab plus FOLFOX as conversion regimen in *RAS/BRAF* wild-type patients with initially unresectable colorectal liver metastases (TRICE trial): A randomized controlled trial

**DOI:** 10.1371/journal.pmed.1004389

**Published:** 2024-05-10

**Authors:** De-Shen Wang, Chao Ren, Shan-Shan Li, William Pat Fong, Xiao-Jun Wu, Jian Xiao, Bin-Kui Li, Yun Zheng, Pei-Rong Ding, Gong Chen, Miao-Zhen Qiu, Zhi-Qiang Wang, Feng-Hua Wang, Hui-Yan Luo, Feng Wang, Xiao-Zhong Wang, Ling-Yun Wang, De-Jin Xie, Tao Chen, Li-Ren Li, Zhen-Hai Lu, Xiao-Hui Zhai, Tian-Shu Liu, Ying Yuan, Jia-Qi Chen, Qiong Tan, Zhi-Zhong Pan, De-Sen Wan, Rong Zhang, Yun-Fei Yuan, Rui-Hua Xu, Yu-Hong Li

**Affiliations:** 1 Department of Medical Oncology, State Key Laboratory of Oncology in South China, Guangdong Provincial Clinical Research Center for Cancer, Sun Yat-sen University Cancer Center, Guangzhou, China; 2 Department of Internal Medicine, The 6th Affiliated Hospital of Sun Yat-Sen University, Guangzhou, China; 3 Department of Colorectal Surgery, State Key Laboratory of Oncology in South China, Guangdong Provincial Clinical Research Center for Cancer, Sun Yat-sen University Cancer Center, Guangzhou, China; 4 Department of Medical Oncology, Guangdong Provincial People’s Hospital, Southern Medical University, Guangzhou, China; 5 Department of Liver Surgery, State Key Laboratory of Oncology in South China, Guangdong Provincial Clinical Research Center for Cancer, Sun Yat-sen University Cancer Center, Guangzhou, China; 6 Shantou Central Hospital, Shantou, China; 7 Department of Internal Medicine, Sun Yat-Sen Memorial Hospital, Sun Yat-Sen University, Guangzhou, China; 8 Department of Medical Oncology, Zhongshan Hospital, Fudan University, Shanghai, China; 9 Department of Medical Oncology, The Second Affiliated Hospital, Zhejiang University School of Medicine, Hangzhou, China; 10 Department of Medical Imaging, State Key Laboratory of Oncology in South China, Guangdong Provincial Clinical Research Center for Cancer, Sun Yat-sen University Cancer Center, Guangzhou, China; 11 Research Unit of Precision Diagnosis and Treatment for Gastrointestinal Cancer, Chinese Academy of Medical Sciences, Guangzhou, China; University of Texas Southwestern Medical Center, UNITED STATES

## Abstract

**Background:**

It remains unclear whether intensification of the chemotherapy backbone in tandem with an anti-EGFR can confer superior clinical outcomes in a cohort of *RAS/BRAF* wild-type colorectal cancer (CRC) patients with initially unresectable colorectal liver metastases (CRLM). To that end, we sought to comparatively evaluate the efficacy and safety of cetuximab plus FOLFOXIRI (triplet arm) versus cetuximab plus FOLFOX (doublet arm) as a conversion regimen (i.e., unresectable to resectable) in CRC patients with unresectable CRLM.

**Methods and findings:**

This open-label, randomized clinical trial was conducted from April 2018 to December 2022 in 7 medical centers across China, enrolling 146 *RAS/BRAF* wild-type CRC patients with initially unresectable CRLM. A stratified blocked randomization method was utilized to assign patients (1:1) to either the cetuximab plus FOLFOXIRI (*n* = 72) or cetuximab plus FOLFOX (*n* = 74) treatment arms. Stratification factors were tumor location (left versus right) and resectability (technically unresectable versus ≥5 metastases). The primary outcome was the objective response rate (ORR). Secondary outcomes included the median depth of tumor response (DpR), early tumor shrinkage (ETS), R0 resection rate, progression-free survival (PFS), overall survival (not mature at the time of analysis), and safety profile. Radiological tumor evaluations were conducted by radiologists blinded to the group allocation. Primary efficacy analyses were conducted based on the intention-to-treat population, while safety analyses were performed on patients who received at least 1 line of chemotherapy. A total of 14 patients (9.6%) were lost to follow-up (9 in the doublet arm and 5 in the triplet arm). The ORR was comparable following adjustment for stratification factors, with 84.7% versus 79.7% in the triplet and doublet arms, respectively (odds ratio [OR] 0.70; 95% confidence intervals [CI] [0.30, 1.67], Chi-square *p* = 0.42). Moreover, the ETS rate showed no significant difference between the triplet and doublet arms (80.6% (58/72) versus 77.0% (57/74), OR 0.82, 95% CI [0.37, 1.83], Chi-square *p* = 0.63). Although median DpR was higher in the triplet therapy group (59.6%, interquartile range [IQR], [50.0, 69.7] versus 55.0%, IQR [42.8, 63.8], Mann–Whitney *p* = 0.039), the R0/R1 resection rate with or without radiofrequency ablation/stereotactic body radiation therapy was comparable with 54.2% (39/72) of patients in the triplet arm versus 52.7% (39/74) in the doublet arm. At a median follow-up of 26.2 months (IQR [12.8, 40.5]), the median PFS was 11.8 months in the triplet arm versus 13.4 months in the doublet arm (hazard ratio [HR] 0.74, 95% CI [0.50, 1.11], Log-rank *p* = 0.14). Grade ≥ 3 events were reported in 47.2% (35/74) of patients in the doublet arm and 55.9% (38/68) of patients in the triplet arm. The triplet arm was associated with a higher incidence of grade ≥ 3 neutropenia (44.1% versus 27.0%, *p* = 0.03) and diarrhea (5.9% versus 0%, *p* = 0.03). The primary limitations of the study encompass the inherent bias in subjective surgical decisions regarding resection feasibility, as well as the lack of a centralized assessment for ORR and resection.

**Conclusions:**

The combination of cetuximab with FOLFOXIRI did not significantly improve ORR compared to cetuximab plus FOLFOX. Despite achieving an enhanced DpR, this improvement did not translate into improved R0 resection rates or PFS. Moreover, the triplet arm was associated with an increase in treatment-related toxicity.

**Trial Registration:**

ClinicalTrials.gov Identifier: NCT03493048.

## Abstract 2

De-Shen Wang and colleagues evaluate the efficacy and safety of cetuximab plus FOLFOXIRI versus cetuximab plus FOLFOX as a conversion regimen in CRC patients with initially unresectable colorectal liver metastases.

## Author summary

**Why was this study done?:**

- Liver metastasis is a common and significant challenge in colorectal cancer (CRC).
- Achieving resection through local treatments such as surgery, radiofrequency treatment, and stereotactic body radiation therapy is crucial for long-term survival. Therefore, identifying strategies to increase the conversion to resection rate in patients with initially unresectable colorectal liver metastases (CRLM) is paramount.
- Although the combination of an anti-EGFR with a doublet chemotherapy backbone has been established as the upfront conversion regimen in *RAS/BRAF* wild-type CRC patients with initially unresectable CRLM, several Phase II studies highlighted the substantial efficacy of a triplet chemotherapy backbone. Nevertheless, the added value of an intensified chemotherapy backbone in combination with cetuximab has not been explored in a randomized controlled trial at the time of this study.

**What did the researchers do and find?:**

- This prospective, open-label, randomized clinical trial aimed to assess the efficacy of upfront cetuximab plus FOLFOXIRI versus cetuximab plus FOLFOX in a cohort consisting only of *RAS/BRAF* wild-type CRC patients with initially unresectable CRLM.
- The study findings reveal that the objective response rates (ORRs) were comparable between the 2 treatment arms.
- Although a superior depth of tumor response was achieved in the cetuximab plus FOLFOXIRI treatment arm, this did not translate to improved resection rates or progression-free survival (PFS). Instead, this regimen was associated with increased treatment toxicities.

**What do these findings mean?:**

- Collectively, the TRICE study recommends selecting cetuximab plus FOLFOX as the first-line regimen for *RAS/BRAF* wild-type CRC patients with unresectable CRLM requiring conversion to resection.
- Major limitations of the study include the inherent bias in subjective surgical decisions regarding resection feasibility and the lack of centralized assessment for study endpoints.

## Introduction

Empirical evidence indicates that approximately 20% of colorectal cancer (CRC) patients harbor liver metastases at the time of diagnosis, with this ratio surging to around 50% as the disease progresses [1,2]. Given that liver metastasectomy confers a curative potential, with 5-year and 10-year survival rates of up to 33% and 23%, respectively, upfront systemic chemotherapy is typically indicated as approximately 75% to 80% of patients with colorectal liver metastases (CRLM) are initially deemed unresectable [3,4]. Importantly, complete resection with or without radiofrequency ablation (RFA) of initially unresectable liver lesions in metastatic CRC (mCRC) patients leads to a reported 5-year overall survival (OS) rate of up to 58%, comparable to that of patients with primary resectable metastases [5]. Consequently, it is paramount to develop more effective chemotherapy regimens and devise strategies to augment the proportion of patients eligible for liver metastasectomy.

Folprecht and colleagues [6] have previously established a robust correlation between the objective response rate (ORR) and resection rate in a series of retrospective and prospective studies of CRC patients with liver-only metastases. Additionally, multiple investigations have revealed that first-line FOLFOXIRI is superior to FOLFIRI or FOLFOX irrespective of the addition of bevacizumab in terms of ORR, R0 resection rate, and median OS in unresectable mCRC patients [7–9]. Notably, the CAIRO 5 study determined that FOLFOXIRI plus bevacizumab yielded added benefit in terms of progression-free survival (PFS), ORR, and R0/1 resection rates compared to FOLFOX or FOLFIRI plus bevacizumab in right-sided CRC subjects with initially unresectable CRLM and/or *RAS/BRAFV600E*-mutated primary tumors [10,11]. Although the combination of a monoclonal antibody against epidermal growth factor receptor (anti-EGFR) with a doublet chemotherapy backbone has been established as the first-line regimen for *RAS/BRAF* wild-type mCRC, several Phase II trials have corroborated the marked efficacy of an intensified triplet regimen combined with an anti-EGFR inhibitor [12–14]. Nevertheless, whether reinforcing the chemotherapy backbone can further boost the ORR and resection rate in patients with initially unresectable CRLM remains undetermined.

To that end, the TRICE study was conducted to determine whether first-line cetuximab plus FOLFOXIRI (triplet arm) can improve the ORR compared to cetuximab plus FOLFOX (doublet arm) in *RAS/BRAF* wild-type CRC patients with initially technically unresectable CRLM. Secondary study endpoints included the median depth of response (DpR), early tumor shrinkage (ETS), R0 resection rate, PFS, OS, and treatment-related adverse events.

## Methods

### Study design

The TRICE study is a prospective, open-label, multicenter, randomized clinical trial initiated by the Sun Yat-sen University Cancer Center (SYSUCC) and conducted in 7 medical centers across China from April 2018 to December 2022. The study was approved by the Human Research Ethics Committee of Sun Yat-sen University Cancer Center (approval number: B2018-008-01) and was undertaken under the principles detailed in the World Medical Association (WMA) Declaration of Helsinki and all relevant local laws and regulations. All individuals gave written informed consent prior to study enrollment. This study was registered under Clinical Trial Number (clinicaltrials.gov): NCT03493048.

### Patients

Patient eligibility criteria included *RAS/BRAF* wild-type CRC patients with histologically confirmed initially unresectable CRLM who were between 18 and 70 years of age, had not received first-line chemotherapy, and had an Eastern Cooperative Oncology Group (ECOG) performance status (PS) of 0–1 (indicating relatively good functional capacity with the ability to perform daily activities, although there may be some limitations in engaging in physically strenuous activities). Unresectability was defined as the presence of 5 or more metastatic lesions or metastases considered technically unresectable by an experienced multidisciplinary team (MDT) due to the following criteria: inadequate liver remnant post-resection (less than 30%), infiltration of all hepatic veins, infiltration of both hepatic arteries or both portal vein branches, consistent with the CELIM study [15,16]. Meanwhile, the exclusion criteria were as follows: patients with extrahepatic metastasis (except for unconfirmed lung or lymph node lesion with a size of less than 10 mm) and/or unresectable primary tumors, those with ECOG PS > 1, individuals with comorbidities or conditions that may impact treatment, and patients with known hypersensitivity to the drugs used in the study. For a comprehensive list of inclusion/exclusion criteria, please refer to S1 Data. Loss to follow-up included individuals who were no longer contactable or available for further participation in a study, either because they withdrew before the end of the planned follow-up period or for other reasons that rendered them unreachable. A total of 14 patients (9.6%) were lost to follow-up, distributed between the doublet and triplet arms, with 9 and 5 patients, respectively.

### Randomization and masking

A stratified blocked randomization method with the Interactive Web Response System (IWRS) was adopted to assign (1:1) a total of 146 CRC patients with initially unresectable CRLM to the cetuximab plus FOLFOXIRI or cetuximab plus FOLFOX treatment arms. Stratification factors were tumor location (left versus right) and resectability (technically unresectable versus ≥5 metastases). The initial dose of irinotecan was 130 mg/m^2^, according to the “3 + 3” dose-escalation design [17]. Given that no patients experienced dose-limiting toxicity, the dose was increased to 150 mg/m^2^. The cetuximab plus FOLFOX treatment arm received 500 mg/m^2^ intravenous cetuximab over 2 h, 85 mg/m^2^ oxaliplatin intravenous infusion over 3 h, 200 mg/m^2^ intravenous *l*-leucovorin (LV) over 2 h, bolus intravenous 400 mg/m^2^ fluorouracil (FU), followed by an infusion of 2,400 mg/m^2^ fluorouracil administered over 46 h, while the cetuximab plus FOLFOXIRI arm received an additional 150 mg/m^2^ intravenous irinotecan and no fluorouracil bolus injection. Both regimens were given every 2 weeks, and dose adjustments were permitted after a careful toxicity assessment. Radiological tumor evaluations were conducted by radiologists blinded to the group allocation after every 4 treatment cycles or more frequently if deemed necessary by the investigators. Liver metastases were assessed using contrast-enhanced magnetic resonance imaging (MRI) unless contraindications were present; if so, computed tomography (CT) was used. The CRC MDT, responsible for determining resectability, consists of accomplished professionals from diverse disciplines such as medical oncology, colorectal surgery, hepatobiliary surgery, radiation oncology, interventional radiology, medical imaging, pathology, and more. Each area of expertise is represented by a minimum of 2 to 3 physicians during each assessment, the majority of whom hold senior consultant positions and possess over 10 years of clinical experience in their respective fields. Patients were assessed for resectability after every 4 treatment courses or more frequently as deemed by the treating physician. Patients received at least 6 courses of conversion regimen before surgical resection was considered. Patients who successfully underwent local treatment did not enter a maintenance phase beyond the maximum of 12 perioperative treatment cycles. Conversely, for patients who did not achieve resectability after 12 treatment cycles, a maintenance treatment phase was initiated using cetuximab plus LV/FU until disease progression or unacceptable toxicities.

### Study endpoints

The primary outcome of this study was ORR, defined as the number of patients who exhibited a partial (target lesion reduction of at least 30% from the nadir) or complete response to systemic treatment, assessed every 8 weeks according to the RECIST version 1.1 criteria [18]. Secondary endpoints included the following: DpR (the relative change of the sum of the longest diameters of the target lesion from baseline in the absence of new lesions or progression of non-target lesions, assessed every 8 weeks), ETS (the number of patients with a target lesion reduction of a least 20% from the nadir following 4 treatment courses based on the RECIST version 1.1 criteria), R0 resection rate (the proportion of patients that underwent liver metastasectomy with or without RFA resulting in no residual tumor), PFS (the period between randomization to disease progression as defined by RECIST version 1.1 criteria or death, whichever came first or censored at the date of the last follow-up), OS (the time from randomization to death due to any cause or censored at the date of the last follow-up, not mature at the time of primary analysis), and treatment-related adverse events (classified according to the National Cancer Institute (NCI) Common Terminology Criteria for Adverse Events (CTCAE), version 4.03 criteria, (S1 Data)). In our study, the successful resection of liver metastases is considered a continuation of PFS, reflecting sustained survival without progression. The incidence and severity of surgery-related complications (perioperative mortality, intraoperative hemorrhage/transfusion, postoperative blood transfusion, and the number of blood units used) were assessed using the Clavien–Dindo classification of surgical complications [19].

### Statistical analysis

Primary efficacy analyses were conducted in the intention-to-treat population, safety analyses were performed in patients who received at least 1 dose of study treatment, and DpR was evaluated in patients who had at least 1 efficacy evaluation. With an expected ORR of 80% for the triplet arm and 60% for the doublet arm, the number of patients required before randomization was 128 using α = 10% (two-sided) and β = 0.20 (power 80%). Considering a dropout rate of 10%, at least 140 patients (70 in each treatment arm) were required for this study. Categorical data were represented as frequency (*n*), percentage (%), and 95% confidence interval (CI) where applicable. Continuous data were expressed using mean, standard deviation (SD), and median. Categorical data were analyzed using the Chi-square test, while continuous data were assessed using the Mann–Whitney two-sided test. Logistic regression models were established to estimate ORs and 95% CIs. The log-rank test was used to compare the treatment groups in terms of PFS, and a Cox proportional hazards model estimated hazard ratio (HR) with 95% CIs. Statistical adjustments were conducted, taking into account the stratification factors, and the proportional hazard assumption was assessed graphically by plotting log-log survival functions for the 2 treatment groups. To assess the treatment effect on ORR and PFS based on baseline patient characteristics, subgroup analyses were further conducted to investigate potential interactions. The Kaplan–Meier method was employed to estimate PFS with 95% CI. A stratified log-rank test was utilized to determine whether survival distribution was similar and to compare survival analysis indicators. The median follow-up was estimated using the reverse Kaplan–Meier method. Clinical variables (age, sex, tumor location, ECOG, primary tumor site, prior adjuvant therapy, time to metastases, number and size of metastases, resectability) were adjusted for the PFS comparison between those that achieved successful local treatment versus those that did not. All analyses were performed using IBM SPSS Statistics 26 and/or GraphPad Prism 9.0, and a two-sided *P* value < 0.1 was considered statistically significant for ORR, while a two-sided *P* value < 0.05 was used to determine significance for secondary endpoints.

## Results

### Patients

**Fig 1:**
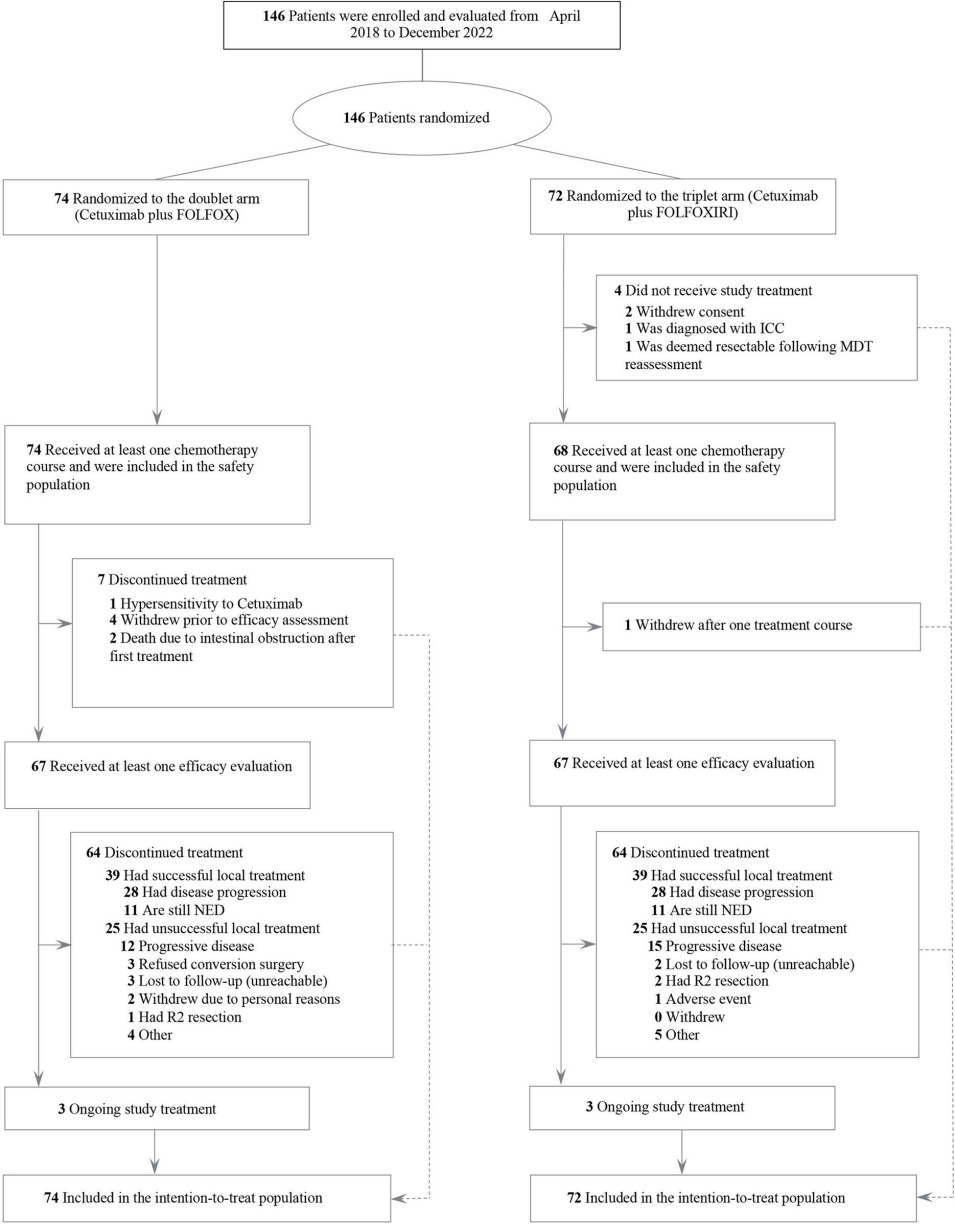
CONSORT diagram. The safety population consisted of patients who received at least 1 line of chemotherapy. Loss to follow-up included individuals who were no longer contactable or available for further participation in a study, either because they withdrew before the end of the planned follow-up period or for other reasons that rendered them unreachable. FOLFOX, fluorouracil, leucovorin, and oxaliplatin; FOLFOXIRI, modified fluorouracil, leucovorin, oxaliplatin, and irinotecan; ICC, intrahepatic cholangiocarcinoma; MDT, multidisciplinary team; NED, no evidence of disease.

A total of 146 patients were enrolled from April 2018 to December 2022 and randomly assigned to the cetuximab plus FOLFOXIRI (*n* = 72) or the cetuximab plus FOLFOX treatment arms (*n* = 74) (Fig 1). Among them, 142 patients (68 in the triplet arm and 74 in the doublet arm) received at least 1 course of chemotherapy and were included in our safety analysis. Meanwhile, 67 patients from each treatment arm received at least 1 efficacy evaluation, with a median of 8 (interquartile range (IQR), [7.0, 9.5]) preoperative treatment cycles. As detailed in Table 1, patient demographics and baseline characteristics were well balanced between the 2 treatment arms except for the size of liver metastases. The included patients were mostly aged ≤65 years old (82.2%), with a median age of 58 years old, and the majority had an ECOG PS score of 0 (93.8%). Besides, left-sided primary tumors (87.0%) and synchronous liver metastases (95.2%) were observed in most cases.

**Table 1:** Baseline clinical characteristics of patients in the ITT population.

Characteristics	Cetuximab plus FOLFOX (*n* = 74)	Cetuximab plus FOLFOXIRI (*n* = 72)
**Sex**		
Male	55 (74.3%)	54 (75.0%)
Female	19 (25.7%)	18 (25.0%)
**Age**		
Median (range, years)	58 (33.00–70.00)	58 (22.00–70.00)
≤65 years	63 (85.1%)	57 (79.2%)
>65 years	11 (14.9%)	15 (20.8%)
**ECOG PS**		
0	70 (94.6%)	67 (93.1%)
1	4 (5.4%)	5 (6.9%)
**Primary tumor site**		
Left	63 (85.1%)	64 (88.9%)
Right	11 (14.9%)	8 (11.1%)
**Previous treatment**		
Primary tumor resection	15 (20.3%)	13 (18.0%)
Prior adjuvant chemotherapy	1 (1.3%)	1 (1.4%)
Prior adjuvant radiotherapy	0 (0.0%)	2 (2.8%)
No previous treatment	58 (78.4%)	56 (77.8%)
**Synchronicity of liver metastases**		
Synchronous metastases*	69 (93.2%)	70 (97.2%)
Metachronous metastases	5 (6.8%)	2 (2.8%)
**Number of liver metastases**		
1–4	22 (29.7%)	18 (25.0%)
5–10	27 (36.5%)	24 (33.3%)
>10	25 (33.8%)	30 (41.7%)
**Size of metastases**		
<5 cm	18 (24.3%)	27 (37.5%)
5–10 cm	30 (40.5%)	33 (45.8%)
>10 cm	26 (35.1%)	12 (16.7%)
**Resectability**		
≥5 metastases	11 (14.9%)	14 (19.4%)
Technically unresectable	63 (85.1%)	58 (80.6%)
**LDH**		
Normal (<300 U/L)	23 (31.1%)	27 (37.5%)
Abnormal (>300 U/L)	51 (68.9%)	45 (62.5%)

* Liver metastases detected within 6 months of primary tumor diagnosis.

ITT, intention-to-treat; FOLFOX, fluorouracil, leucovorin, and oxaliplatin; FOLFOXIRI, modified fluorouracil, leucovorin, oxaliplatin, and irinotecan; ECOG PS, Eastern Cooperative Oncology Group performance status; LDH, lactate dehydrogenase.

### Efficacy

**Fig 2:**
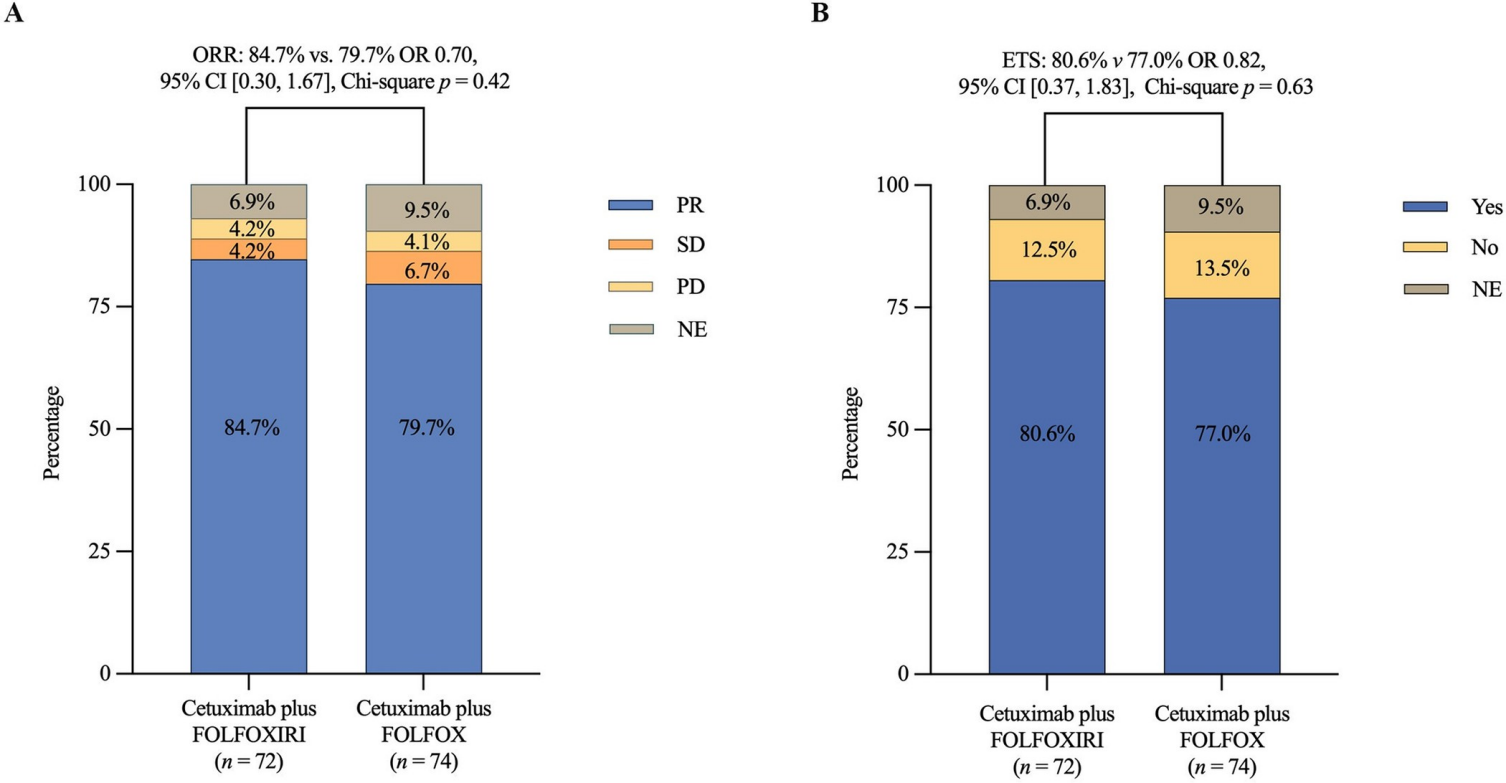
Tumor response comparisons between the 2 treatment arms in terms of (A) ORR and (B) ETS. Not evaluable refers to patients in the ITT population who did not have at least 1 efficacy assessment. FOLFOX, fluorouracil, leucovorin, and oxaliplatin; FOLFOXIRI, fluorouracil, leucovorin, oxaliplatin, and irinotecan; OR, odds ratio; PR, partial response; SD, stable disease; PD, progressive disease; NE, not evaluated; ORR, objective response rate; ETS, early tumor shrinkage.

**Fig 3:**
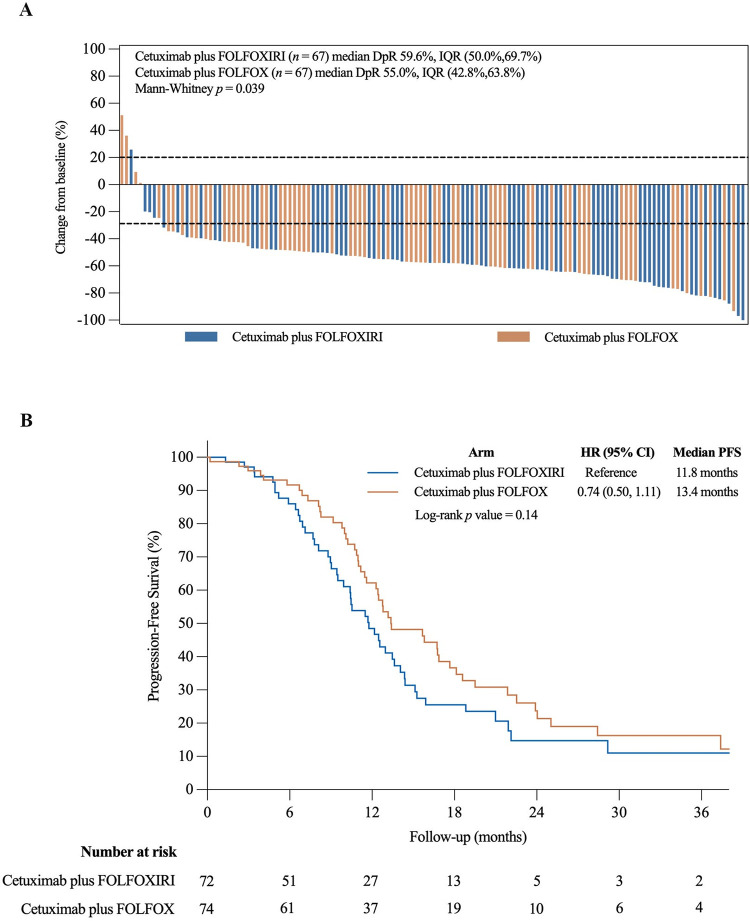
**(A) Waterfall plot depicting the depth of tumor response in patients that had at least 1 efficacy evaluation.** The blue and orange bars represent the percentage of tumor change from baseline for patients treated with cetuximab plus FOLFOXIRI and cetuximab plus FOLFOX, respectively. The dashed lines correspond to RECIST version 1.1 criteria, with PD indicating an increase of at least 20% in the sum of the diameters of target lesions from baseline, and PR defined as a minimum of 30% decrease in the sum of the diameters of target lesions from baseline. **(B) Progression-free survival in the ITT population.** FOLFOX, 5-fluorouracil, leucovorin, and oxaliplatin; FOLFOXIRI, 5-fluorouracil, leucovorin, oxaliplatin, and irinotecan; DpR, depth of response; IQR, interquartile range; ITT, intention-to-treat; PD, progressive disease; PR, partial response.

The ORR was 84.7% (61/72) in the triplet arm compared to 79.7% (59/74) in the doublet arm following adjustments for stratification factors (odds ratio [OR] 0.70, 95% CI [0.30, 1.67], Chi-square *p* = 0.42), and no patients from either group achieved complete responses (Fig 2A and S1 Table). The ETS rate was comparable between the triplet and doublet arms (80.6% (58/72) versus 77.0% (57/74), OR 0.82, 95% CI [0.37, 1.83], Chi-square *p* = 0.63) (Fig 2B). Further subgroup analysis of ORR revealed no marked difference between treatment arms and patient baseline clinical characteristics (S1 Fig). Interestingly, the patients in the triplet arm displayed a more significant change (Mann–Whitney *p* = 0.039) in terms of median DpR (*n* = 67, 59.6%, [IQR], [50.0, 69.7]) from baseline than those in the doublet arm (*n* = 67, 55.0%, IQR [42.8, 63.8]) (Fig 3A). Nonetheless, the R0/R1 resection rate with or without RFA/stereotactic body radiation therapy (SBRT) was comparable with 54.2% (39/72) of patients in the triplet arm versus 52.7% (39/74) in the doublet arm (S2 Table). Additionally, there was no substantial difference in postoperative complications between both treatment arms. Similarly, no marked differences in terms of surgical complications were observed. At a median follow-up of 26.2 months (IQR [12.8, 40.5]), the median PFS was 11.8 months for patients receiving cetuximab plus FOLFOXIRI versus 13.4 months for the cetuximab plus FOLFOX treatment arm (HR 0.74, 95% CI [0.50, 1.11], Log-rank *p* = 0.14) after accounting for stratification factors (Fig 3B). Moreover, PFS subgroup analysis did not reveal substantial differences between baseline characteristics and disease progression events (S2 Fig). Of note, successful local treatment (R0/R1 resection with or without RFA/SBRT) provided benefits in terms of PFS, with a significant improvement in PFS from 10.1 months to 13.6 months (HR 2.06, 95% CI [1.32, 3.20], Log-rank *p =* 0.001) following adjustment for clinical variables (S3 Fig). A total of 19 (13%) patients with right-sided tumors also received either the triplet (*n* = 8) or doublet (*n* = 11) regimen, achieving an ORR of 89.5% (17/19), R0/R1 resection with or without RFA/SBRT of 36.8% (7/19), and a PFS of 12.47 months 95% CI (10.67, 14.26).

### Safety

Treatment-related adverse events in the safety population are summarized in Table 2. Grade ≥ 3 events were reported in 47.2% (35/74) of patients in the doublet arm and 55.9% (38/68) of patients in the triplet arm. Patients in the triplet arm were more likely to suffer from grade 1–4 neutropenia (86.8% [59/68] versus 71.6% [53/74], *p* = 0.03) and diarrhea (73.5% [50/68] versus 23% [17/74], *p* < 0.001) than those in the doublet arm. Consistently, patients in the triplet arm had a higher incidence of grade ≥ 3 neutropenia (44.1% [30/68] versus 27.0% [20/74], *p* = 0.03) and diarrhea (5.9% [4/68] versus 0%, *p* = 0.03). Other common grade ≥ 3 adverse events (triplet versus doublet) included febrile neutropenia (4.4% [3/68] versus 4.1% [3/74]), stomatitis (10.3% [7/68] versus 13.5% [10/74]), and anemia (2.9% [2/68] versus 5.4% [4/74]). Moreover, 2 patients in the doublet arm suffered from severe hypersensitivities to cetuximab and oxaliplatin, respectively. Furthermore, in the doublet arm, 2 patients succumbed to complications of intestinal obstruction induced by the primary tumor.

**Table 2:** All-cause adverse events in the safety population.

	Cetuximab plus FOLFOX (*n* = 74)		Cetuximab plus FOLFOXIRI (*n* = 68)	
Adverse event	Grade 1–4	Grade 3–4	Grade 1–4	Grade 3–4
Any event	72 (97.3%)	35 (47.2%)	65 (95.5%)	38 (55.9%)
Anemia	41 (55.4%)	4 (5.4%)	42 (61.8%)	2 (2.9%)
Thrombocytopenia	11 (14.9%)	-	10 (14.7%)	2 (2.9%)
Neutropenia	53 (71.6%)	20 (27.0%)	59 (86.8%)	30 (44.1%)
Febrile neutropenia	-	3 (4.1%)	-	3 (4.4%)
Diarrhea	17 (23.0%)	-	50 (73.5%)	4 (5.9%)
Nausea	37 (50.0%)	-	41 (60.3%)	-
Vomiting	17 (23.0%)	-	20 (29.4%)	-
Fatigue	48 (64.9%)	1 (1.4%)	44 (64.7%)	-
Stomatitis	45 (60.8%)	10 (13.5%)	48 (70.6%)	7 (10.3%)
Acneiform rash	55 (74.3%)	1 (1.4%)	55 (80.9%)	-
Peripheral neuropathy	45 (60.8%)	-	44 (64.7%)	-
Hand-foot syndrome	6 (8.1%)	-	4 (5.9%)	-
Paronychia	7 (9.5%)	1 (1.4%)	6 (8.8%)	-
Hypoalbuminemia	31 (41.9%)	-	34 (50.0%)	-
Elevated ALT	40 (54.1%)	2 (2.7%)	39 (57.4%)	2 (2.9%)
Elevated AST	53 (71.6%)	2 (2.7%)	40 (58.8%)	2 (2.9%)
Elevated ALP	37 (50.0%)		36 (52.9%)	
Hypersensitivity to cetuximab	1 (1.4%)	1 (1.4%)	0 (0.0%)	-
Hypersensitivity to oxaliplatin	1 (1.4%)	1 (1.4%)	3 (4.4%)	-
Intestinal obstruction	2 (2.7%)	2 (2.7%)	-	-

The safety population consists of patients from the intention-to-treat cohort who received at least 1 line of chemotherapy. All adverse events were assessed using the NCI CTCAE, version 4.03 criteria. The highest grades for each parameter in each patient were selected.

ALT, alanine transaminase; AST, aspartate transaminase, ALP, alkaline phosphatase; NCI, National Cancer Institute; CTCAE, Common Terminology Criteria for Adverse Events.

## Discussion

The present study did not meet its primary endpoint, with no significant improvement in ORR following chemotherapy intensification. In addition, no marked differences in ETS, R0 resection rate, and PFS were observed between the 2 treatment arms. Nevertheless, the high efficacy exhibited by both treatment arms in terms of ORR, DpR, and ETS clearly demonstrates the advantages of combining cetuximab with chemotherapy as the conversion regimen of choice for *RAS/BRAF* wild-type CRLM patients.

Previous clinical trials indicated that FOLFOXIRI in combination with an anti-EGFR inhibitor can confer significant benefits. Notably, Assenat and colleagues [12] previously designed a single-arm study to investigate the efficacy of cetuximab plus a modified FOLFOXIRI regimen in advanced CRC and found that the overall ORR was 80.9% for *KRAS* wild-type patients, while PFS and OS were 9.5 and 24.7 months, respectively. Meanwhile, the MACBETH study reported an ORR of 71.6% and an R0 resection rate of 51.9% in CRC patients with liver-only metastases receiving cetuximab plus FOLFOXIRI [13]. Furthermore, in 2 independent Phase II studies, the combination of either panitumumab or cetuximab to FOLFOXIRI induced a significant benefit in terms of ORR, DpR, ETS, and secondary resection rate in *RAS* wild-type mCRC patients with initially technically unresectable CRLM [14,20]. The above outcomes are similar to those observed in our triplet arm, whereby an ORR of 84.7% and an R0/R1 resection rate with or without RFA/SBRT of 54.2% (39/72) were achieved, demonstrating the feasibility and efficacy of this treatment approach. However, there is a paucity of prospective randomized controlled trials to effectively compare the added value of using a triplet over a doublet chemotherapy backbone in combination with cetuximab in patients with unresectable CRLM, emphasizing the importance of the present study. Previously, the PRODIGE-14 Phase II randomized clinical trial investigated the value of chemotherapy intensification in combination with bevacizumab or cetuximab depending on *RAS/BRAF* status as a conversion regimen in initially unresectable liver-only metastases but did not meet its primary endpoint of increasing R0/R1 resection rate from 50% to 70% with the triplet regimen [21].

While we achieved a similar conclusion to the recent TRIPLETE study, whereby chemotherapy intensification in combination with an anti-EGFR antibody conferred limited benefit, there are several key differences between these 2 studies. The TRIPLETE study compared a modified schedule of FOLFOXIRI plus panitumumab to FOLFOX plus panitumumab as first-line therapy for *RAS/BRAF* wild-type mCRC patients, the majority of whom exhibited multiple metastatic sites (52%), with only 38% of patients having liver-only disease [22]. In both groups, chemotherapy plus panitumumab was administered up to 12 cycles, followed by maintenance with FU/LV and panitumumab. In contrast, the TRICE study exclusively enrolled *RAS/BRAF* wild-type CRC patients with technically unresectable CRLM, aiming to evaluate the efficacy of cetuximab in combination with an intensified chemotherapy backbone in CRC patients who could potentially achieve conversion to resection. Moreover, no maintenance phase was initiated following the maximum of 12 cycles in both treatment arms, unless patients did not reach resectability. While the ORR, DpR, and PFS between the 2 studies demonstrated substantial similarity, the TRICE study documented superior R0 resection rates. A potential factor contributing to this trend may stem from differences in the proportion of patients exclusively presenting with liver metastases across the 2 studies. Indeed, our study utilized a different stratification strategy based on resectability status (technically unresectable versus ≥5 metastases) that included more patients that were technically initially unresectable (82.9%) in contrast to the CELIM study (55.0%) [15,16].

Although cetuximab plus FOLFOXIRI yielded a better DpR in the present study, this did not translate into a meaningful improvement in the R0 resection rate. Currently, there is no evidence supporting the usefulness of a higher DpR in specific patient populations, such as those with liver metastases involving all major hepatic veins, although it is plausible that significant tumor downsizing may facilitate successful surgical resection. In addition, previous studies have demonstrated a potential correlation between deeper tumor response and longer OS [23–25]. Therefore, further analysis after OS maturation and patient stratification is warranted to determine whether the improved DpR in our triplet arm connotes better overall survival. In line with the CAIRO 5 study, our findings suggest that patients who underwent successful local treatment experienced a longer median PFS compared to those who did not achieve R0/R1 resections, hinting at the importance of devising conversion to resection strategies in patients with initially unresectable CRLM [10]. Nevertheless, it is important to acknowledge the potential influence of survivor bias on these results, since patients who underwent successful local treatment and subsequently achieved better PFS may inherently possess favorable prognostic factors in addition to a preexisting period of PFS prior to surgical intervention as opposed to those who did not. Therefore, there is a need for sufficiently powered randomized controlled trials to further validate this finding and address these limitations. Right-sided CRC patients (*n* = 19) also achieved a high ORR (89.5%) and ETS rate (73.7%) in both arms but exhibited a lower conversion rate (36.8%) compared to left-sided CRC patients. Presently, the use of an anti-EGFR monoclonal antibody in right-sided CRC patients whose treatment goal is conversion to resection is supported by several studies [14,26,27]. Conversely, a subgroup analysis within the PARADIGM study recently indicated that right-sided CRC patients treated with panitumumab displayed a lower response rate and comparable R0 resection rate compared to bevacizumab [28]. Consequently, further prospective randomized trials are imperative to validate these findings and elucidate the optimal therapeutic strategy for this specific patient cohort.

A triplet chemotherapy backbone is typically associated with an inferior safety profile compared to a doublet chemotherapy regimen. Herein, the triplet arm was associated with a higher incidence of diarrhea (73.5% versus 23%, chi-square, *p* < 0.001) and neutropenia (86.8% versus 71.6%, chi-square, *p* = 0.03) compared to the doublet arm, in line with the TRIPLETE study. However, we documented a lower incidence of grade ≥ 3 diarrhea (5.9%) in the triplet arm versus the 23% reported in the TRIPLETE study despite similar irinotecan and fluorouracil concentrations used. In the FOCULM trial conducted in *RAS/BRAF* wild-type mCRC patients of Asian descent, 165 mg/m^2^ irinotecan was utilized, and only 7.5% of patients experienced grade ≥ 3 diarrhea, aligning with the lower incidence of grade ≥ 3 diarrhea we reported in our study when 150 mg/m^2^ irinotecan was used. Growing evidence suggests that *UGT1A1* polymorphisms can elevate the risk of severe irinotecan-related toxicities, particularly grade ≥ 3 myelosuppression and diarrhea [29–31]. Considering that the prevalence of the UGT1A1*28 genotype in the Asian population is nearly 2.5 times lower than in the white population (16% versus 39%) [32], this discrepancy could potentially contribute to the observed contrast. Recently, the AXEPT study, which was conducted across 98 hospitals in Japan, South Korea, and China, reported only 7.7% (50/650) of patients with polymorphisms homozygous for UGT1A1*6 or UGT1A1*28 or double heterozygous for UGT1A1*6 and UGT1A1*28 [33]. Herein, none of the patients in the cetuximab plus FOLFOXIRI arm exhibited polymorphisms for UGT1A1*6 or UGT1A1*28, providing additional insight into the reduced incidence of grade ≥ 3 diarrhea.

Nonetheless, the present study has several limitations, including selecting ORR as a surrogate endpoint instead of using overall survival, which is considered the gold standard for assessing treatment efficacy in clinical trials. Whether alternative primary endpoints to OS, such as DpR and time to surgical failure, could be superior to ORR remains to be determined. Moreover, stratified balanced sampling of the technically unresectable subgroup was not conducted, resulting in a higher number of patients with more than 10 metastases in the triplet arm (41.7% versus 33.8%), potentially leading to the longer but not statistically longer PFS in the doublet arm. Lastly, inevitable bias in subjective surgical decisions regarding resection feasibility may have influenced our study outcomes, suggesting the need for a centralized surgical and ORR assessment in the future.

Taken together, our findings underscore the recommendation to prioritize cetuximab plus FOLFOX as the preferred regimen for unresectable *RAS/BRAF* wild-type CRLM patients undergoing conversion to resection. Although cetuximab in combination with FOLFOXIRI can significantly increase the depth of tumor response for *RAS/BRAF* wild-type patients with initially unresectable CRLM, this improvement did not translate into superior ORR or R0 resection rates. Instead, this treatment intensification was associated with increased treatment-related toxicity.

## Supporting information

S1 Fig Subgroup analysis of the ORR based on the baseline patient clinical factors with respective *p* values for interaction. ECOG, Eastern Cooperative Oncology Group; OR, odds ratio; CI, confidence interval; mets., metastases; LDH, Lactate dehydrogenase.
(TIF)

S2 Fig Subgroup analysis of PFS based on the baseline patient clinical factors with respective *p* values for interaction. ECOG, Eastern Cooperative Oncology Group; HR, hazard ratio; CI, confidence interval; mets., metastases; LDH, Lactate dehydrogenase.
(TIF)

S3 Fig Progression-free survival of patients with successful versus unsuccessful local treatment. HR, hazard ratio; PFS, progression-free survival; CI, confidence interval.
(TIF)

S1 Table Best objective response rate as per RECIST version 1.1. *Patients in the ITT population that did not have at least 1 efficacy assessment. RECIST, Response Evaluation Criteria in Solid Tumors; FOLFOX, fluorouracil, leucovorin, and oxaliplatin; FOLFOXIRI, modified fluorouracil, leucovorin, oxaliplatin, and irinotecan.
(DOCX)

S2 Table Postoperative complications and R0 resection rates. *Included patients who needed blood transfusions and those who needed total parenteral nutrition due to intestinal obstruction. ^#^Included patients who needed stent placement due to post-hepatectomy bile duct leaks. FOLFOX, fluorouracil, leucovorin, and oxaliplatin; FOLFOXIRI, modified fluorouracil, leucovorin, oxaliplatin, and irinotecan; RFA, radiofrequency ablation; SBRT, Stereotactic body radiotherapy.
(DOCX)

S1 Data Study protocol. (DOCX)

S2 Data Consort checklist. (DOCX)

## Abbreviations

CI: confidence interval
CRC: colorectal cancer
CRLM: colorectal liver metastases
CT: computed tomography
CTCAE: common terminology criteria for adverse events
DpR: depth of tumor response
ECOG: Eastern Cooperative Oncology Group
EGFR: epidermal growth factor receptor
ETS: early tumor shrinkage
FU: fluorouracil
FOLFOX: fluorouracil and oxaliplatin
FOLFOXIRI: fluorouracil oxaliplatin irinotecan
HR: hazard ratio
IQR: interquartile range
IWRS: Interactive Web Response System
LV: leucovorin
mCRC: metastatic CRC
MDT: multidisciplinary team
MRI: magnetic resonance imaging
NCI: National Cancer Institute
OR: odds ratio
ORR: objective response rate
OS: overall survival
PFS: progression-free survival
PS: performance score
RECIST: response evaluation criteria in solid tumors
RFA: radiofrequency ablation
SBRT: stereotactic body radiation therapy
SD: standard deviation
UGT1A1: UDP glucuronosyltransferase 1 family polypeptide A1
WMA: World Medical Association
