# Supplementary figures and images for: Cetuximab plus FOLFOXIRI versus cetuximab plus FOLFOX as conversion regimen in RAS/BRAF wild-type patients with initially unresectable colorectal liver metastases (TRICE trial): A randomized controlled trial
Source: PLoS Med. 2024 May 10;21(5):e1004389. doi: 10.1371/journal.pmed.1004389 (PMC11086847; doi:10.1371/journal.pmed.1004389)

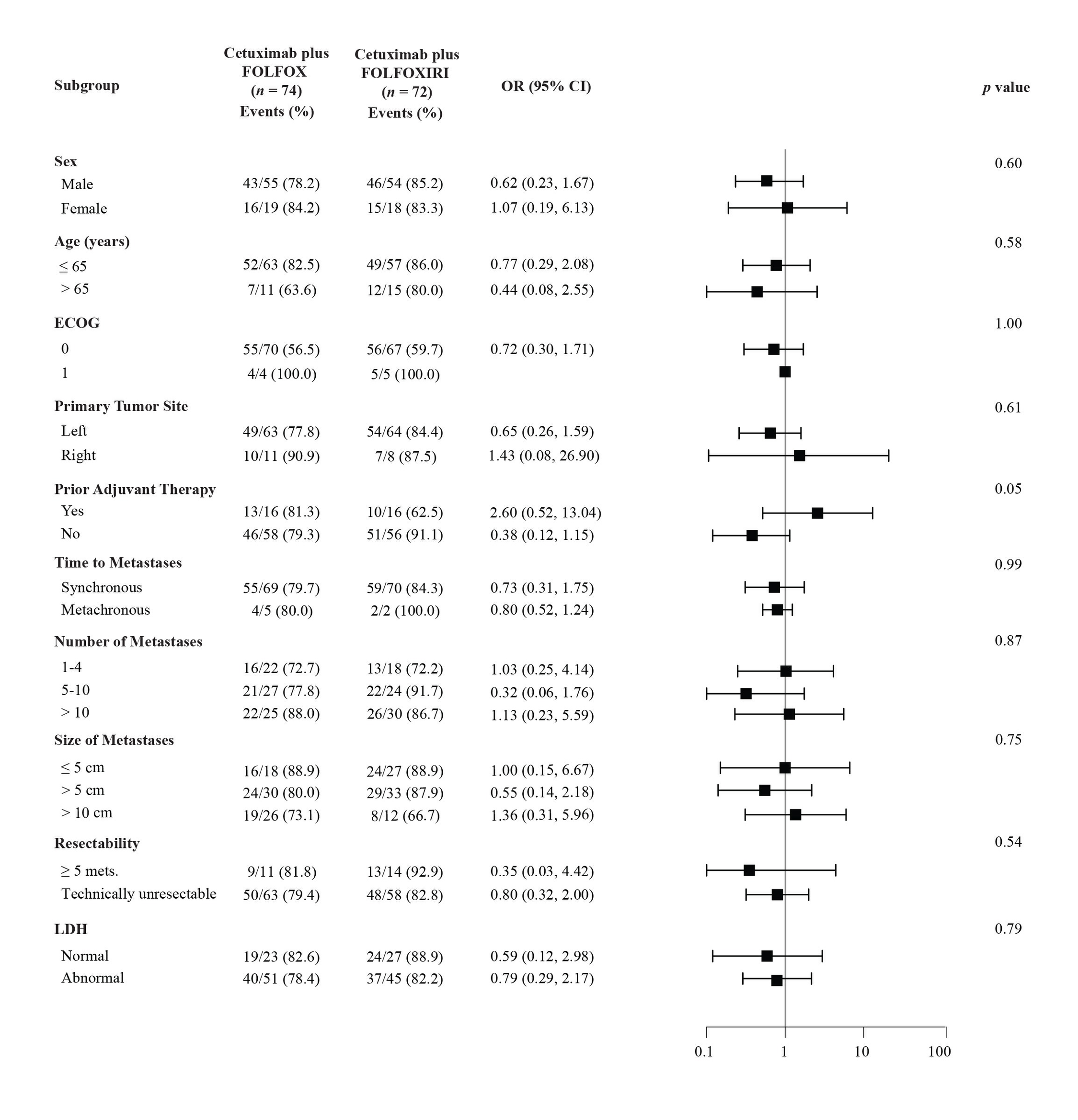

Supplement: S1 Fig — ECOG, Eastern Cooperative Oncology Group; OR, odds ratio; CI, confidence interval; mets., metastases; LDH, Lactate dehydrogenase. (TIF) [file pmed.1004389.s001.tif]

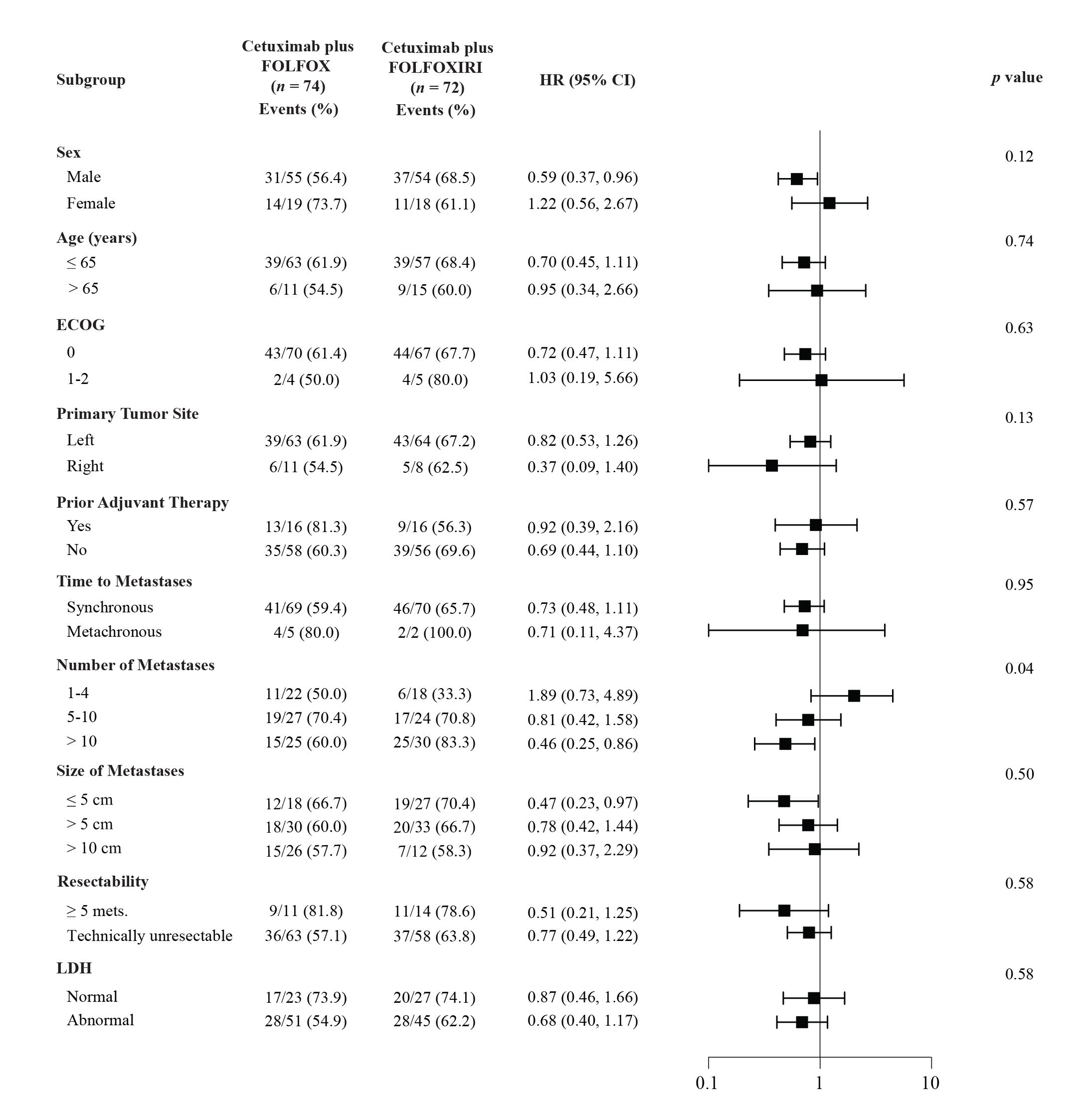

Supplement: S2 Fig — ECOG, Eastern Cooperative Oncology Group; HR, hazard ratio; CI, confidence interval; mets., metastases; LDH, Lactate dehydrogenase. (TIF) [file pmed.1004389.s002.tif]

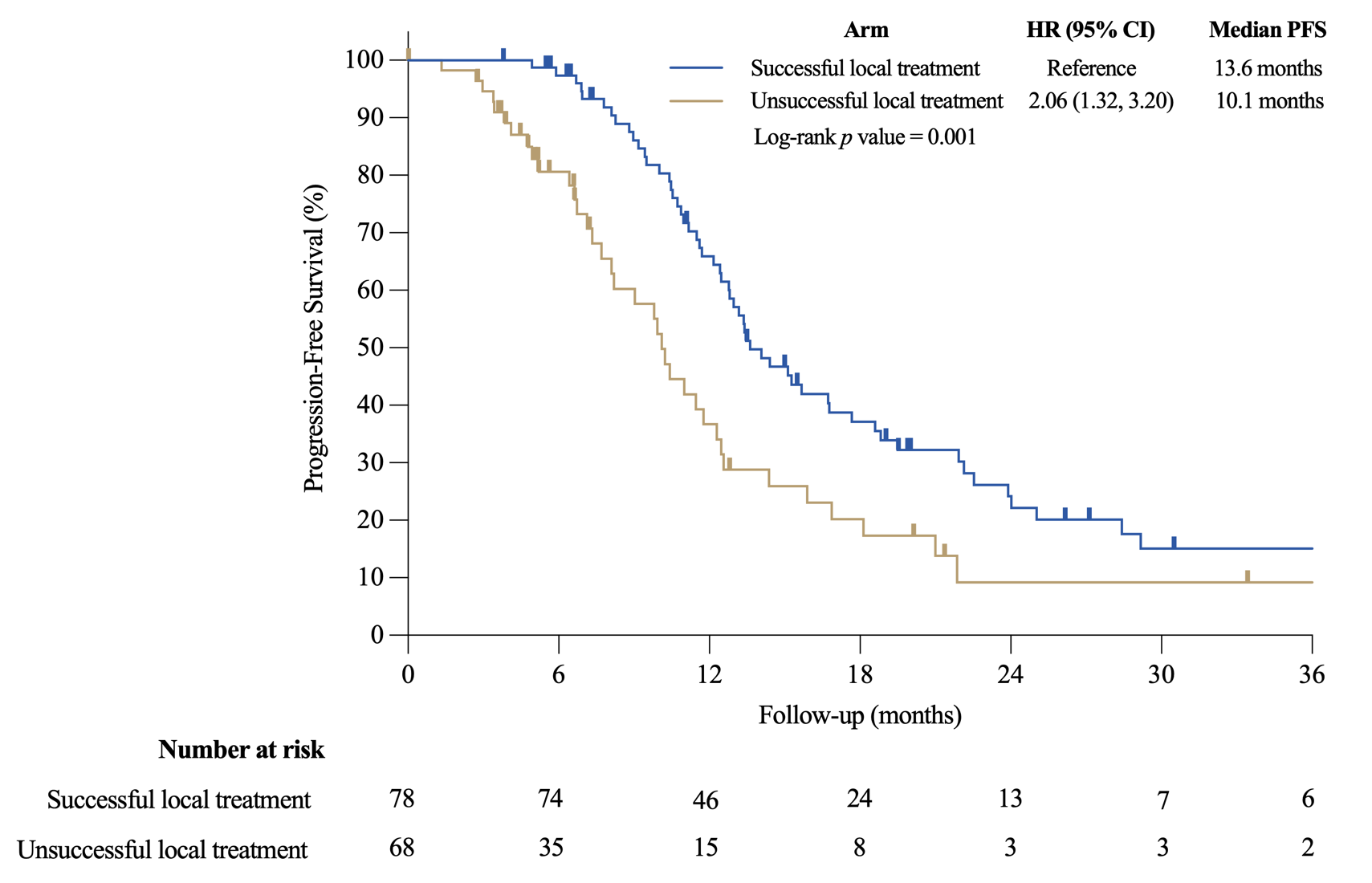

Supplement: S3 Fig — HR, hazard ratio; PFS, progression-free survival; CI, confidence interval. (TIF) [file pmed.1004389.s003.tif]
