# Supplementary material for: Cetuximab plus FOLFOXIRI versus cetuximab plus FOLFOX as conversion regimen in RAS/BRAF wild-type patients with initially unresectable colorectal liver metastases (TRICE trial): A randomized controlled trial
Source: PLoS Med. 2024 May 10;21(5):e1004389. doi: 10.1371/journal.pmed.1004389 (PMC11086847; doi:10.1371/journal.pmed.1004389)
Supplement: S1 Table — *Patients in the ITT population that did not have at least 1 efficacy assessment. RECIST, Response Evaluation Criteria in Solid Tumors; FOLFOX, fluorouracil, leucovorin, and oxaliplatin; FOLFOXIRI, modified fluorouracil, leucovorin, oxaliplatin, and irinotecan. (DOCX) [file pmed.1004389.s004.docx]

**S1 Table. Best objective response rate as per RECIST version 1.1.**

|  | **Cetuximab plus FOLFOX**  **(*n* = 74)** | **Cetuximab plus FOLFOXIRI**  **(*n* = 72)** |
| --- | --- | --- |
| Complete response | 0 | 0 |
| Partial response | 59 (79.7%) | 61 (84.7%) |
| Stable disease | 5 (6.7%) | 3 (4.2%) |
| Progressive disease | 3 (4.1%) | 3 (4.2%) |
| Not evaluated^*^ | 7 (9.5%) | 5 (6.9%) |
| Objective response rate | 59 (79.7%) | 61 (84.7%) |

^*^Patients in the ITT population that did not have at least one efficacy assessment. Abbreviations: RECIST, Response Evaluation Criteria in Solid Tumors.; FOLFOX, fluorouracil, leucovorin, and oxaliplatin; FOLFOXIRI, modified fluorouracil, leucovorin, oxaliplatin, and irinotecan.
