# Supplementary material for: Cetuximab plus FOLFOXIRI versus cetuximab plus FOLFOX as conversion regimen in RAS/BRAF wild-type patients with initially unresectable colorectal liver metastases (TRICE trial): A randomized controlled trial
Source: PLoS Med. 2024 May 10;21(5):e1004389. doi: 10.1371/journal.pmed.1004389 (PMC11086847; doi:10.1371/journal.pmed.1004389)
Supplement: S2 Table — *Included patients who needed blood transfusions and those who needed total parenteral nutrition due to intestinal obstruction. #Included patients who needed stent placement due to post-hepatectomy bile duct leaks. FOLFOX, fluorouracil, leucovorin, and oxaliplatin; FOLFOXIRI, modified fluorouracil, leucovorin, oxaliplatin, and irinotecan; RFA, radiofrequency ablation; SBRT, Stereotactic body radiotherapy. (DOCX) [file pmed.1004389.s005.docx]

**S2 Table. Postoperative complications and R0 resection rates.**

|  | **Cetuximab plus FOLFOX (*n* = 74)** | **Cetuximab plus FOLFOXIRI (*n* = 72)** | ***p* value** |
| --- | --- | --- | --- |
| Postoperative complications | 22 (29.7%) | 24 (33.3 %) | 0.64 |
| Clavien Dindo grade 2^*^ complications | 7 (9.5 %) | 5 (6.9 %) | 0.58 |
| Clavien Dindo grade 3A^#^ complications | 2 (2.7%) | 1 (1.38%) | 0.57 |
| R0 resection +/- RFA | 38 (51.4%) | 37 (51.4 %) | 1.00 |
| R0/R1 resections +/- RFA/SBRT | 39 (52.7%) | 39 (54.2%) | 0.86 |

^*^Included patients who needed blood transfusions and those who needed total parenteral nutrition due to intestinal obstruction. ^#^Included patients who needed stent placement due to post-hepatectomy bile duct leaks. Abbreviations: FOLFOX, fluorouracil, leucovorin, and oxaliplatin; FOLFOXIRI, modified fluorouracil, leucovorin, oxaliplatin, and irinotecan; RFA, radiofrequency ablation; SBRT, Stereotactic body radiotherapy.
