# Supplementary material for: Cetuximab plus FOLFOXIRI versus cetuximab plus FOLFOX as conversion regimen in RAS/BRAF wild-type patients with initially unresectable colorectal liver metastases (TRICE trial): A randomized controlled trial
Source: PLoS Med. 2024 May 10;21(5):e1004389. doi: 10.1371/journal.pmed.1004389 (PMC11086847; doi:10.1371/journal.pmed.1004389)
Supplement: S1 Data — (DOCX) [file pmed.1004389.s006.docx]

The TRICE Study:

Cetuximab Plus FOLFOXIRI versus Cetuximab Plus FOLFOX as initial treatment for unresectable colorectal with liver metastases patients

**PROTOCOL**

**第1.5版**

SPONSOR: Sun Yat-sen University

**2021年02月07日CONTENTS**

[**1. Study background 10**](#_Toc112413171)

[**2. Study objectives 14**](#_Toc112413172)

[2.1 Primary objective 14](#_Toc112413173)

[2.2 Secondary objectives 14](#_Toc112413174)

[**3. Study population** 14](#_Toc112413175)

[3.1 Inclusion criteria 15](#_Toc112413176)

[**3.2 Exclusion criteria 16**](#_Toc112413177)

[**4. Estimated sample size/randomization 17**](#_Toc112413178)

[**5. Study regimen** 17](#_Toc112413179)

[5.1 Study steps and procedures 18](#_Toc112413180)

[5.2 Dosing regimens and treatment courses 21](#_Toc112413181)

[**6. Chemotherapy dose adjustment and concomitant medication** 22](#_Toc112413182)

[6.1 Overview 22](#_Toc112413183)

[6.2 Dose modifications due to hematologic toxicity 23](#_Toc112413184)

[6.3 Dose modifications due to non-hematologic toxicity 24](#_Toc112413185)

[6.4 Management of adverse reactions due to cetuximab 25](#_Toc112413186)

[6.5 Management of other toxicities 27](#_Toc112413187)

[6.6 Concomitant medications: 28](#_Toc112413188)

[**7. Efficacy evaluation** 28](#_Toc112413190)

[7.1 Indicators of effectiveness evaluation 28](#_Toc112413191)

[7.2 Indicators of safety evaluation 29](#_Toc112413192)

[**8. Screening and baseline visit** 29](#_Toc112413193)

[**9. Assessment during the study 31**](#_Toc112413194)

[**9.1 Pre-chemotherapy assessment 31**](#_Toc112413195)

[9.2 Surgery and related assessments 32](#_Toc112413196)

[9.3 Post-treatment follow-up 32](#_Toc112413197)

[9.4 Discontinuation and withdrawal criteria 33](#_Toc112413198)

[**10. Statistical analysis** 33](#_Toc112413199)

[10.1 Study population 33](#_Toc112413200)

[10.2 Statistical analysis methods 34](#_Toc112413201)

[10.3 Analysis plan 34](#_Toc112413202)

[10.4 Stratified analyses 34](#_Toc112413203)

[11. Adverse events reporting and recording 35](#_Toc112413204)

[11.1 Definition 35](#_Toc112413205)

[11.2 Scope of expected adverse events 36](#_Toc112413206)

[11.3 Relevant indicators for determining the causal relationship between adverse events and drugs 36](#_Toc112413207)

[11.4 Management of adverse events 38](#_Toc112413208)

[APPENDIX I Karnofsky Performance Status 42](#_Toc112413209)

[APPENDIX II Child-Pugh Classification 43](#_Toc112413210)

[APPENDIX III Common Terminology Criteria for Adverse Events（ CTCEA V4.03,2010-06-14） partial content 44](#_Toc112413211)

[APPENDIX IV Response Evaluation Criteria in Solid Tumors 193](#_Toc112413212)

[APPENDIX V Classification of Surgical Complications：Clavien–Dindo Grade](#_Toc517797327) 215**Summary of Research Protocol**

| Study | The TRICE Study: Cetuximab Plus FOLFOXIRI versus Cetuximab Plus FOLFOX as initial treatment for unresectable colorectal with liver metastases patients |
| --- | --- |
| Study design | prospective, open-label, multicentre phase II randomized trial |
| Sites | Sun Yat-sen University etc |
| Protocol | FOLFOXIRI plus Cetuximab versus mFOLFOX plus Cetuximab |
| Objectives | Primary Objectives：Overall Response Rate（ORR）  Secondary Objectives：Depth of response (DpR)、 R0/resection rate、Early tumor shrinkage (ETS)、Progression-free Survival（PFS）、Overall Survival（OS）、Safety profile; |
| Inclusion criteria | Patients must meet the following study criteria:   - Age ≥ 18 years and ≤ 70 years. - Histologically confirmed colorectal adenocarcinoma. - Liver metastasis confirmed by imaging or pathology. - The multidisciplinary team (MDT) determines that the liver metastases are unresectable, which is specifically defined as ① metastatic lesions ≥ 5; ② ineligible for R0 resection; ③ expected insufficient residual liver volume after resection; ④ unable to preserve all three hepatic veins after resection, unable to ensure that the blood flow and bile ducts of the residual liver into and out of the liver could be preserved, and unable to preserve the adjacent two liver segments. Patients who meet any of the above criteria can be determined as having initially unresectable liver metastases. - Patients with wild-type RAS (KRAS and NRAS exons 2,3, and 4) and BRAF codon 600. - No prior treatment for liver metastases, including chemotherapy, surgery, radiotherapy, transcatheter arterial chemoembolization (TACE), and targeted therapy. - Absence of extrahepatic metastasis confirmed by CT, MRI or PET/CT (if necessary) (enrollment can be considered if there is a lung or lymph node lesion less than 10 mm, which is difficult to determine metastases). - Normal hematologic function (platelets > 90 × 10^9^/L; leukocytes > 3 × 10^9^/L; neutrophils > 1.5 × 10^9^/L). - Serum bilirubin ≤ 1.5 times the upper limit of normal (ULN) and transaminases ≤ 5 times ULN. - No ascites, normal coagulation function, albumin ≥ 35 g/L. - Liver function: Child-Push score: Class A - Serum creatinine < ULN, or calculated creatinine clearance > 50 ml/min (using the Cockcroft-Gault formula). - ECOG score 0-1. - Life expectancy > 3 months. - Sign written informed consent. - Willing and able to be followed up until death or end of study or study termination. |
| Exclusion criteria | Patients who meet any of the following criteria will be excluded from the study:   - Presence of any extrahepatic metastasis and/or primary tumor that cannot be resected with radical surgery. - Serious arterial embolism or ascites. - Have bleeding tendency or coagulation disorder. - Have hypertensive risk or hypertensive encephalopathy. - Serious uncontrolled systemic complications such as infection or diabetes. - Clinically significant cardiovascular disease such as cerebrovascular accident (within 6 months prior to enrollment), myocardial infarction (within 6 months prior to enrollment), uncontrolled hypertension despite appropriate medical treatment. Unstable angina, congestive heart failure (NYHA class 2-4), cardiac arrhythmia requiring medication. - History or physical evidence of central nervous system disease (e.g., primary brain tumor, epilepsy uncontrolled by standard of care, any history of brain metastases or stroke). - History of other malignancies (except basal cell carcinoma of the skin and/or carcinoma in situ of the cervix after radical surgery) within the past 5 years. - Treatment with any ongoing investigational drug within the last 28 days prior to the study. - Any residual toxicity from prior chemotherapy (except alopecia), such as peripheral neuropathy ≥ NCI CTC v4.03 Grade 2, will not be considered for oxaliplatin-containing regimen. - Hypersensitivity to any drug in the study. - Pregnant and lactating women. - Women of childbearing age (< 2 years after menstruation) or men of childbearing potential who are not using or refuse to use effective non-hormonal contraception (intrauterine contraceptive ring, barrier contraceptives combined with spermicidal gel, or surgical sterilization). - Unable or unwilling to comply with the study protocol. - Patients with any other diseases, dysfunction caused by metastatic lesions, or suspected disease found by physical examination, indicating possible contraindications to the use of the investigational drug or putting the patients at high risk of treatment-related complications. |
| Sample size | We expect an ORR of 80% in patients treated with cetuximab plus FOLFOXIRI regimen and 60% in patients treated with cetuximab plus FOLFOX regimen. With a type 1 error probability two-sided α = 10% and a power β = 80%, a total of 128 patients are required to be enrolled into randomization. Considering a dropout rate of 10%, a total of 140 patients are planned to be enrolled in this study, with 70 patients in each group. |
| Study steps and procedures | 1. The screening procedure begins after the patients sign the written informed consent. 2. CT and/or MRI scans (with PET-CT where available) are used to assess liver metastases and to exclude extrahepatic metastases. 3. An MDT meeting is held prior to chemotherapy and the resectability of metastases is assessed and determined based on the given criteria. The liver surgeon performs a comprehensive assessment of each patient’s baseline liver metastases, record in detail the number and size of liver metastases, the liver segment involved, whether the hepatic vein, portal vein, and inferior vena cava are involved, and explain in detail why each patient is considered initially unresectable. 4. Eligible patients at screening are randomly assigned by the study physician to receive either cetuximab plus FOLFOXIRI triplet regimen or cetuximab plus FOLFOX doublet regimen. 5. CT and/or MRI scans are performed every 4 cycles after chemotherapy, with additional examinations if necessary, and the tumor is assessed according to RECIST v1.1 criteria; MDT discussion is held regularly to determine the resectability of patients’ liver metastases. The reason why each patient can be converted to surgically resectable is also specified. 6. In this study, 8 courses (no less than 6 courses) of conversion chemotherapy are recommended, and some patients also need to appropriately prolong the treatment course. This is mainly because that this study includes patients with unresectable colorectal liver metastases, who usually have a large number of liver metastases, large lesions, with high CRS score. Current clinical practice recommends surgical resection after adequate chemotherapy to achieve more benefit. 7. For the treatment of liver metastases, surgical resection with or without intraoperative radiofrequency treatment can be used. For the lesions that cannot be resected or treated with radiofrequency during surgery but are suitable for SBRT treatment, SBRT should be added after surgery. The timing of resection of primary intestinal lesions can be determined by the investigator according to the patient’s condition. For patients with liver metastases converted to surgical resectable, primary intestinal disease can be resected before chemotherapy, or can be resected at the same time or in stages with liver metastases after successful conversion. For patients with rectal cancer, local rectal radiotherapy can be performed when needed, and long-course or short-course radiotherapy can be used. 8. Patients with liver metastases can receive chemotherapy alone with or without cetuximab as adjuvant chemotherapy after surgery. Patients in the cetuximab + mFOLFOXIRI regimen group can receive doublet regimen chemotherapy after surgery. Patients should receive tumor assessment before postoperative adjuvant chemotherapy, including at least CEA, liver MRI scan + enhanced scan, and chest and lower abdomen pelvic CT (if necessary). Patients will receive up to a total of 12 courses of chemotherapy before and after surgery, and treatment will be interrupted in the event of disease progression, intolerable adverse reactions, or patient refusal. 9. For patients who cannot undergo surgery or are unwilling to receive surgery but can receive radiofrequency treatment, radiofrequency treatment can be given. The total number of planned chemotherapy courses treated before and after radiofrequency treatment is 12 courses. 10. For patients who received adequate chemotherapy and whose tumor is controlled (PR or SD achieved) but still cannot undergo resection or radiofrequency, the treatment regimen can be changed for conversion therapy, and once the conversion is successful, surgical treatment and/or radiofrequency therapy can be performed. These patients may also discontinue chemotherapy or enter maintenance chemotherapy. 11. Patients who have disease progression during chemotherapy can discuss with the investigator to decide whether to receive further palliative treatment. For patients with unresectable liver metastases after chemotherapy, whether primary lesion resection should be performed can be decided according to the patient’s wishes and specific circumstances.   Patients who have completed the treatment specified in the protocol or have not completed the treatment due to intolerable toxicity, but have not experienced tumor progression, are followed up every 3 months, including physical examination and imaging examination until tumor progression. For patients with tumor progression, follow-up will be performed by phone every 3 months until death. The following information should be collected as much as possible during follow-up: subsequent anti-tumor treatment and survival data. Patients with preoperative disease progression should withdraw from the study and enter into the follow-up period as well |
| Evaluation Criterion and time | Unresectable criteria included: ① ≥5 metastatic lesions;② R0 resection could not be performed;③ Insufficient residual liver volume is expected after resection;④ None of the three hepatic veins could be preserved after resection, which could not ensure that the blood flow into and out of the liver and the bile duct of the residual liver could be preserved, and the two adjacent hepatic segments could not be preserved. Patients who meet any of the above criteria can be judged as initially unresectable liver metastases.  Evaluation criteria of chemotherapy efficacy: RECIST V1.1 criteria  Evaluation criteria of chemotherapy adverse reactions: NCI CTC:4.03  Evaluation criteria of surgical complications: Clavien-Dindo grading system |
| Statistical analysis | Statistical software SAS 9.1.3 and GraphPad PRISM 9.0 were used to analyze the data |
| Expected Study progress | Enrollment and preliminary efficacy evaluation were completed by the end of December 2022 |

**The TRICE Study: Cetuximab Plus FOLFOXIRI versus Cetuximab Plus FOLFOX as initial treatment for unresectable colorectal with liver metastases patients**

## 1. Study background

Colorectal cancer (CRC) is one of the most common malignant tumor in China and which is the fifth most common cancer. At present, the incidence and mortality of CRC in China are still increasing. Liver metastasis is the most common metastatic pattern of CRC and a major cause of treatment failure. It has been reported in the literature that 15% to 20% of patients have liver metastases at the time of definitive diagnosis, while another 15% to 40% of CRC patients experience liver metastases after treatment of the primary tumor.

For CRC patients with liver metastases only, local treatment based on liver resection remains the main treatment to obtain long-term survival. The 5-year survival rate after resection is about 30% to 40%, while the 10-year survival rate can reach 20%. Resection of liver metastases as much as possible or combination with radiofrequency treatment and stereotactic body radiation therapy (SBRT) to achieve no evidence of disease (NED) is the main treatment for patients with colorectal liver metastases (CRLM) [1]. However, despite advances in surgical techniques, only 10% to 20% of patients are initially resectable, and most patients with liver metastases are initially unresectable. Finding ways to convert more patients from initially unresectable to surgically resectable is the key to improve the survival of patients with CRLM.

For patients with initially unresectable liver metastases, the initial treatment option is usually systemic chemotherapy. Multiple clinical studies have confirmed that neoadjuvant chemotherapy can shrink and downstage liver metastases in patients with CRLM, so that patients with unresectable liver metastases can obtain the chance of surgical resection. G. Folprecht et al. analyzed several clinical studies that reported the objective response rate (ORR) and initial resection rate of unresectable metastases and found that a strong positive correlation between ORR and resection rate in studies that included CRC patients with liver only metastases (γ = 0.96, P = 0.002). This study concluded that there was a significant positive correlation between the ORR of preoperative chemotherapy and the reoperation rate of liver metastases [2]. Therefore, ORR and R0 resection rate of liver metastases were used as the study’s endpoints to evaluate the efficacy of neoadjuvant chemotherapy regimens for CRLM.

To improve the ORR and resection rate of liver metastases, researchers have tried to use more active treatment regimens, such as FOLFOXIRI triplet regimen consisting of 5-FU/CF, L-OHP, and CPT-11, or combination of targeted drugs with a chemotherapy backbone such as bevacizumab targeting vascular endothelial growth factor (VEGF) and cetuximab targeting epidermal growth factor receptor (EGFR). In the observational phase Ⅳ study First BEAT, among 704 patients with initially unresectable metastatic colorectal cancer (mCRC) whose metastases were confined to the liver, 84 patients (11.9%) underwent curative resection of liver metastases after treatment with bevacizumab plus first-line chemotherapy, of which 69 patients (9.8%) achieved R0 resection [3]. However, the study by Saltz LB et al. analyzed the efficacy of first-line bevacizumab plus FOLFOX/XELOX versus FOLFOX/XELOX regimen alone in 1400 patients with advanced CRC, and the results showed that the combination of bevacizumab did not significantly improve the ORR or tumor regression [4]. The results of two studies of FOLFOXIRI triplet regimen, GONO and HORG, showed that FOLFOXIRI triplet regimen could improve the ORR and R0 resection rate of metastases compared with FOLFIRI doublet regimen. In the GONO study, the overall ORR increased from 41% for the doublet regimen to 66% for triplet regimen; P = 0.0002; the R0 resection rate was 15% vs. 6%, respectively (P = 0.033); in the HORG study, the overall ORR increased from 33.6% for doublet regimen to 43% for triplet regimen, P = 0.168; and the R0 resection rate was 10% vs. 4%, respectively (P = 0.008) [5]. The results of GONO study showed that compared with doublet regimen, FOLFOXIRI triplet regimen could achieve longer median overall survival (OS) (23.4 months vs. 16.7 months, P = 0.026). However, the toxicity and adverse events of triplet chemotherapy were significantly increased compared with the standard doublet regimen, especially hematological toxicity and peripheral neurotoxicity, which reduced the tolerance and compliance of patients [6].

Cetuximab, a drug targeting epidermal growth factor receptor (EGFR), has shown good efficacy in combination with chemotherapy. The OPUS study results showed that cetuximab plus FOLFOX improved the response rate from 37% to 61% compared to FOLFOX alone in KRAS wild-type patients [7]. The CELIM study showed that cetuximab plus standard chemotherapy (FOLFOX6 or FOLFIRI doublet regimen) resulted in a high response rate in the first-line treatment of mCRC patients, with significant tumor shrinkage in 79% of KRAS wild-type patients, surgery in 42% of all patients, and an R0 resection rate of 35% [8, 9]. In a meta-analysis that included 4 RCTs (PRIME, CRYSTAL, OPUS, and COIN) comparing chemotherapy of cetuximab/panitumumab plus doublet regimen (FOLFOX/FOLFIRI/XELOX) with doublet regimen alone (FOLFOX/FOLFIRI/XELOX) for advanced CRC, the response rate increased from 43% to 72% with cetuximab/panitumumab combination treatment (RR 1.67, 95% CI 1.28-2.18; P = 0.0001) [7, 10].

In summary, compared with the traditional doublet (FOLFOX/FOLFIRI/XELOX) treatment, the triplet or doublet treatment plus cetuximab can further improve the ORR of chemotherapy and improve the resection rate of liver metastases. Logically, some researchers propose that triplet regimen plus cetuximab may achieve higher ORR. Assenat et al. conducted a phase Ⅱ single-arm study to investigate the efficacy of cetuximab plus FOLFOXIRI (CPT-11 180 mg/m^2^) in advanced CRC. The results showed that for KRAS wild-type patients, the overall ORR was 83.3%, and PFS and OS were 9.5 and 24.7 months, respectively [11]. The study by Folprecht et al. further explored the safety and the response rate of the combination of cetuximab and triplet regimen in first-line treatment of advanced CRC. The results showed that the ORR of the quadruplet regimen was 75%, with Grade 3/4 adverse events including diarrhea (25%), agranulocytosis (40%), and rash (15%) [12]. In another single-arm study reported by Antoniotti et al. (GONO MACBETH), the ORR of cetuximab plus triplet (CPT-11 130 mg/m^2^) regimen was 71.6%, and the R0 surgical resection rate was 50% in patients with liver metastases only [13]. Geissler et al. further reported a study (VOLFI) of cetuximab plus triplet regimen versus triplet regimen for first-line treatment of advanced CRC. The study results showed that the ORR of the quadruplet regimen of cetuximab plus triplet (CPT-11 150 mg/m^2^) versus triplet regimen were 85.7% and 60.6%, respectively, and the R0 surgical resection rates of metastatic lesions in subgroup analysis were 50% vs. 27.3%, respectively.

Based on the above studies, for patients with CRLM, either triplet regimen or cetuximab plus doublet (FOLFOX/FOLFIRI) regimen or cetuximab plus triplet regimen can achieve a high ORR and thereby improving the resection rate of liver metastases. However, there is no randomized controlled study data to answer whether cetuximab plus triplet regimen can further improve the ORR and surgical resection rate of liver metastases of advanced CRC in the first-line setting compared with cetuximab plus FOLFOX regimen. Therefore, we intend to further carry out a randomized,controlled clinical study of cetuximab plus FOLFOXIRI regimen versus cetuximab plus FOLFOX regimen in the first-line treatment of patients with initially unresectable CRLM, to answer the question that whether cetuximab plus FOLFOXIRI regimen can improve the overall ORR, surgical resection rate and OS compared with cetuximab plus FOLFOX regimen in patients with previously untreated, initially unresectable CRLM patients.

References:

1. Wang Y, Wang ZQ, Wang FH et al. The Role of Adjuvant Chemotherapy for Colorectal Liver Metastasectomy after Pre-Operative Chemotherapy: Is the Treatment Worthwhile? J Cancer 2017; 8: 1179-1186.

2. Folprecht G, Grothey A, Alberts S et al. Neoadjuvant treatment of unresectable colorectal liver metastases: correlation between tumour response and resection rates. Ann Oncol 2005; 16: 1311-1319.

3. Okines A, Puerto OD, Cunningham D et al. Surgery with curative-intent in patients treated with first-line chemotherapy plus bevacizumab for metastatic colorectal cancer First BEAT and the randomised phase-III NO16966 trial. Br J Cancer 2009; 101: 1033-1038.

4. Saltz LB, Clarke S, Diaz-Rubio E et al. Bevacizumab in combination with oxaliplatin-based chemotherapy as first-line therapy in metastatic colorectal cancer: a randomized phase III study. J Clin Oncol 2008; 26: 2013-2019.

5. Souglakos J, Androulakis N, Syrigos K et al. FOLFIRINOX (folinic acid, 5-fluorouracil, oxaliplatin and irinotecan) vs FOLFIRI (folinic acid, 5-fluorouracil and irinotecan) as first-line treatment in metastatic colorectal cancer (MCC): a multicentre randomised phase III trial from the Hellenic Oncology Research Group (HORG). Br J Cancer 2006; 94: 798-805.

6. Fornaro L, Lonardi S, Masi G et al. FOLFIRINOX in combination with panitumumab as first-line treatment in quadruple wild-type (KRAS, NRAS, HRAS, BRAF) metastatic colorectal cancer patients: a phase II trial by the Gruppo Oncologico Nord Ovest (GONO). Ann Oncol 2013; 24: 2062-2067.

7. Tabernero J, Van Cutsem E, Diaz-Rubio E et al. Phase II trial of cetuximab in combination with fluorouracil, leucovorin, and oxaliplatin in the first-line treatment of metastatic colorectal cancer. J Clin Oncol 2007; 25: 5225-5232.

8. Folprecht G, Gruenberger T, Bechstein W et al. Survival of patients with initially unresectable colorectal liver metastases treated with FOLFOX/cetuximab or FOLFIRI/cetuximab in a multidisciplinary concept (CELIM study). Ann Oncol 2014; 25: 1018-1025.
 9. Folprecht G, Gruenberger T, Bechstein WO et al. Tumour response and secondary resectability of colorectal liver metastases following neoadjuvant chemotherapy with cetuximab: the CELIM randomised phase 2 trial. Lancet Oncol 2010; 11: 38-47.

10. Petrelli F, Barni S. Resectability and outcome with anti-EGFR agents in patients with KRAS wild-type colorectal liver-limited metastases: a meta-analysis. Int J Colorectal Dis 2012; 27: 997-1004.

11. Assenat E, Desseigne F, Thezenas S et al. Cetuximab plus FOLFIRINOX (ERBIRINOX) as first-line treatment for unresectable metastatic colorectal cancer: a phase II trial. Oncologist 2011; 16: 1557-1564.

12. Folprecht G, Hamann S, Schutte K et al. Dose escalating study of cetuximab and 5-FU/folinic acid (FA)/oxaliplatin/irinotecan (FOLFIRINOX) in first line therapy of patients with metastatic colorectal cancer. BMC Cancer 2014; 14: 521.

13. Cremolini C, Antoniotti C, Lonardi S et al. Activity and Safety of Cetuximab Plus Modified FOLFIRINOX Followed by Maintenance With Cetuximab or Bevacizumab for RAS and BRAF Wild-type Metastatic Colorectal Cancer: A Randomized Phase 2 Clinical Trial. JAMA Oncol 2018; 4: 529-536.

## 2. Study objectives

### 2.1 Primary objective

To compare the ORR of cetuximab plus FOLFOXIRI regimen with cetuximab plus FOLFOX in patients with initially unresectable CRLM without previous palliative treatment.

### 2.2 Secondary objectives

- Depth of response (DpR)
- R0/resection rate
- Early tumor shrinkage (ETS)
- Progression-free survival (PFS) of the whole group
- OS in the whole group
- Safety (adverse reactions of chemotherapy, surgical complications, etc.)

**3. Study population**

The target population for this study is patients with initially unresectable RAS/BRAF wild-type CRC with liver metastases only, and without previous palliative treatment.

### 3.1 Inclusion criteria

Patients must meet the following study criteria:

- Age ≥ 18 years and ≤ 70 years.
- Histologically confirmed colorectal adenocarcinoma.
- Liver metastasis confirmed by imaging or pathology.
- The multidisciplinary team (MDT) determines that the liver metastases are unresectable, which is specifically defined as ① metastatic lesions ≥ 5; ② ineligible for R0 resection; ③ expected insufficient residual liver volume after resection; ④ unable to preserve all three hepatic veins after resection, unable to ensure that the blood flow and bile ducts of the residual liver into and out of the liver could be preserved, and unable to preserve the adjacent two liver segments. Patients who meet any of the above criteria can be determined as having initially unresectable liver metastases.
- Patients with wild-type RAS (KRAS and NRAS exons 2,3, and 4) and BRAF codon 600.
- No prior treatment for liver metastases, including chemotherapy, surgery, radiotherapy, transcatheter arterial chemoembolization (TACE), and targeted therapy.
- Absence of extrahepatic metastasis confirmed by CT, MRI or PET/CT (if necessary) (enrollment can be considered if there is a lung or lymph node lesion less than 10 mm, which is difficult to determine metastases).
- Normal hematologic function (platelets > 90 × 10^9^/L; leukocytes > 3 × 10^9^/L; neutrophils > 1.5 × 10^9^/L).
- Serum bilirubin ≤ 1.5 times the upper limit of normal (ULN) and transaminases ≤ 5 times ULN.
- No ascites, normal coagulation function, albumin ≥ 35 g/L.
- Liver function: Child-Push score: Class A
- Serum creatinine < ULN, or calculated creatinine clearance > 50 ml/min (using the Cockcroft-Gault formula).
- ECOG score 0-1.
- Life expectancy > 3 months.
- Sign written informed consent.
- Willing and able to be followed up until death or end of study or study termination.

**3.2 Exclusion criteria**

Patients who meet any of the following criteria will be excluded from the study:

- Presence of any extrahepatic metastasis and/or primary tumor that cannot be resected with radical surgery.
- Serious arterial embolism or ascites.
- Have bleeding tendency or coagulation disorder.
- Have hypertensive risk or hypertensive encephalopathy.
- Serious uncontrolled systemic complications such as infection or diabetes.
- Clinically significant cardiovascular disease such as cerebrovascular accident (within 6 months prior to enrollment), myocardial infarction (within 6 months prior to enrollment), uncontrolled hypertension despite appropriate medical treatment. Unstable angina, congestive heart failure (NYHA class 2-4), cardiac arrhythmia requiring medication.
- History or physical evidence of central nervous system disease (e.g., primary brain tumor, epilepsy uncontrolled by standard of care, any history of brain metastases or stroke).
- History of other malignancies (except basal cell carcinoma of the skin and/or carcinoma in situ of the cervix after radical surgery) within the past 5 years.
- Treatment with any ongoing investigational drug within the last 28 days prior to the study.
- Any residual toxicity from prior chemotherapy (except alopecia), such as peripheral neuropathy ≥ NCI CTC v4.03 Grade 2, will not be considered for oxaliplatin-containing regimen.
- Hypersensitivity to any drug in the study.
- Pregnant and lactating women.
- Women of childbearing age (< 2 years after menstruation) or men of childbearing potential who are not using or refuse to use effective non-hormonal contraception (intrauterine contraceptive ring, barrier contraceptives combined with spermicidal gel, or surgical sterilization).
- Unable or unwilling to comply with the study protocol.
- Patients with any other diseases, dysfunction caused by metastatic lesions, or suspected disease found by physical examination, indicating possible contraindications to the use of the investigational drug or putting the patients at high risk of treatment-related complications.

## 4. Estimated sample size/randomization

We expect an ORR of 80% in patients treated with cetuximab plus FOLFOXIRI regimen and 60% in patients treated with cetuximab plus FOLFOX regimen. With a type 1 error probability two-sided α = 10% and a power β = 80%, a total of 128 patients are required to be enrolled into randomization. Considering a dropout rate of 10%, a total of 140 patients are planned to be enrolled in this study, with 70 patients in each group.

**Randomization method:** Cetuximab plus FOLFOXIRI regimen or cetuximab plus FOLFOX regimen will be assigned by the Interactive Web Response System (IWRS) randomization system using a stratified block randomization method. Stratified randomization will be performed with the site of primary intestinal lesion (left- or right-sided) and determinate to the resectability of liver metastases (technically potentially resectable or technically resectable with ≥ 5 metastatic lesions having poor prognosis) as stratification factors at enrollment.

**Randomization concealment and implementation:** Randomization will be implemented in an IWRS. Stratified block randomization protocol is preset in this system. The corresponding app can be downloaded on the mobile phone, and the authorized investigator can input the basic information of the enrolled case to know the randomization result. Each patient has a unique randomization number, which remains unchanged throughout the study. All randomization processes will be recorded in the randomization system.

**5. Study regimen**

### 5.1 Study steps and procedures

1. The screening procedure begins after the patients sign the written informed consent.
2. CT and/or MRI scans (with PET-CT where available) are used to assess liver metastases and to exclude extrahepatic metastases.
3. An MDT meeting is held prior to chemotherapy and the resectability of metastases is assessed and determined based on the given criteria. The liver surgeon performs a comprehensive assessment of each patient’s baseline liver metastases, record in detail the number and size of liver metastases, the liver segment involved, whether the hepatic vein, portal vein, and inferior vena cava are involved, and explain in detail why each patient is considered initially unresectable.
4. Eligible patients at screening are randomly assigned by the study physician to receive either cetuximab plus FOLFOXIRI triplet regimen or cetuximab plus FOLFOX doublet regimen.
5. CT and/or MRI scans are performed every 4 cycles after chemotherapy, with additional examinations if necessary, and the tumor is assessed according to RECIST v1.1 criteria; MDT discussion is held regularly to determine the resectability of patients’ liver metastases. The reason why each patient can be converted to surgically resectable is also specified.
6. In this study, 8 courses (no less than 6 courses) of conversion chemotherapy are recommended, and some patients also need to appropriately prolong the treatment course. This is mainly because that this study includes patients with unresectable colorectal liver metastases, who usually have a large number of liver metastases, large lesions, with high-risk CRS score. Current clinical practice recommends surgical resection after adequate chemotherapy to achieve more benefit.
7. For the treatment of liver metastases, surgical resection with or without intraoperative radiofrequency treatment can be used. For the lesions that cannot be resected or treated with radiofrequency during surgery but are suitable for SBRT treatment, SBRT should be added after surgery. The timing of resection of primary intestinal lesions can be determined by the investigator according to the patient’s condition. For patients with liver metastases converted to surgical resectable, primary intestinal disease can be resected before chemotherapy, or can be resected at the same time or in stages with liver metastases after successful conversion. For patients with rectal cancer, local rectal radiotherapy can be performed when needed, and long-course or short-course radiotherapy can be used.
8. Patients with liver metastases can receive chemotherapy alone with or without cetuximab as adjuvant chemotherapy after surgery. Patients in the cetuximab + mFOLFOXIRI regimen group can receive doublet regimen chemotherapy after surgery. Patients should receive tumor assessment before postoperative adjuvant chemotherapy, including at least CEA, liver MRI scan + enhanced scan, and chest and lower abdomen pelvic CT (if necessary). Patients will receive up to a total of 12 courses of chemotherapy before and after surgery, and treatment will be interrupted in the event of disease progression, intolerable adverse reactions, or patient refusal.
9. For patients who cannot undergo surgery or are unwilling to receive surgery but can receive radiofrequency treatment, radiofrequency treatment can be given. The total number of planned chemotherapy courses treated before and after radiofrequency treatment is 12 courses.
10. For patients who received adequate chemotherapy and whose tumor is controlled (PR or SD achieved) but still cannot undergo resection or radiofrequency, the treatment regimen can be changed for conversion therapy, and once the conversion is successful, surgical treatment and/or radiofrequency therapy can be performed. These patients may also discontinue chemotherapy or enter maintenance chemotherapy.
11. Patients who have disease progression during chemotherapy can discuss with investigator to decide whether to receive further palliative treatment. For patients with unresectable liver metastases after chemotherapy, whether to perform the primary lesion resection can be decided according to the patient’s wishes and specific circumstances.
12. Patients who have completed the treatment specified in the protocol or have not completed the treatment due to intolerable toxicity, but have not experienced tumor progression, are followed up every 3 months, including physical examination and imaging examination until tumor progression. For patients with tumor progression, follow-up will be performed by phone every 3 months until death. The following information should be collected as much as possible during follow-up: subsequent anti-tumor treatment and survival data. Patients with preoperative disease progression should withdraw from the study and enter the follow-up period as well.

Flowchart:

Technically resectable but ≥ 5 metastatic lesions or technically unresectable

Randomization

Cetuximab plus FOLFOXIRI

Cetuximab plus FOLFOX

Chemotherapy: 6 to 8 courses (3 to 4 months)

Resectable

Progression

Disease controlled but remains unresectable

Switch to other therapy or maintenance therapy

Surgical resection

Continue chemotherapy: 4 to 8 courses (2 to 3 months)

Switch to other therapy

Progression

### 5.2 Dosing regimens and treatment courses

**Preoperative conversion chemotherapy regimen:**

Eligible patients enrolled are randomized to receive either triplet regimen of cetuximab plus FOLFOXIRI or doublet regimen of cetuximab plus FOLFOX. The specific regimen is as follows:

**Cetuximab + mFOLFOX6 regimen**

Cetuximab 500 mg/m^2^ ivd 90 min d1

Oxaliplatin 85 mg/m^2^ ivd 3h d1

L-leucovorin 200 mg/m^2^ ivd 2h d1 (d,l-leucovorin 400 mg/m^2^)

5-FU 400 mg/m^2^ iv d1

5-FU 2400 mg/m^2^ ivd civ 46h d1

**Cetuximab + mFOLFOXIRI regimen**

Cetuximab 500 mg/m^2^ ivd 90 min d1

Irinotecan 130 mg/m2 ivd d1 (The starting dose of irinotecan is 130 mg/m2, according to the “3 + 3” design. If 0/3 patients experience dose limiting toxicity (DLT), the dose should be increased to 150 mg/m2; if 1/3 patients experiences DLT, the number of enrolled patients should be increased to 6; if ≤ 1/6 patients experience DLT, the dose should be increased to 150 mg/m2; if ≥ 2/6 patients experience DLT, the dose of irinotecan should be decreased to 100 mg/m2.

At the dose of 150 mg/m2, if 0/3 patients experience DLT, the dose is determined to be 150 mg/m2; if 1/3 patients experiences DLT, the number of enrolled patients should be increased to 6; if ≤ 1/6 patients experience DLT, the dose is determined to be 150 mg/m2; and if ≥ 2/6 experience DLT, the dose is determined to be 130 mg/m2). UGT gene detection is recommended. For patients with UGT*28 7/7, UGT*6 A/A, UGT*28 6/7 and UGT*6 A/G, irinotecan at the dose of 130 mg/m2 is used, no need for escalation)

Oxaliplatin 85 mg/m^2^ ivd 3h d1

L-leucovorin 200 mg/m^2^ ivd d1 (d,l-leucovorin 400 mg/m^2^)

5-FU 2400 mg/m^2^ ivd civ 46h d1

The above regimen is repeated every 14 days.

**Number of conversion chemotherapy courses:**

In this study, 8 (no less than 6) courses of conversion chemotherapy are recommended, and some patients also need to appropriately prolong the treatment course. This is mainly because that this study includes patients with unresectable colorectal liver metastases, who usually have a large number of liver metastases, large lesions, with high-risk CRS score. Current clinical practice recommends surgical resection after adequate chemotherapy to achieve more benefit.

**Postoperative adjuvant chemotherapy regimen and the number of courses:**

Patients should receive tumor assessment before postoperative adjuvant chemotherapy, including at least CEA, liver MRI scan + enhanced scan, and chest and lower abdomen pelvic CT (if necessary).

Patients with liver metastases can receive chemotherapy alone with or without cetuximab after surgery. Patients in the cetuximab + mFOLFOXIRI regimen group can receive doublet regimen chemotherapy after surgery. Patients in both groups can receive FUDR HAI on the basis of chemotherapy.

Patients will receive up to a total of 12 courses of chemotherapy before and after surgery, and treatment will be interrupted in the event of disease progression, intolerable adverse reactions, or patient refusal.

**6. Chemotherapy dose adjustment and concomitant medication**

**6.1 Overview**

Toxicities will be assessed according to NCI CTCAE v4.03 criteria starting from the first treatment cycle and will need to be repeated prior to and at the end of each subsequent treatment cycle. The following are general considerations for dose modifications due to chemotherapy-related adverse reactions:

- Dose reductions or interruptions may not be required if, at the discretion of the investigator, the adverse reaction is unlikely to develop into a serious or life-threatening event and will not result in treatment delay or interruption (e.g., alopecia, taste alteration, etc.). If anemia can resolve well with transfusion, no dose reduction or treatment interruption is required.
- If any Grade 1 adverse reactions occur, treatment may be continued at the starting dose without dose interruption.
- Prior to each new treatment cycle, patients should meet the following criteria:

Normal hematologic function (platelets > 90 × 10^9^/L; leukocytes > 3 × 10^9^/L; neutrophils > 1.5 × 10^9^/L).

Serum bilirubin ≤ 1.5 times ULN and transaminases ≤ 5 times ULN.

Serum creatinine < ULN, or calculated creatinine clearance > 50 ml/min (using the Cockcroft-Gault formula).

Recovery to baseline or ≤ Grade 1 from any treatment-related Grade 3/4 non-hematologic adverse reactions (except alopecia).

- If the patient is ineligible for FOLFOXIRI or FOLFOX chemotherapy, treatment with cetuximab or FU/CF chemotherapy can be continued. If the patient is ineligible for cetuximab treatment, FOLFOXIRI or FOLFOX chemotherapy can be continued.

**6.2 Dose modifications due to hematologic toxicity**

Dose modifications during this or subsequent cycles due to DLT are made as follows:

- For Grade 3 ANC decreased, dose modification of investigational drugs (oxaliplatin/irinotecan, 5-FU) is not needed. For Grade 3 PLT decreased, febrile Grade 3 ANC decreased, or Grade 4 ANC decreased, adjust the dose of oxaliplatin/irinotecan to 80% of the original dose. For Grade 4 PLT decreased or febrile Grade 4 ANC decreased, adjust the dose of oxaliplatin/irinotecan to 70% of the original dose.
- After a dose reduction, if the same or higher grade of toxicity recurs, further reduce the dose of oxaliplatin/irinotecan to 60%. If Grade 4 toxicity persists despite dose modification for Grade 4 toxicity, discontinue oxaliplatin/irinotecan and continue the treatment with cetuximab or FU/CF chemotherapy.
- After the second dose reduction, if the same or higher grade of toxicity recurs, discontinue oxaliplatin/irinotecan and continue the treatment with cetuximab or FU/CF chemotherapy.

**6.3 Dose modifications due to non-hematologic toxicity**

If the non-hematological toxicity is not clearly related to a certain investigational drug, consider adjusting the dose of all drugs that may cause the adverse reactions; if the non-hematological toxicity is clearly related to a certain investigational drug, consider only adjusting the dose of this drug, while the dose of other investigational drugs may not be adjusted.

Dose adjustment for the first occurrence of non-hematological toxicity:

- Diarrhea
- If diarrhea occurs at any time, the patient must recover to less than Grade 1 diarrhea before restarting treatment. For Grade 1 diarrhea, administer the investigational drug at the original dose; for Grade 2 diarrhea, administer irinotecan and 5-FU at 85% of the original dose; after a dose reduction, if the same or higher grade of toxicity recurs, further reduce irinotecan and 5-FU to 75% of the original dose. For Grade 3 diarrhea, administer irinotecan and 5-FU at 75% of the original dose; after dose reduction, if the same or higher grade of toxicity recurs, further reduce irinotecan and 5-FU to 60% of the original dose. If the patient presents with the following conditions, including Grade 4 diarrhea, diarrhea with fever, and diarrhea with Grade 3 to 4 granulocytopenia, administer irinotecan and 5-FU at 60% of the original dose. After dose reduction, discontinue the treatment if the same or higher grade of toxicity recurs.
- Bilirubin increased

Once bilirubin increased occurs, it is recommended to rule out bilirubin increased caused by biliary obstruction and tumor progression. If bilirubin is > 1.5 × ULN, irinotecan is no longer recommended.

- Transaminases increased

In this study, if the transaminases level is abnormal, it is recommended to rule out transaminase increased caused by tumor progression. Drug treatment will be withheld until toxicity recovers to baseline or ≤ Grade 1 before treatment can be restarted. For Grade 1 and 2 transaminases increased, no dose adjustment is made; for Grade 3 transaminase increased, adjust the dose of oxaliplatin/irinotecan to 80% of the original dose. For Grade 4 transaminases increased, discontinue all treatment. Once a dose reduction has been made due to hepatic function, no further dose reduction is recommended if no worsening of indicators is observed.

- Stomatitis

If stomatitis occurs at any time, the patient must recover to less than Grade 1 stomatitis before restarting treatment. Dose reduction is required as follows: for Grade 1 stomatitis, administer the investigational drug at the original dose; for Grade 2 stomatitis, administer 5-FU at 85% of the original dose; for Grade 3 stomatitis, administer 5-FU at 70% of the original dose; for Grade 4 stomatitis, discontinue all treatment.

- Cardiotoxicity

Once angina pectoris or myocardial infarction occurs, discontinue 5-FU treatment permanently.

- Hand-foot syndrome:

For Grade 3 or 4 toxicity, withhold the drug treatment until the toxicity recovers to Grade ≤ 1 before restarting the treatment. Dose reduction is required as follows: for Grade 3 hand-foot syndrome, administer 5-FU at 75% of the original dose; for Grade 4 hand-foot syndrome, administer 5-FU at 60% of the original dose.

- Neurotoxicity

For Grade 3 or 4 neurotoxicity, withhold the drug treatment until the neurotoxicity recovers to Grade ≤ 1 before restarting the treatment. Dose reduction is required as follows: for Grade 2 neurotoxicity, administer oxaliplatin at 85% of the original dose; for patients with Grade 3/4 neurotoxicity, discontinue oxaliplatin treatment and patients in the cetuximab plus FOLFOXIRI group may continue to receive either cetuximab or FU/CF chemotherapy.

**6.4** **Management of adverse reactions due to cetuximab**

- Infusion-related reactions, including anaphylaxis

Severe infusion-related reactions, including anaphylaxis, can occur frequently and in some cases can be fatal. Once severe infusion reactions occur, discontinue the drug immediately and permanently and provide emergency treatment. Some of these reactions may be reported as anaphylaxis, anaphylactoid reactions or presented as cytokine release syndrome (CRS). Symptoms may occur during the first infusion and hours after the end of the infusion or in subsequent instillations. The physician is recommended to inform the patient of the possibility of delayed onset of this reaction and ask the patient to contact the physician immediately if symptoms occur. Possible symptoms include bronchospasm, urticaria, high or low blood pressure, loss of consciousness, or shock. Angina pectoris, myocardial infarction, or sudden cardiac arrest are rare.

Anaphylaxis may occur within minutes of the first infusion, e.g., caused by preformed IgE antibodies that cross-react with the drug. These reactions are usually accompanied by bronchospasm and urticaria. These reactions may occur despite premedication. Patients who are allergic to red meat or tick bites or who are positive for IgE antibodies to cetuximab (α-1-3-galactose) have much higher risk of anaphylaxis. For these patients, the risks and benefits, including alternative therapies, should be carefully assessed before application of the drug, and the drug can only be used under the close supervision of trained professionals equipped with resuscitation equipment.

The first dose should be administered slowly, the instillation rate should not exceed 5 mg/min, and all vital signs should be closely monitored for at least 2 hours. If an infusion-related reaction occurs within 15 minutes of the first dose, the instillation should be discontinued. Careful risk-benefit assessment should be performed before subsequent infusions, including consideration of whether the patient has preformed IgE antibodies.

If infusion-related reactions occur late in the instillation or in the subsequent instillations, the corresponding management depends on the severity of the reaction:

1. Grade 1: perform instillation continuously and slowly under close monitoring
2. Grade 2: perform instillation continuously and slowly and adopt symptomatic treatment immediately
3. Grade 3 and 4: stop the instillation immediately, perform active symptomatic treatment and stop further treatment with the drug.

CRS usually occurs within 1 hour after instillation of the drug, and generally is not accompanied by bronchospasm and urticaria. CRS is usually the most serious adverse reaction of the first instillation of the drug.

Mild and moderate infusion reactions are very common and include symptoms such as fever, chills, dizziness, or dyspnea, and occur primarily during the first infusion. If the patient develops mild to moderate relevant reactions, the instillation rate of the drug should be slowed down. It is recommended to take the adjusted rate during all subsequent instillation processes.

Patients should be closely monitored, especially during the first dose. It is recommended that special attention should be paid to patients with low performance status or concomitant cardiopulmonary disease.

- Skin reactions

The main adverse reaction of the drug is skin reactions. Severe skin reactions nay occur, especially when the drug is combined with chemotherapy. The increased risk of secondary infections, primarily bacterial, can lead to complications such as staphylococcal scalded skin syndrome, necrotizing fasciitis, and sepsis, which in some cases have been reported to be fatal.

Skin reactions are very common and treatment with the drug should be interrupted or discontinued when they occur. Clinical practice guidelines recommend prophylactic treatment with oral tetracycline (6-8 weeks) and 1% hydrocortisone topical cream with moisturizers. Moderate to high potency corticosteroids or oral tetracycline antibiotics have been used in the treatment of skin reactions.

For patients who develop intolerable severe (≥ Grade 3; Common Terminology Criteria for Adverse Events, NCI-CTCAE) skin reactions, treatment with the drug must be interrupted. Treatment should only be restarted when the reactions have resolved to Grade 2 (see National Cancer Institute website for detailed CTCAE grading classifications). If the severe skin reaction occurs for the first time, no dose adjustment of the drug is required.

If the severe skin reaction occurs for the second or third time, the use of the drug must be interrupted again.

Resume treatment at a lower dose only if the reaction has resolved to Grade 2 (for the second occurrence: 200 mg/m^2^ by body surface area; for the third occurrence: 150 mg/m^2^ by body surface area).

If the severe skin reaction occurs for the fourth time, or the skin reactions cannot be resolved to Grade 2 after treatment interruption, the treatment with the drug should be discontinued permanently.

**6.5 Management of other toxicities**

If other Grade < 3 toxicities not listed above occur, give symptomatic treatment whenever possible. If Grade 3 toxicity occurs, withhold the drug treatment. When the toxicity recovers from Grade 3 to Grade ≤ 1, administer the corresponding investigational drug at 85% of the original dose in the next treatment cycle. If Grade 4 toxicity occurs, discontinue all drug treatment. If the toxicity still does not resolve to Grade ≤ 1 four weeks after the start of the next scheduled cycle, the patient should withdraw from the treatment and be followed up according to the protocol until the toxicity is resolved.

However, in the event of certain Grade ≥ 3 adverse events (e.g., when a patient develops symptoms of fatigue, sexual function changes, and clinically insignificant dry skin), the investigator may also decide to allow the patient to continue treatment.

For the second occurrence of non-hematological toxicity, the following dose adjustment methods should be used:

If the patient experiences Grade 3 toxicity again, although the dose of the investigational drug has been previously adjusted, the study dose can still be adjusted again if the investigator believes that the treatment is in the best interest of the patient (otherwise the treatment should be stopped). Adjust the second dose to 70% of the original dose. However, for the second occurrence of Grade 4 toxicity, the above principles do not apply, and the patient should discontinue treatment in this case. If the dose has already been reduced, it cannot be increased thereafter.

**6.6 Concomitant medications:**

- To prevent vomiting in the combination group, intravenous H3 receptor antagonists with or without dexamethasone is routinely used to stop vomiting before each course of chemotherapy, and NK-1 inhibitors (aprepitant) can also be given.
- To prevent the occurrence of anaphylaxis, diphenhydramine 20 mg is routinely administered intramuscularly 30 minutes before cetuximab chemotherapy.
- The investigator may give G-CSF support according to the patient’s bone marrow adverse reactions. However, G-CSF is not recommended for the first prophylactic treatment.

Other concomitant medications may be determined by the investigator on a patient-by-patient basis.

**7. Efficacy evaluation**

**7.1 Indicators of effectiveness evaluation**

All enrolled patients (intent-to-treat population) will be analyzed for efficacy. The indicators of efficacy evaluation of this study include:

- ORR (partial response (PR) plus complete response (CR)): assessed by the investigator using RECIST v1.1 criteria.
- DpR: the investigator assesses DpR by measuring the ratio of maximum tumor regression to baseline tumor, and calculates the median value.
- R0 resection rate: defined as the proportion of patients who achieve complete resection (pathologically defined R0 resection) after treatment with cetuximab plus FOLFOXIRI regimen or cetuximab plus FOLFOX regimen according to the study protocol.
- ETS: target lesion reduction of a least 20% from the nadir following 4 treatment courses assessed using the RECIST version 1.1 criteria
- PFS: assessed by the investigator using RECIST v1.1, defined as the time from the start of study treatment to disease progression, or relapse after resection of liver metastases, or death due to any cause.
- OS: defined as the time from the start of study treatment to death due to any cause.

**7.2 Indicators of safety evaluation**

The indicators of safety evaluation of this study include:

- Incidence, severity and serious adverse events of chemotherapy-related adverse events, refer to NCI CTCAE v4.03 criteria;
- Incidence and severity of operation-related complications (perioperative mortality, intraoperative bleeding/transfusion, postoperative blood transfusion and number of blood units used), incidence and severity of systemic complications and local liver complications. Evaluation is completed at 48 hours, 1 month, and 3 months postoperatively using the assessment criteria of Clavien-Dindo grading scale system.

**8. Screening and baseline visit**

Patients should not undergo any screening procedures before signing the written informed consent. If any of the following procedures (such as tumor assessment) are completed as a routine clinical practice within the protocol-required time window before patients sign the written informed consent, they are not repeated if the investigator considers the results to be reasonable and acceptable. Only the data of eligible patients screened according to the inclusion and exclusion criteria can be filled in the case report form (CRF). All patients who sign the written informed consent form will be screened, and baseline screening will be performed from 28 days prior to initiation of study treatment to 24 hours prior to initiation of study treatment, including the following:

**Day -28 to -1**

- Signing of written informed consent form: date.
- Demographic characteristics: age, sex.
- Medical history: including gastrointestinal diseases, hypertension, thromboembolic diseases, cardiovascular diseases, renal diseases and other significant diseases.
- Cancer and treatment history: time of first treatment; initial diagnosis of CRC (date, lymph node status, TNM stage, histological tumor grade, primary tumor lesion [colon or rectum]); diagnosis of metastatic disease (date, metastases, CT scan [abdomen, chest, pelvis]); previous treatments (surgical resection of primary tumor, local radiotherapy); previous chemotherapy (regimen, type [adjuvant, neoadjuvant], time, course, dose).
- Concomitant medication: drugs possibly related to the occurrence of adverse events or related to the management of adverse events should be recorded.
- Physical examination: complete physical examination should include head, eyes, ears, nose, throat, cardiovascular, skin, muscles and bones, respiratory, digestive, genitourinary and nervous system; abnormalities observed at baseline should be recorded in the corresponding parts of the general medical history and baseline conditions in the CRF; at subsequent visits, local, symptom-oriented physical examination should be performed; abnormal signs with changes from baseline should be recorded in the patient’s notes; new or worsened abnormalities should be recorded as adverse events in the relevant parts of the adverse events in the CRF.
- Height and weight
- Vital signs: including measurements of pulse rate, sitting systolic/diastolic blood pressure, and body temperature.
- ECOG performance status score.
- ECG.
- Whole body CT scan + enhanced scan.
- FDG-PET: only perform when necessary; not mandatory.
- Liver MRI scan + enhanced scan, contrast agent enhancement with Primovist if available.
- Pelvic MRI scan + enhanced scan is recommended for patients with rectal cancer.
- Tumor assessment: assessed by the investigator based on RECIST 1.1 criteria.
- RAS and BRAF gene status (detection method not limited)

**Day -7 to -1**

- Laboratory tests (dates, values): hematology, biochemistry (including ALP), tumor markers
- Surgery assessment by MDT: resectability is determined based on the following given criteria for unresectability:
- Metastatic lesions ≥ 5
- Ineligible for R0 resection
- Expected insufficient residual liver volume after resection
- Unable to preserve all three hepatic veins after resection, unable to ensure that the blood flow and bile ducts of the residual liver into and out of the liver could be preserved, and unable to preserve the adjacent two liver segments.

Patients who meet any of the above criteria can be determined as having initially unresectable liver metastases.

## 9. Assessment during the study

## 9.1 Pre-chemotherapy assessment

The following assessments will be performed during each course:

- Concomitant medication
- Clinical data: weight, physical examination, vital signs, ECOG performance score
- Laboratory tests: hematology, biochemistry (including ALP) and tumor markers on Day 0 and Day 14
- EKG
- Tumor assessment:

Assessments will be performed every 4 courses and the number of examinations may be increased if necessary.

Tumor measurements/assessments are performed using CT or MRI scans based on RECIST v1.1 criteria.

For each patient, the same assessment method should be used throughout the study, and the assessment should be performed by the same assessor as far as possible.

All metastases detected at baseline screening should be assessed at every tumor assessment.

- MDT assessment:

Every 4 courses, the number of assessments may be increased if necessary.

In this study, 8 (no less than 6) courses of conversion chemotherapy are recommended, and some patients also need to appropriately prolong the treatment course before hepatectomy with or without radiofrequency treatment is considered.

This is mainly because that this study includes patients with unresectable colorectal liver metastases, who usually have a large number of liver metastases, large lesions, with high-risk CRS score. Current clinical practice recommends surgical resection after adequate chemotherapy to achieve more benefit.

- Chemotherapy related adverse events
- Survival

**9.2 Surgery and related assessments**

Surgery data is recorded in the designated section of the CRF form and include surgeon name, surgery type, duration, ischemic time, type of blood product transfused, and hospitalization time.

Surgical complications are assessed at 48 hours, 1 month, and 3 months after surgery. Assessment indicators include perioperative mortality (within 60 days after surgery), intraoperative bleeding/transfusion, postoperative transfusion, systemic complications, and local liver complications. Systemic complications are defined as: pulmonary complications, cardiovascular complications, urethral complications, infections, wound healing, and iatrogenic complications. Local liver complications include bile leakage, bleeding, infectious perihepatic fluid collection, and liver dysfunction. The assessment criteria of Clavien-Dindo grading scale system is used. Resected metastases are examined pathologically to determine if they are completely resected.

**9.3 Post-treatment follow-up**

Patients who have completed the treatment specified in the protocol or have not completed the treatment due to intolerable adverse reactions, but have not experienced tumor progression, are followed up every 3 months, including physical examination and imaging examination until tumor progression. For patients with tumor progression, follow-up is performed by phone every 3 months until death. The following information should be collected as much as possible during follow-up: subsequent anti-tumor treatment and survival data. Patients with preoperative disease progression should withdraw from the study and enter into the follow-up stage as well.

**9.4 Discontinuation and withdrawal criteria**

The investigator has the right to discontinue the investigational drug treatment for a patient or withdraw a patient from the study at any time. In addition, patients have the right to request to discontinue the investigational drug or withdraw from the study at any time and for any reason. Reasons for discontinuation of the investigational drug or study withdrawal may include, but are not limited to, the following:

- Patients withdraw informed consent at any time.
- Presence of a medical event that, at the discretion of the investigator, can jeopardize the patient’s safety if the study is to continue.
- Early withdrawal from the study is considered by the investigator to be in the best interest of the patient.
- Patients do not follow the study protocol.

Every effort should be made to obtain information on patients withdrawn from the study. The primary reason for withdrawal from the study should be recorded on the CRF. After withdrawal of informed consent, patients should not be followed up for any reason. Patients who withdraw from the study should not be replaced by other patients.

**10. Statistical analysis**

**10.1 Study population**

The analysis population of this study includes full analysis set (FAS), per protocol set (PPS) and safety set (SS). The principle of **intention-to-treat (ITT) population** refers to the principle that the analysis is performed according to the original randomized intention, that is, the analysis should include all randomized subjects, and the subjects assigned to any treatment group should be followed up, evaluated and analyzed as members of this group, regardless of the actual treatment received.

**FAS** is defined as the set of data obtained after the least and fairest elimination of data from all subjects, which is as close as possible to the ideal according to the ITT principle.

**PPS** is a subset of FAS whose subjects are sufficiently compliant with the protocol and can be assessed for the effect of treatment and whose characteristics meet the following principles: (1) completion of predetermined treatment; (2) measurability of primary study endpoints; (3) absence of major protocol violations, including violations of the eligibility criteria. If the PP population reaches more than 95% of ITT population, the analysis of PP population is not necessary.

**SS** includes all patients who participate and receive at least one dose of the investigational drug, and if the participating population is not consistent with the as-treated population, the safety analysis population will be based on the as-treated population.

**10.2 Statistical analysis methods**

Categorical data is summarized descriptively using the statistics including frequency (n) and percentage (%), and the 95% confidence interval (CI) of the overall percentage is given. Continuous data is summarized descriptively using statistics including mean, standard deviation (SD), median, minimum and maximum. For time-to-event data, Kaplan-Meier method is used to estimate the survival function, plot the survival curve and estimate the survival time and its 95% CI. The Cochran-Mantel-Haenszel (CMH) method considering the randomization stratification factors is used to perform the hypothesis test for the comparison of response rate between the two groups, and the rate and its 95% CI (Clopper-Pearson method) is estimated for each group. The survival analysis indicators are compared using the stratified log-rank test considering the random stratification factors to compare whether the survival distribution is the same between the two treatments.

**10.3 Analysis plan**

Two data analyses are planned for this study. The first analysis is planned after the last patient completes the study treatment, and the primary analysis parameter is the primary endpoint, ORR. The second analysis is a complete analysis and is performed when a median follow up period of 5 years is reached.

**10.4 Stratified analyses**

The following pre-specified subgroup analyses are planned based on baseline characteristics.

Primary tumor site (left-/right-sided).

Surgical resection of liver metastases (clearly technically unresectable/technically resectable but ≥ 5 metastatic lesions with poor prognosis).

## 11. Adverse events reporting and recording

### 11.1 Definition

Adverse event (AE): The term of adverse event covers the occurrence or worsening of any sign, symptom, syndrome, or disease that occurs in a subject during observation in a clinical study and that affects the health of the subject. This term also includes clinically relevant conditions found in the laboratory or other diagnostic process, such as unplanned diagnosis and treatment; or leading to withdrawal from the trial. An AE may be: a new disease, the worsening of treatment status, symptom or sign, or the worsening of a concomitant disease; the effect of a control drug; a combination of one or more factors unrelated to trial participation. Therefore, the term “AE” does not imply a causal relationship to the investigational drug.

Any untoward medical occurrence that occurs outside of the patient follow-up period specified in this protocol is not an AE.

Some pre-existing conditions, symptoms (such as cancer or other diseases) or medically significant abnormalities confirmed by laboratory tests or instruments (such as ECG) should not be considered as AEs. However, the occurrence of new symptoms as well as the worsening of pre-existing conditions should be considered as AEs. In addition, new abnormalities identified by laboratory or instrumental examination that have a clinical impact on a patient, result in a reduction in the dose of the investigational drug, delay in treatment, or discontinuation of treatment or result in a serious adverse event (SAE) as defined below should be considered as AEs.

SAE: An adverse event that occurs at any dose of the investigational drug or at any time during the observation period, which is as follows:

- 1. Results in death;
  2. Immediately life-threatening, i.e., at the discretion of the investigator, places the patient at risk of death at the time of the event (it does not include an event which hypothetically might have caused death if it is more severe);
  3. Requires inpatient hospitalization or prolongation of existing hospitalization;
  4. Disability refers to an event that can lead to permanent or serious disability/disability of a patient based on clinical determination; disability refers to the significant loss of abilities to carry out activities of daily life;
  5. Results in congenital anomaly (due to the use of medicinal products before fertilization or during pregnancy, resulting the newborn defective;
  6. Medically significant (refers to an event that is not immediately life-threatening or dose not result in death or requires hospitalization, but may jeopardize the patient or may require measures to prevent one of the consequences defined above); requires medical treatment to prevent permanent loss or damage. Examples of such events include: allergic bronchospasm requiring rescue in an emergency room or at home, blood dyscrasias or convulsions that do not require hospitalization.

### 11.2 Scope of expected adverse events

Common adverse reactions:

- Digestive system: diarrhea, nausea, vomiting, abdominal pain, stomatitis, etc. Serious (Grade 3-4) gastrointestinal reactions are relatively rare.
- Blood: neutropenia, anemia, thrombocytopenia.
- Nervous system: headache, paresthesia, vertigo, insomnia, somnolence, etc.
- Skin: rash, exfoliation, etc.
- Others: alopecia, mucositis, pyrexia, weakness, fatigue, hypertension, thrombosis, liver and kidney function damage.

### 11.3 Relevant indicators for determining the causal relationship between adverse events and drugs

Relevant indicators for determining the causal relationship between AEs and drugs: ① Whether there is a reasonable sequential relationship between the time of starting medication and the time of occurrence of suspected adverse reactions. ② Whether the suspected adverse reaction conforms to the known type of adverse reaction of the drug. ③ Whether the suspected adverse reactions can be explained by the patient’s pathological condition, concomitant medication, concurrent therapy and previous therapy. ④ Whether the suspected adverse reactions can be alleviated and resolved after drug discontinuation or dose reduction. ⑤ Whether the same reaction occurs again after re-exposure to the same drug.

Determination of causal relationship between AEs and drug: according to the above five indicators, the causal relationship is determined as the following 5 categories, definite, probable, possible, unlikely and unrelated.

| 1. Definite | The event follows a reasonable temporal sequence from the time of drug administration.  The event resolves after treatment interruption.  AEs been confirmed to reappear after repeated medication (rechallenge). |
| --- | --- |
| 2. Probable | The event follows a reasonable temporal sequence from the time of drug administration.  The event resolves after treatment interruption.  The event cannot be reasonably explained by:  A known symptom of the patient’s clinical condition, or an event caused by other drugs/treatments. |
| 3. Possible | The event follows a reasonable temporal sequence from the time of drug administration.  The event is consistent with the known response to the investigational drug, but the event may also be caused by:  Patient’s clinical condition or other drugs/treatments. |
| 4. Unlikely | The event does not follow a reasonable temporal sequence from the time of drug administration.  The event may have been caused by:  A documented pre-existing condition, or concomitant therapy, or the patient’s clinical condition. |
| 5. Unrelated | The event must have been caused by:  Patient’s clinical condition, or other drugs/treatments. |

### 11.4 Management of adverse events

**11.4.1 Recording of adverse events**

The investigator should explain to the patients and ask them to truthfully reflect the changes in their conditions after medication. Physicians should avoid asking leading questions. Any AEs occurring in the subjects during the trial, including laboratory abnormalities, must be carefully inquired and followed up. All AEs must be determined for its nature, severity and correlation with the drug. The investigator should follow up, observe and record the outcome of all AEs and follow up the patients who withdraw from the trial due to AEs until the AE is resolved. For each event, the following information should be recorded in the AE section of the CRF:

- Severity: Whether an AE is classified as SAE or non-SAE.
- AE: Whenever possible, the specific diagnosis of the event should be recorded. If a diagnosis is not possible, each sign or symptom should be recorded separately, e.g. nausea and vomiting are recorded as two AEs. If an event occurs several times and there is an appropriate time interval to explain that a subsequent condition can be considered a recurring symptom, each AE should be recorded separately on the CRF.
- Start date: The date the event started is recorded. If an abnormal laboratory change is reported as an AE, the date of collection of the first laboratory sample indicating a change in condition is recorded as the start date of the event. The start date of an SAE is defined as the date on which the event begins to meet the criteria for an SAE.
- End date: The date the event ended is recorded. If an abnormal laboratory change is reported as an AE, the collection date of the first post-hoc sample (for which detection can indicate that the condition returns to the pre-event status or returns to normal) is recorded as the end date of the event.
- Relationship to the investigational product
- Therapeutic Intervention: Any therapeutic intervention that is necessary is recorded in the concomitant therapy section of the CRF and noted as drug therapy or non-drug therapy.
- Outcome:

(1) Resolution of the event without sequelae - The patient recovers from the AE without any sequelae observed.

(2) Resolution of the event with sequelae - The patient has recovered from the AE but any sequelae is observed (specify sequelae in the AE section of the CRF).

(3) The event does not resolve - the event persists. (Patients should continue to be followed until definitive final results are available. When follow-up data is collected, follow-up information is recorded in the AE section of the CRF. If the event is serious, a follow-up SAE report needs to be completed.)

(4) Death - The patient dies of an AE. (List the main reason for the patient’s death in the AE section of the CRF, and attach an autopsy report to the CRF, if feasible)

- - Action taken related to the investigational drug:

(1) None - No action is taken related to the investigational drug.

(2) Interruption/adjustment - Withhold the investigational drug and resume when the patient’s symptoms are relieved.

(3) Discontinuation - Permanently discontinue use of the investigational drug.

**11.4.2 Report of serious adverse events**

In case of any SAEs during the trial, in addition to active treatment or rescue, the institute undertaking the clinical study should acknowledge by telephone/fax within 24 hours, fill in the “Serious Adverse Event Report Form” and report to the local provincial drug regulatory authorities, National Medical Products Administration, responsible person of the clinical study and Ethics Committee in written form. The sponsor and its entrusting party, CRO, should immediately notify each participating hospital and ensure that the reporting procedures meet all requirements of laws and regulations.

The contact information of SAE report is as follows:

| Institute | Contact person | Telephone |
| --- | --- | --- |
| Sun Yat-sen University Cancer Center | Yuhong Li | 13602755671 |
| Sun Yat-sen University Cancer Center  Drug Clinical Trial Ethics Committee | Zhongyu Yuan | (020) 87343565 |
| National Medical Products Administration | Division of Drug Research Regulation, Department of Drug Safety Regulation | (010) 68313344-1013 |

In the event of a death case, the investigator should write a comprehensive death case description on the SAE form and keep a SAE report with the CRF on site. If an autopsy is performed, the investigator should proactively obtain a copy of the autopsy report and retain a copy of the autopsy report with the CRF on site.

## APPENDIX I Karnofsky Performance Status

| **Percent** | **Performance Status** |
| --- | --- |
| 100  90  80  70  60  50  40  30  20  10  0 | Normal no complaints; no evidence of disease  Able to carry on normal activity; minor signs or symptoms of disease  Normal activity with effort; some signs or symptoms of disease  Cares for self; unable to carry on normal activity or to do active work  Requires occasional assistance, but is able to care for most of personal needs  Requires considerable assistance and frequent medical care  Disabled; requires special care and assistance  Severely disabled; hospital admission is indicated although death not imminent  Very sick; hospital admission necessary; active supportive treatment necessary  Moribund; fatal processes progressing rapidly  Deceased |

## APPENDIX II Child-Pugh Classification

| Factor | 1 point | 2 point | 3 point |
| --- | --- | --- | --- |
| Encephalopathy（grade） | None | 1 or 2 | 3 or 4 |
| [Ascites](https://baike.baidu.com/item/%E8%85%B9%E6%B0%B4) | Absent | Slight | Moderate |
| Total bilirubin（umol/L） | <34 | 34－51 | >51 |
| Serum albumin（g/L） | >35 | 28-35 | <28 |
| Prothrombin time（seconds＞control） | <4 | 4-6 | >6 |

Class A：total points（5－6）；Class B：total points（7－9） ；Class C：total points≥10

## APPENDIX III Common Terminology Criteria for Adverse Events（ CTCEA V4.03,2010-06-14）partial content

| **Metabolism and nutrition disorders** | | | | | |
| --- | --- | --- | --- | --- | --- |
|  | **Grade** | | | | |
| **Adverse Event** | **1** | **2** | **3** | **4** | **5** |
| Acidosis | pH <normal, but >=7.3 | - | pH <7.3 | Life-threatening consequences | Death |
| Definition: A disorder characterized by abnormally high acidity (high hydrogen-ion concentration) of the blood and other body tissues. | | | | | |
| Alcohol intolerance | - | Present | Severe symptoms; limiting self care ADL | Life-threatening consequences; urgent intervention indicated | Death |
| Definition: A disorder characterized by an increase in sensitivity to the adverse effects of alcohol, which can include nasal congestion, skin flushes, heart dysrhythmias, nausea, vomiting, indigestion and headaches. | | | | | |
| Alkalosis | pH >normal, but <=7.5 | - | pH >7.5 | Life-threatening consequences | Death |
| Definition: A disorder characterized by abnormally high alkalinity (low hydrogen-ion concentration) of the blood and other body tissues. | | | | | |
| Anorexia | Loss of appetite without alteration in eating habits | Oral intake altered without significant weight loss or malnutrition; oral nutritional supplements indicated | Associated with significant weight loss or malnutrition (e.g., inadequate oral caloric and/or fluid intake); tube feeding or TPN indicated | Life-threatening consequences; urgent intervention indicated | Death |
| Definition: A disorder characterized by a loss of appetite. | | | | | |
| Dehydration | Increased oral fluids indicated; dry mucous membranes; diminished skin turgor | IV fluids indicated <24 hrs | IV fluids or hospitalization indicated | Life-threatening consequences; urgent intervention indicated | Death |
| Definition: A disorder characterized by excessive loss of water from the body. It is usually caused by severe diarrhea, vomiting or diaphoresis. | | | | | |

| **Metabolism and nutrition disorders** | | | | | |
| --- | --- | --- | --- | --- | --- |
|  | **Grade** | | | | |
| **Adverse Event** | **1** | **2** | **3** | **4** | **5** |
| Glucose intolerance | Asymptomatic; clinical or diagnostic observations only; intervention not indicated | Symptomatic; dietary modification or oral agent indicated | Severe symptoms; insulin indicated | Life-threatening consequences; urgent intervention indicated | Death |
| Definition: A disorder characterized by an inability to properly metabolize glucose. | | | | | |
| Hypercalcemia | Corrected serum calcium of  >ULN - 11.5 mg/dL; >ULN -  2.9 mmol/L; Ionized calcium  >ULN - 1.5 mmol/L | Corrected serum calcium of  >11.5 - 12.5 mg/dL; >2.9 - 3.1  mmol/L; Ionized calcium >1.5  - 1.6 mmol/L; symptomatic | Corrected serum calcium of  >12.5 - 13.5 mg/dL; >3.1 - 3.4  mmol/L; Ionized calcium >1.6  - 1.8 mmol/L; hospitalization indicated | Corrected serum calcium of  >13.5 mg/dL; >3.4 mmol/L; Ionized calcium >1.8 mmol/L; life-threatening consequences | Death |
| Definition: A disorder characterized by laboratory test results that indicate an elevation in the concentration of calcium (corrected for albumin) in blood. | | | | | |
| Hyperglycemia | Fasting glucose value >ULN - 160 mg/dL; Fasting glucose value >ULN - 8.9 mmol/L | Fasting glucose value >160 - 250 mg/dL; Fasting glucose value >8.9 - 13.9 mmol/L | >250 - 500 mg/dL; >13.9 -  27.8 mmol/L; hospitalization indicated | >500 mg/dL; >27.8 mmol/L; life-threatening consequences | Death |
| Definition: A disorder characterized by laboratory test results that indicate an elevation in the concentration of blood sugar. It is usually an indication of diabetes mellitus or glucose intolerance. | | | | | |
| Hyperkalemia | >ULN - 5.5 mmol/L | >5.5 - 6.0 mmol/L | >6.0 - 7.0 mmol/L; hospitalization indicated | >7.0 mmol/L; life-threatening consequences | Death |
| Definition: A disorder characterized by laboratory test results that indicate an elevation in the concentration of potassium in the blood; associated with kidney failure or sometimes with the use of diuretic drugs. | | | | | |
| Hypermagnesemia | >ULN - 3.0 mg/dL; >ULN -  1.23 mmol/L | - | >3.0 - 8.0 mg/dL; >1.23 - 3.30  mmol/L | >8.0 mg/dL; >3.30 mmol/L; life-threatening consequences | Death |
| Definition: A disorder characterized by laboratory test results that indicate an elevation in the concentration of magnesium in the blood. | | | | | |

| **Metabolism and nutrition disorders** | | | | | |
| --- | --- | --- | --- | --- | --- |
|  | **Grade** | | | | |
| **Adverse Event** | **1** | **2** | **3** | **4** | **5** |
| Hypernatremia | >ULN - 150 mmol/L | >150 - 155 mmol/L | >155 - 160 mmol/L; hospitalization indicated | >160 mmol/L; life-threatening consequences | Death |
| Definition: A disorder characterized by laboratory test results that indicate an elevation in the concentration of sodium in the blood. | | | | | |
| Hypertriglyceridemia | 150 mg/dL - 300 mg/dL; 1.71 mmol/L - 3.42 mmol/L | >300 mg/dL - 500 mg/dL;  >3.42 mmol/L - 5.7 mmol/L | >500 mg/dL - 1000 mg/dL;  >5.7 mmol/L - 11.4 mmol/L | >1000 mg/dL; >11.4 mmol/L; life-threatening consequences | Death |
| Definition: A disorder characterized by laboratory test results that indicate an elevation in the concentration of triglyceride concentration in the blood. | | | | | |
| Hyperuricemia | >ULN - 10 mg/dL (0.59  mmol/L) without physiologic consequences | - | >ULN - 10 mg/dL (0.59  mmol/L) with physiologic consequences | >10 mg/dL; >0.59 mmol/L;  life-threatening consequences | Death |
| Definition: A disorder characterized by laboratory test results that indicate an elevation in the concentration of uric acid. | | | | | |
| Hypoalbuminemia | <LLN - 3 g/dL; <LLN - 30 g/L | <3 - 2 g/dL; <30 - 20 g/L | <2 g/dL; <20 g/L | Life-threatening consequences; urgent intervention indicated | Death |
| Definition: A disorder characterized by laboratory test results that indicate a low concentration of albumin in the blood. | | | | | |
| Hypocalcemia | Corrected serum calcium of  <LLN - 8.0 mg/dL; <LLN - 2.0  mmol/L; Ionized calcium  <LLN - 1.0 mmol/L | Corrected serum calcium of  <8.0 - 7.0 mg/dL; <2.0 - 1.75  mmol/L; Ionized calcium <1.0  - 0.9 mmol/L; symptomatic | Corrected serum calcium of  <7.0 - 6.0 mg/dL; <1.75 - 1.5  mmol/L; Ionized calcium <0.9  - 0.8 mmol/L; hospitalization indicated | Corrected serum calcium of  <6.0 mg/dL; <1.5 mmol/L; Ionized calcium <0.8 mmol/L; life-threatening consequences | Death |
| Definition: A disorder characterized by laboratory test results that indicate a low concentration of calcium (corrected for albumin) in the blood. | | | | | |

| **Metabolism and nutrition disorders** | | | | | |
| --- | --- | --- | --- | --- | --- |
|  | **Grade** | | | | |
| **Adverse Event** | **1** | **2** | **3** | **4** | **5** |
| Hypoglycemia | <LLN - 55 mg/dL; <LLN - 3.0  mmol/L | <55 - 40 mg/dL; <3.0 - 2.2  mmol/L | <40 - 30 mg/dL; <2.2 - 1.7  mmol/L | <30 mg/dL; <1.7 mmol/L; life- threatening consequences; seizures | Death |
| Definition: A disorder characterized by laboratory test results that indicate a low concentration of glucose in the blood. | | | | | |
| Hypokalemia | <LLN - 3.0 mmol/L | <LLN - 3.0 mmol/L; symptomatic; intervention indicated | <3.0 - 2.5 mmol/L; hospitalization indicated | <2.5 mmol/L; life-threatening consequences | Death |
| Definition: A disorder characterized by laboratory test results that indicate a low concentration of potassium in the blood. | | | | | |
| Hypomagnesemia | <LLN - 1.2 mg/dL; <LLN - 0.5  mmol/L | <1.2 - 0.9 mg/dL; <0.5 - 0.4  mmol/L | <0.9 - 0.7 mg/dL; <0.4 - 0.3  mmol/L | <0.7 mg/dL; <0.3 mmol/L; life- threatening consequences | Death |
| Definition: A disorder characterized by laboratory test results that indicate a low concentration of magnesium in the blood. | | | | | |
| Hyponatremia | <LLN - 130 mmol/L | - | <130 - 120 mmol/L | <120 mmol/L; life-threatening consequences | Death |
| Definition: A disorder characterized by laboratory test results that indicate a low concentration of sodium in the blood. | | | | | |
| Hypophosphatemia | <LLN - 2.5 mg/dL; <LLN - 0.8  mmol/L | <2.5 - 2.0 mg/dL; <0.8 - 0.6  mmol/L | <2.0 - 1.0 mg/dL; <0.6 - 0.3  mmol/L | <1.0 mg/dL; <0.3 mmol/L; life- threatening consequences | Death |
| Definition: A disorder characterized by laboratory test results that indicate a low concentration of phosphates in the blood. | | | | | |
| Iron overload | - | Moderate symptoms; intervention not indicated | Severe symptoms; intervention indicated | Life-threatening consequences; urgent intervention indicated | Death |
| Definition: A disorder characterized by accumulation of iron in the tissues. | | | | | |
| Obesity | - | BMI 25 - 29.9 kg/m2 | BMI 30 - 39.9 kg/m2 | BMI >=40 kg/m2 | - |

| **Metabolism and nutrition disorders** | | | | | |
| --- | --- | --- | --- | --- | --- |
|  | **Grade** | | | | |
| **Adverse Event** | **1** | **2** | **3** | **4** | **5** |
| Definition: A disorder characterized by having a high amount of body fat. | | | | | |
| Tumor lysis syndrome | - | - | Present | Life-threatening consequences; urgent intervention indicated | Death |
| Definition: A disorder characterized by metabolic abnormalities that result from a spontaneous or therapy-related cytolysis of tumor cells. | | | | | |
| Metabolism and nutrition disorders - Other, specify | Asymptomatic or mild symptoms; clinical or diagnostic observations only; intervention not indicated | Moderate; minimal, local or noninvasive intervention indicated; limiting age- appropriate instrumental ADL | Severe or medically significant but not immediately life- threatening; hospitalization or prolongation of existing hospitalization indicated; disabling; limiting self care ADL | Life-threatening consequences; urgent intervention indicated | Death |

| **Investigations** | | | | | |
| --- | --- | --- | --- | --- | --- |
|  | **Grade** | | | | |
| **Adverse Event** | **1** | **2** | **3** | **4** | **5** |
| Activated partial thromboplastin time prolonged | >ULN - 1.5 x ULN | >1.5 - 2.5 x ULN | >2.5 x ULN; hemorrhage | - | - |
| Definition: An abnormal laboratory test result in which the partial thromboplastin time is found to be greater than the control value. As a possible indicator of coagulopathy, a prolonged partial thromboplastin time (PTT) may occur in a variety of diseases and disorders, both primary and related to treatment. | | | | | |
| Alanine aminotransferase increased | >ULN - 3.0 x ULN | >3.0 - 5.0 x ULN | >5.0 - 20.0 x ULN | >20.0 x ULN | - |
| Definition: A finding based on laboratory test results that indicate an increase in the level of alanine aminotransferase (ALT or SGPT) in the blood specimen. | | | | | |
| Alkaline phosphatase increased | >ULN - 2.5 x ULN | >2.5 - 5.0 x ULN | >5.0 - 20.0 x ULN | >20.0 x ULN | - |
| Definition: A finding based on laboratory test results that indicate an increase in the level of alkaline phosphatase in a blood specimen. | | | | | |
| Aspartate aminotransferase increased | >ULN - 3.0 x ULN | >3.0 - 5.0 x ULN | >5.0 - 20.0 x ULN | >20.0 x ULN | - |
| Definition: A finding based on laboratory test results that indicate an increase in the level of aspartate aminotransferase (AST or SGOT) in a blood specimen. | | | | | |
| Blood antidiuretic hormone abnormal | Asymptomatic; clinical or diagnostic observations only; intervention not indicated | Symptomatic; medical intervention indicated | Hospitalization indicated | - | - |
| Definition: A finding based on laboratory test results that indicate abnormal levels of antidiuretic hormone in the blood specimen. | | | | | |
| Blood bilirubin increased | >ULN - 1.5 x ULN | >1.5 - 3.0 x ULN | >3.0 - 10.0 x ULN | >10.0 x ULN | - |
| Definition: A finding based on laboratory test results that indicate an abnormally high level of bilirubin in the blood. Excess bilirubin is associated with jaundice. | | | | | |
| Blood corticotrophin decreased | Asymptomatic; clinical or diagnostic observations only; intervention not indicated | Symptomatic; medical intervention indicated | Hospitalization indicated | - | - |

| **Investigations** | | | | | |
| --- | --- | --- | --- | --- | --- |
|  | **Grade** | | | | |
| **Adverse Event** | **1** | **2** | **3** | **4** | **5** |
| Definition: A finding based on laboratory test results that indicate an decrease in levels of corticotrophin in a blood specimen. | | | | | |
| Blood gonadotrophin abnormal | Asymptomatic; clinical or diagnostic observations only; intervention not indicated | Symptomatic; medical intervention indicated; limiting instrumental ADL | Severe symptoms; limiting self care ADL | - | - |
| Definition: A finding based on laboratory test results that indicate abnormal levels of gonadotrophin hormone in a blood specimen. | | | | | |
| Blood prolactin abnormal | Asymptomatic; clinical or diagnostic observations only; intervention not indicated | Moderate symptoms; limiting instrumental ADL | - | - | - |
| Definition: A finding based on laboratory test results that indicate abnormal levels of prolactin hormone in a blood specimen. | | | | | |
| Carbon monoxide diffusing capacity decreased | 3 - 5 units below LLN; for follow-up, a decrease of 3 - 5 units (ml/min/mm Hg) below the baseline value | 6 - 8 units below LLN; for follow-up, an asymptomatic decrease of >5 - 8 units (ml/min/mm Hg) below the baseline value | Asymptomatic decrease of >8 units drop; >5 units drop along with the presence of pulmonary symptoms (e.g. ,  >Grade 2 hypoxia or >Grade  2 or higher dyspnea) | - | - |
| Definition: A finding based on lung function test results that indicate a decrease in the lung capacity to absorb carbon monoxide. | | | | | |
| Cardiac troponin I increased | Levels above the upper limit of normal and below the level of myocardial infarction as defined by the manufacturer | - | Levels consistent with myocardial infarction as defined by the manufacturer | - | - |
| Definition: A laboratory test result which indicates increased levels of cardiac troponin I in a biological specimen. | | | | | |

| **Investigations** | | | | | |
| --- | --- | --- | --- | --- | --- |
|  | **Grade** | | | | |
| **Adverse Event** | **1** | **2** | **3** | **4** | **5** |
| Cardiac troponin T increased | Levels above the upper limit of normal and below the level of myocardial infarction as defined by the manufacturer | - | Levels consistent with myocardial infarction as defined by the manufacturer | - | - |
| Definition: A laboratory test result which indicates increased levels of cardiac troponin T in a biological specimen. | | | | | |
| CD4 lymphocytes decreased | <LLN - 500/mm3; <LLN - 0.5  x 10e9 /L | <500 - 200/mm3; <0.5 - 0.2 x  10e9 /L | <200 - 50/mm3; <0.2 x 0.05 -  10e9 /L | <50/mm3; <0.05 x 10e9 /L | - |
| Definition: A finding based on laboratory test results that indicate an decrease in levels of CD4 lymphocytes in a blood specimen. | | | | | |
| Cholesterol high | >ULN - 300 mg/dL; >ULN -  7.75 mmol/L | >300 - 400 mg/dL; >7.75 -  10.34 mmol/L | >400 - 500 mg/dL; >10.34 -  12.92 mmol/L | >500 mg/dL; >12.92 mmol/L | - |
| Definition: A finding based on laboratory test results that indicate higher than normal levels of cholesterol in a blood specimen. | | | | | |
| CPK increased | >ULN - 2.5 x ULN | >2.5 x ULN - 5 x ULN | >5 x ULN - 10 x ULN | >10 x ULN | - |
| Definition: A finding based on laboratory test results that indicate an increase in levels of creatine phosphokinase in a blood specimen. | | | | | |
| Creatinine increased | >1 - 1.5 x baseline; >ULN -  1.5 x ULN | >1.5 - 3.0 x baseline; >1.5 -  3.0 x ULN | >3.0 baseline; >3.0 - 6.0 x ULN | >6.0 x ULN | - |
| Definition: A finding based on laboratory test results that indicate increased levels of creatinine in a biological specimen. | | | | | |
| Ejection fraction decreased | - | Resting ejection fraction (EF) 50 - 40%; 10 - 19% drop from baseline | Resting ejection fraction (EF) 39 - 20%; >20% drop from baseline | Resting ejection fraction (EF)  <20% | - |
| Definition: The percentage computed when the amount of blood ejected during a ventricular contraction of the heart is compared to the amount that was present prior to the contraction. | | | | | |

| **Investigations** | | | | | |
| --- | --- | --- | --- | --- | --- |
|  | **Grade** | | | | |
| **Adverse Event** | **1** | **2** | **3** | **4** | **5** |
| Electrocardiogram QT corrected interval prolonged | QTc 450 - 480 ms | QTc 481 - 500 ms | QTc >= 501 ms on at least two separate ECGs | QTc >= 501 or >60 ms change from baseline and Torsade de pointes or polymorphic ventricular tachycardia or signs/symptoms of serious arrhythmia | - |
| Definition: A finding of a cardiac dysrhythmia characterized by an abnormally long corrected QT interval. | | | | | |
| Fibrinogen decreased | <1.0 - 0.75 x LLN or <25%  decrease from baseline | <0.75 - 0.5 x LLN or 25 -  <50% decrease from baseline | <0.5 - 0.25 x LLN or 50 -  <75% decrease from baseline | <0.25 x LLN or 75% decrease from baseline or absolute value <50 mg/dL | - |
| Definition: A finding based on laboratory test results that indicate an decrease in levels of fibrinogen in a blood specimen. | | | | | |
| Forced expiratory volume decreased | FEV1% (percentages of observed FEV1 and FVC related to their respective predicted values) 99 - 70% predicted | FEV1 60 - 69% | 50 - 59% | <= 49% | - |
| Definition: A finding based on test results that indicate a relative decrease in the fraction of the forced vital capacity that is exhaled in a specific number of seconds. | | | | | |
| GGT increased | >ULN - 2.5 x ULN | >2.5 - 5.0 x ULN | >5.0 - 20.0 x ULN | >20.0 x ULN | - |
| Definition: A finding based on laboratory test results that indicate higher than normal levels of the enzyme gamma-glutamyltransferase in the blood specimen. GGT (gamma- glutamyltransferase ) catalyzes the transfer of a gamma glutamyl group from a gamma glutamyl peptide to another peptide, amino acids or water. | | | | | |

| **Investigations** | | | | | |
| --- | --- | --- | --- | --- | --- |
|  | **Grade** | | | | |
| **Adverse Event** | **1** | **2** | **3** | **4** | **5** |
| Growth hormone abnormal | Asymptomatic; clinical or diagnostic observations only; intervention not indicated | Symptomatic; medical intervention indicated; limiting instrumental ADL | - | - | - |
| Definition: A finding based on laboratory test results that indicate abnormal levels of growth hormone in a biological specimen. | | | | | |
| Haptoglobin decreased | <LLN | - | - | - | - |
| Definition: A finding based on laboratory test results that indicate an decrease in levels of haptoglobin in a blood specimen. | | | | | |
| Hemoglobin increased | Increase in >0 - 2 gm/dL above ULN or above baseline if baseline is above ULN | Increase in >2 - 4 gm/dL above ULN or above baseline if baseline is above ULN | Increase in >4 gm/dL above ULN or above baseline if baseline is above ULN | - | - |
| Definition: A finding based on laboratory test results that indicate increased levels of hemoglobin in a biological specimen. | | | | | |
| INR increased | >1 - 1.5 x ULN; >1 - 1.5 times  above baseline if on anticoagulation | >1.5 - 2.5 x ULN; >1.5 - 2.5  times above baseline if on anticoagulation | >2.5 x ULN; >2.5 times above baseline if on anticoagulation | - | - |
| Definition: A finding based on laboratory test results that indicate an increase in the ratio of the patient's prothrombin time to a control sample in the blood. | | | | | |
| Lipase increased | >ULN - 1.5 x ULN | >1.5 - 2.0 x ULN | >2.0 - 5.0 x ULN | >5.0 x ULN | - |
| Definition: A finding based on laboratory test results that indicate an increase in the level of lipase in a biological specimen. | | | | | |
| Lymphocyte count decreased | <LLN - 800/mm3; <LLN - 0.8  x 10e9 /L | <800 - 500/mm3; <0.8 - 0.5 x  10e9 /L | <500 - 200/mm3; <0.5 - 0.2 x  10e9 /L | <200/mm3; <0.2 x 10e9 /L | - |
| Definition: A finding based on laboratory test results that indicate a decrease in number of lymphocytes in a blood specimen. | | | | | |
| Lymphocyte count increased | - | >4000/mm3 - 20,000/mm3 | >20,000/mm3 | - | - |
| Definition: A finding based on laboratory test results that indicate an abnormal increase in the number of lymphocytes in the blood, effusions or bone marrow. | | | | | |

| **Investigations** | | | | | |
| --- | --- | --- | --- | --- | --- |
|  | **Grade** | | | | |
| **Adverse Event** | **1** | **2** | **3** | **4** | **5** |
| Neutrophil count decreased | <LLN - 1500/mm3; <LLN - 1.5  x 10e9 /L | <1500 - 1000/mm3; <1.5 - 1.0  x 10e9 /L | <1000 - 500/mm3; <1.0 - 0.5 x  10e9 /L | <500/mm3; <0.5 x 10e9 /L | - |
| Definition: A finding based on laboratory test results that indicate a decrease in number of neutrophils in a blood specimen. | | | | | |
| Pancreatic enzymes decreased | <LLN and asymptomatic | Increase in stool frequency, bulk, or odor; steatorrhea | Sequelae of absorption deficiency | - | - |
| Definition: A finding based on laboratory test results that indicate an decrease in levels of pancreatic enzymes in a biological specimen. | | | | | |
| Platelet count decreased | <LLN - 75,000/mm3; <LLN -  75.0 x 10e9 /L | <75,000 - 50,000/mm3; <75.0  - 50.0 x 10e9 /L | <50,000 - 25,000/mm3; <50.0  - 25.0 x 10e9 /L | <25,000/mm3; <25.0 x 10e9  /L | - |
| Definition: A finding based on laboratory test results that indicate a decrease in number of platelets in a blood specimen. | | | | | |
| Serum amylase increased | >ULN - 1.5 x ULN | >1.5 - 2.0 x ULN | >2.0 - 5.0 x ULN | >5.0 x ULN | - |
| Definition: A finding based on laboratory test results that indicate an increase in the levels of amylase in a serum specimen. | | | | | |
| Urine output decreased | - | - | Oliguria (<80 ml in 8 hr) | Anuria (<240 ml in 24 hr) | - |
| Definition: A finding based on test results that indicate urine production is less relative to previous output. | | | | | |
| Vital capacity abnormal | 90 - 75% of predicted value | <75 - 50% of predicted value; limiting instrumental ADL | <50% of predicted value; limiting self care ADL | - | - |
| Definition: A finding based on pulmonary function test results that indicate an abnormal vital capacity (amount of exhaled after a maximum inhalation) when compared to the predicted value. | | | | | |
| Weight gain | 5 - <10% from baseline | 10 - <20% from baseline | >=20% from baseline | - | - |
| Definition: A finding characterized by an increase in overall body weight; for pediatrics, greater than the baseline growth curve. | | | | | |
| Weight loss | 5 to <10% from baseline; intervention not indicated | 10 - <20% from baseline; nutritional support indicated | >=20% from baseline; tube feeding or TPN indicated | - | - |

| **Investigations** | | | | | |
| --- | --- | --- | --- | --- | --- |
|  | **Grade** | | | | |
| **Adverse Event** | **1** | **2** | **3** | **4** | **5** |
| Definition: A finding characterized by a decrease in overall body weight; for pediatrics, less than the baseline growth curve. | | | | | |
| White blood cell decreased | <LLN - 3000/mm3; <LLN - 3.0  x 10e9 /L | <3000 - 2000/mm3; <3.0 - 2.0  x 10e9 /L | <2000 - 1000/mm3; <2.0 - 1.0  x 10e9 /L | <1000/mm3; <1.0 x 10e9 /L | - |
| Definition: A finding based on laboratory test results that indicate an decrease in number of white blood cells in a blood specimen. | | | | | |
| Investigations - Other, specify | Asymptomatic or mild symptoms; clinical or diagnostic observations only; intervention not indicated | Moderate; minimal, local or noninvasive intervention indicated; limiting age- appropriate instrumental ADL | Severe or medically significant but not immediately life- threatening; hospitalization or prolongation of existing hospitalization indicated; disabling; limiting self care ADL | Life-threatening consequences; urgent intervention indicated | Death |

| **Blood and lymphatic system disorders** | | | | | |
| --- | --- | --- | --- | --- | --- |
|  | **Grade** | | | | |
| **Adverse Event** | **1** | **2** | **3** | **4** | **5** |
| Anemia | Hemoglobin (Hgb) <LLN -  10.0 g/dL; <LLN - 6.2 mmol/L;  <LLN - 100 g/L | Hgb <10.0 - 8.0 g/dL; <6.2 -  4.9 mmol/L; <100 - 80g/L | Hgb <8.0 g/dL; <4.9 mmol/L;  <80 g/L; transfusion indicated | Life-threatening consequences; urgent intervention indicated | Death |
| Definition: A disorder characterized by an reduction in the amount of hemoglobin in 100 ml of blood. Signs and symptoms of anemia may include pallor of the skin and mucous membranes, shortness of breath, palpitations of the heart, soft systolic murmurs, lethargy, and fatigability. | | | | | |
| Bone marrow hypocellular | Mildly hypocellular or <=25% reduction from normal cellularity for age | Moderately hypocellular or  >25 - <50% reduction from normal cellularity for age | Severely hypocellular or >50 -  <=75% reduction cellularity from normal for age | Aplastic persistent for longer than 2 weeks | Death |
| Definition: A disorder characterized by the inability of the bone marrow to produce hematopoietic elements. | | | | | |
| Disseminated intravascular coagulation | - | Laboratory findings with no bleeding | Laboratory findings and bleeding | Life-threatening consequences; urgent intervention indicated | Death |
| Definition: A disorder characterized by systemic pathological activation of blood clotting mechanisms which results in clot formation throughout the body. There is an increase in the risk of hemorrhage as the body is depleted of platelets and coagulation factors. | | | | | |
| Febrile neutropenia | - | - | ANC <1000/mm3 with a single temperature of >38.3 degrees C (101 degrees F) or a sustained temperature of  >=38 degrees C (100.4 degrees F) for more than one  hour. | Life-threatening consequences; urgent intervention indicated | Death |
| Definition: A disorder characterized by an ANC <1000/mm3 and a single temperature of >38.3 degrees C (101 degrees F) or a sustained temperature of >=38 degrees C (100.4 degrees F) for more than one hour. | | | | | |

| **Blood and lymphatic system disorders** | | | | | |
| --- | --- | --- | --- | --- | --- |
|  | **Grade** | | | | |
| **Adverse Event** | **1** | **2** | **3** | **4** | **5** |
| Hemolysis | Laboratory evidence of hemolysis only (e.g., direct antiglobulin test; DAT; Coombs'; schistocytes; decreased haptoglobin) | Evidence of hemolysis and  >=2 gm decrease in hemoglobin. | Transfusion or medical intervention indicated (e.g., steroids) | Life-threatening consequences; urgent intervention indicated | Death |
| Definition: A disorder characterized by laboratory test results that indicate widespread erythrocyte cell membrane destruction. | | | | | |
| Hemolytic uremic syndrome | Evidence of RBC destruction (schistocytosis) without clinical consequences | - | Laboratory findings with clinical consequences (e.g., renal insufficiency, petechiae) | Life-threatening consequences, (e.g., CNS hemorrhage or thrombosis/embolism or renal failure) | Death |
| Definition: A disorder characterized by a form of thrombotic microangiopathy with renal failure, hemolytic anemia, and severe thrombocytopenia. | | | | | |
| Leukocytosis | - | - | >100,000/mm3 | Clinical manifestations of leucostasis; urgent intervention indicated | Death |
| Definition: A disorder characterized by laboratory test results that indicate an increased number of white blood cells in the blood. | | | | | |
| Lymph node pain | Mild pain | Moderate pain; limiting instrumental ADL | Severe pain; limiting self care ADL | - | - |
| Definition: A disorder characterized by a sensation of marked discomfort in a lymph node. | | | | | |
| Spleen disorder | Incidental findings (e.g., Howell-Jolly bodies); mild degree of thrombocytosis and leukocytosis | Prophylactic antibiotics indicated | - | Life-threatening consequences; urgent intervention indicated | Death |

| **Blood and lymphatic system disorders** | | | | | |
| --- | --- | --- | --- | --- | --- |
|  | **Grade** | | | | |
| **Adverse Event** | **1** | **2** | **3** | **4** | **5** |
| Definition: A disorder of the spleen. | | | | | |
| Thrombotic thrombocytopenic purpura | Evidence of RBC destruction (schistocytosis) without clinical consequences | - | Laboratory findings with clinical consequences (e.g., renal insufficiency, petechiae) | Life-threatening consequences, (e.g., CNS hemorrhage or thrombosis/embolism or renal failure) | Death |
| Definition: A disorder characterized by the presence of microangiopathic hemolytic anemia, thrombocytopenic purpura, fever, renal abnormalities and neurological abnormalities such as seizures, hemiplegia, and visual disturbances. It is an acute or subacute condition. | | | | | |
| Blood and lymphatic system disorders - Other, specify | Asymptomatic or mild symptoms; clinical or diagnostic observations only; intervention not indicated | Moderate; minimal, local or noninvasive intervention indicated; limiting age- appropriate instrumental ADL | Severe or medically significant but not immediately life- threatening; hospitalization or prolongation of existing hospitalization indicated; disabling; limiting self care ADL | Life-threatening consequences; urgent intervention indicated | Death |

| **Gastrointestinal disorders** | | | | | |
| --- | --- | --- | --- | --- | --- |
|  | **Grade** | | | | |
| **Adverse Event** | **1** | **2** | **3** | **4** | **5** |
| Abdominal distension | Asymptomatic; clinical or diagnostic observations only; intervention not indicated | Symptomatic; limiting instrumental ADL | Severe discomfort; limiting self care ADL | - | - |
| Definition: A disorder characterized by swelling of the abdomen. | | | | | |
| Abdominal pain | Mild pain | Moderate pain; limiting instrumental ADL | Severe pain; limiting self care ADL | - | - |
| Definition: A disorder characterized by a sensation of marked discomfort in the abdominal region. | | | | | |
| Anal fistula | Asymptomatic; clinical or diagnostic observations only; intervention not indicated | Symptomatic; altered GI function | Severely altered GI function; tube feeding, TPN or hospitalization indicated; elective operative intervention indicated | Life-threatening consequences; urgent intervention indicated | Death |
| Definition: A disorder characterized by an abnormal communication between the opening in the anal canal to the perianal skin. | | | | | |
| Anal hemorrhage | Mild; intervention not indicated | Moderate symptoms; medical intervention or minor cauterization indicated | Transfusion, radiologic, endoscopic, or elective operative intervention indicated | Life-threatening consequences; urgent intervention indicated | Death |
| Definition: A disorder characterized by bleeding from the anal region. | | | | | |
| Anal mucositis | Asymptomatic or mild symptoms; intervention not indicated | Symptomatic; medical intervention indicated; limiting instrumental ADL | Severe symptoms; limiting self care ADL | Life-threatening consequences; urgent intervention indicated | Death |
| Definition: A disorder characterized by inflammation of the mucous membrane of the anus. | | | | | |

| **Gastrointestinal disorders** | | | | | |
| --- | --- | --- | --- | --- | --- |
|  | **Grade** | | | | |
| **Adverse Event** | **1** | **2** | **3** | **4** | **5** |
| Anal necrosis | - | - | TPN or hospitalization indicated; radiologic, endoscopic, or operative intervention indicated | Life-threatening consequences; urgent operative intervention indicated | Death |
| Definition: A disorder characterized by a necrotic process occurring in the anal region. | | | | | |
| Anal pain | Mild pain | Moderate pain; limiting instrumental ADL | Severe pain; limiting self care ADL | - | - |
| Definition: A disorder characterized by a sensation of marked discomfort in the anal region. | | | | | |
| Anal stenosis | Asymptomatic; clinical or diagnostic observations only; intervention not indicated | Symptomatic; altered GI function | Symptomatic and severely altered GI function; non- emergent operative intervention indicated; TPN or hospitalization indicated | Life-threatening consequences; urgent operative intervention indicated | Death |
| Definition: A disorder characterized by a narrowing of the lumen of the anal canal. | | | | | |
| Anal ulcer | Asymptomatic; clinical or diagnostic observations only; intervention not indicated | Symptomatic; altered GI function | Severely altered GI function; TPN indicated; elective operative or endoscopic intervention indicated; disabling | Life-threatening consequences; urgent operative intervention indicated | Death |
| Definition: A disorder characterized by a circumscribed, inflammatory and necrotic erosive lesion on the mucosal surface of the anal canal. | | | | | |

| **Gastrointestinal disorders** | | | | | |
| --- | --- | --- | --- | --- | --- |
|  | **Grade** | | | | |
| **Adverse Event** | **1** | **2** | **3** | **4** | **5** |
| Ascites | Asymptomatic; clinical or diagnostic observations only; intervention not indicated | Symptomatic; medical intervention indicated | Severe symptoms; invasive intervention indicated | Life-threatening consequences; urgent operative intervention indicated | Death |
| Definition: A disorder characterized by accumulation of serous or hemorrhagic fluid in the peritoneal cavity. | | | | | |
| Bloating | No change in bowel function or oral intake | Symptomatic, decreased oral intake; change in bowel function | - | - | - |
| Definition: A disorder characterized by subject-reported feeling of uncomfortable fullness of the abdomen. | | | | | |
| Cecal hemorrhage | Mild; intervention not indicated | Moderate symptoms; medical intervention or minor cauterization indicated | Transfusion, radiologic, endoscopic, or elective operative intervention indicated | Life-threatening consequences; urgent intervention indicated | Death |
| Definition: A disorder characterized by bleeding from the cecum. | | | | | |
| Cheilitis | Asymptomatic; clinical or diagnostic observations only; intervention not indicated | Moderate symptoms; limiting instrumental ADL | Severe symptoms; limiting self care ADL; intervention indicated | - | - |
| Definition: A disorder characterized by inflammation of the lip. | | | | | |
| Colitis | Asymptomatic; clinical or diagnostic observations only; intervention not indicated | Abdominal pain; mucus or blood in stool | Severe abdominal pain; change in bowel habits; medical intervention indicated; peritoneal signs | Life-threatening consequences; urgent intervention indicated | Death |
| Definition: A disorder characterized by inflammation of the colon. | | | | | |

| **Gastrointestinal disorders** | | | | | |
| --- | --- | --- | --- | --- | --- |
|  | **Grade** | | | | |
| **Adverse Event** | **1** | **2** | **3** | **4** | **5** |
| Colonic fistula | Asymptomatic; clinical or diagnostic observations only; intervention not indicated | Symptomatic; altered GI function | Severely altered GI function; bowel rest, TPN or hospitalization indicated; elective operative intervention indicated | Life-threatening consequences; urgent intervention indicated | Death |
| Definition: A disorder characterized by an abnormal communication between the large intestine and another organ or anatomic site. | | | | | |
| Colonic hemorrhage | Mild; intervention not indicated | Moderate symptoms; medical intervention or minor cauterization indicated | Transfusion, radiologic, endoscopic, or elective operative intervention indicated | Life-threatening consequences; urgent intervention indicated | Death |
| Definition: A disorder characterized by bleeding from the colon. | | | | | |
| Colonic obstruction | Asymptomatic; clinical or diagnostic observations only; intervention not indicated | Symptomatic; altered GI function | Hospitalization indicated; elective operative intervention indicated; disabling | Life-threatening consequences; urgent operative intervention indicated | Death |
| Definition: A disorder characterized by blockage of the normal flow of the intestinal contents in the colon. | | | | | |
| Colonic perforation | - | Symptomatic; medical intervention indicated | Severe symptoms; elective operative intervention indicated | Life-threatening consequences; urgent intervention indicated | Death |
| Definition: A disorder characterized by a rupture in the colonic wall. | | | | | |

| **Gastrointestinal disorders** | | | | | |
| --- | --- | --- | --- | --- | --- |
|  | **Grade** | | | | |
| **Adverse Event** | **1** | **2** | **3** | **4** | **5** |
| Colonic stenosis | Asymptomatic; clinical or diagnostic observations only; intervention not indicated | Symptomatic; altered GI function | Severely altered GI function; tube feeding or hospitalization indicated; elective operative intervention indicated | Life-threatening consequences; urgent operative intervention indicated | Death |
| Definition: A disorder characterized by a narrowing of the lumen of the colon. | | | | | |
| Colonic ulcer | Asymptomatic; clinical or diagnostic observations only; intervention not indicated | Symptomatic; altered GI function | Severely altered GI function; TPN indicated; elective operative or endoscopic intervention indicated; disabling | Life-threatening consequences; urgent operative intervention indicated | Death |
| Definition: A disorder characterized by a circumscribed, inflammatory and necrotic erosive lesion on the mucosal surface of the colon. | | | | | |
| Constipation | Occasional or intermittent symptoms; occasional use of stool softeners, laxatives, dietary modification, or enema | Persistent symptoms with regular use of laxatives or enemas; limiting instrumental ADL | Obstipation with manual evacuation indicated; limiting self care ADL | Life-threatening consequences; urgent intervention indicated | Death |
| Definition: A disorder characterized by irregular and infrequent or difficult evacuation of the bowels. | | | | | |
| Dental caries | One or more dental caries, not involving the root | Dental caries involving the root | Dental caries resulting in pulpitis or periapical abscess or resulting in tooth loss | - | - |
| Definition: A disorder characterized by the decay of a tooth, in which it becomes softened, discolored and/or porous. | | | | | |

| **Gastrointestinal disorders** | | | | | |
| --- | --- | --- | --- | --- | --- |
|  | **Grade** | | | | |
| **Adverse Event** | **1** | **2** | **3** | **4** | **5** |
| Diarrhea | Increase of <4 stools per day over baseline; mild increase in ostomy output compared to baseline | Increase of 4 - 6 stools per day over baseline; moderate increase in ostomy output compared to baseline | Increase of >=7 stools per day over baseline; incontinence; hospitalization indicated; severe increase in ostomy output compared to baseline; limiting self care ADL | Life-threatening consequences; urgent intervention indicated | Death |
| Definition: A disorder characterized by frequent and watery bowel movements. | | | | | |
| Dry mouth | Symptomatic (e.g., dry or thick saliva) without significant dietary alteration; unstimulated saliva flow >0.2 ml/min | Moderate symptoms; oral intake alterations (e.g., copious water, other lubricants, diet limited to purees and/or soft, moist foods); unstimulated saliva 0.1 to 0.2 ml/min | Inability to adequately aliment orally; tube feeding or TPN indicated; unstimulated saliva  <0.1 ml/min | - | - |
| Definition: A disorder characterized by reduced salivary flow in the oral cavity. | | | | | |
| Duodenal fistula | Asymptomatic; clinical or diagnostic observations only; intervention not indicated | Symptomatic; altered GI function | Severely altered GI function; tube feeding, TPN or hospitalization indicated; elective operative intervention indicated | Life-threatening consequences; urgent intervention indicated | Death |
| Definition: A disorder characterized by an abnormal communication between the duodenum and another organ or anatomic site. | | | | | |

| **Gastrointestinal disorders** | | | | | |
| --- | --- | --- | --- | --- | --- |
|  | **Grade** | | | | |
| **Adverse Event** | **1** | **2** | **3** | **4** | **5** |
| Duodenal hemorrhage | Mild; intervention not indicated | Moderate symptoms; medical intervention or minor cauterization indicated | Transfusion, radiologic, endoscopic, or elective operative intervention indicated | Life-threatening consequences; urgent intervention indicated | Death |
| Definition: A disorder characterized by bleeding from the duodenum. | | | | | |
| Duodenal obstruction | Asymptomatic; clinical or diagnostic observations only; intervention not indicated | Symptomatic; altered GI function | Hospitalization or elective operative intervention indicated; disabling | Life-threatening consequences; urgent operative intervention indicated | Death |
| Definition: A disorder characterized by blockage of the normal flow of stomach contents through the duodenum. | | | | | |
| Duodenal perforation | - | Symptomatic; medical intervention indicated | Severe symptoms; elective operative intervention indicated | Life-threatening consequences; urgent operative intervention indicated | Death |
| Definition: A disorder characterized by a rupture in the duodenal wall. | | | | | |
| Duodenal stenosis | Asymptomatic; clinical or diagnostic observations only; intervention not indicated | Symptomatic; altered GI function | Severely altered GI function; tube feeding; hospitalization indicated; elective operative intervention indicated | Life-threatening consequences; urgent operative intervention indicated | Death |
| Definition: A disorder characterized by a narrowing of the lumen of the duodenum. | | | | | |

| **Gastrointestinal disorders** | | | | | |
| --- | --- | --- | --- | --- | --- |
|  | **Grade** | | | | |
| **Adverse Event** | **1** | **2** | **3** | **4** | **5** |
| Duodenal ulcer | Asymptomatic; clinical or diagnostic observations only; intervention not indicated | Moderate symptoms; medical intervention indicated; limiting instrumental ADL | Severely altered GI function; TPN indicated; elective operative or endoscopic intervention indicated; limiting self care ADL; disabling | Life-threatening consequences; urgent operative intervention indicated | Death |
| Definition: A disorder characterized by a circumscribed, inflammatory and necrotic erosive lesion on the mucosal surface of the duodenal wall. | | | | | |
| Dyspepsia | Mild symptoms; intervention not indicated | Moderate symptoms; medical intervention indicated | Severe symptoms; surgical intervention indicated | - | - |
| Definition: A disorder characterized by an uncomfortable, often painful feeling in the stomach, resulting from impaired digestion. Symptoms include burning stomach, bloating, heartburn, nausea and vomiting. | | | | | |
| Dysphagia | Symptomatic, able to eat regular diet | Symptomatic and altered eating/swallowing | Severely altered eating/swallowing; tube feeding or TPN or hospitalization indicated | Life-threatening consequences; urgent intervention indicated | Death |
| Definition: A disorder characterized by difficulty in swallowing. | | | | | |
| Enterocolitis | Asymptomatic; clinical or diagnostic observations only; intervention not indicated | Abdominal pain; mucus or blood in stool | Severe or persistent abdominal pain; fever; ileus; peritoneal signs | Life-threatening consequences; urgent intervention indicated | Death |
| Definition: A disorder characterized by inflammation of the small and large intestines. | | | | | |
| Enterovesical fistula | Asymptomatic; clinical or diagnostic observations only; intervention not indicated | Symptomatic; noninvasive intervention indicated | Severe, medically significant; medical intervention indicated | Life-threatening consequences; urgent intervention indicated | Death |
| Definition: A disorder characterized by an abnormal communication between the urinary bladder and the intestine. | | | | | |

| **Gastrointestinal disorders** | | | | | |
| --- | --- | --- | --- | --- | --- |
|  | **Grade** | | | | |
| **Adverse Event** | **1** | **2** | **3** | **4** | **5** |
| Esophageal fistula | Asymptomatic; clinical or diagnostic observations only; intervention not indicated | Symptomatic; altered GI function | Severely altered GI function; tube feeding, TPN or hospitalization indicated; elective operative intervention indicated | Life-threatening consequences; urgent intervention indicated | Death |
| Definition: A disorder characterized by an abnormal communication between the esophagus and another organ or anatomic site. | | | | | |
| Esophageal hemorrhage | Mild; intervention not indicated | Moderate symptoms; medical intervention or minor cauterization indicated | Transfusion, radiologic, endoscopic, or elective operative intervention indicated | Life-threatening consequences; urgent intervention indicated | Death |
| Definition: A disorder characterized by bleeding from the esophagus. | | | | | |
| Esophageal necrosis | - | - | Inability to aliment adequately by GI tract; radiologic, endoscopic, or operative intervention indicated | Life-threatening consequences; urgent operative intervention indicated | Death |
| Definition: A disorder characterized by a necrotic process occurring in the esophageal wall. | | | | | |
| Esophageal obstruction | Asymptomatic; clinical or diagnostic observations only; intervention not indicated | Symptomatic; altered GI function; limiting instrumental ADL | Hospitalization indicated; elective operative intervention indicated; limiting self care ADL; disabling | Life-threatening consequences; urgent intervention indicated | Death |
| Definition: A disorder characterized by blockage of the normal flow of the contents in the esophagus. | | | | | |
| Esophageal pain | Mild pain | Moderate pain; limiting instrumental ADL | Severe pain; limiting self care ADL | - | - |

| **Gastrointestinal disorders** | | | | | |
| --- | --- | --- | --- | --- | --- |
|  | **Grade** | | | | |
| **Adverse Event** | **1** | **2** | **3** | **4** | **5** |
| Definition: A disorder characterized by a sensation of marked discomfort in the esophageal region. | | | | | |
| Esophageal perforation | - | Symptomatic; medical intervention indicated | Severe symptoms; elective operative intervention indicated | Life-threatening consequences; urgent operative intervention indicated | Death |
| Definition: A disorder characterized by a rupture in the wall of the esophagus. | | | | | |
| Esophageal stenosis | Asymptomatic; clinical or diagnostic observations only; intervention not indicated | Symptomatic; altered GI function | Severely altered GI function; tube feeding; hospitalization indicated; elective operative intervention indicated | Life-threatening consequences; urgent operative intervention indicated | Death |
| Definition: A disorder characterized by a narrowing of the lumen of the esophagus. | | | | | |
| Esophageal ulcer | Asymptomatic; clinical or diagnostic observations only; intervention not indicated | Symptomatic; altered GI function; limiting instrumental ADL | Severely altered GI function; TPN indicated; elective operative or endoscopic intervention indicated; limiting self care ADL; disabling | Life-threatening consequences; urgent operative intervention indicated | Death |
| Definition: A disorder characterized by a circumscribed, inflammatory and necrotic erosive lesion on the mucosal surface of the esophageal wall. | | | | | |
| Esophageal varices hemorrhage | - | Self-limited; intervention not indicated | Transfusion, radiologic, endoscopic, or elective operative intervention indicated | Life-threatening consequences; urgent intervention indicated | Death |
| Definition: A disorder characterized by bleeding from esophageal varices. | | | | | |

| **Gastrointestinal disorders** | | | | | |
| --- | --- | --- | --- | --- | --- |
|  | **Grade** | | | | |
| **Adverse Event** | **1** | **2** | **3** | **4** | **5** |
| Esophagitis | Asymptomatic; clinical or diagnostic observations only; intervention not indicated | Symptomatic; altered eating/swallowing; oral supplements indicated | Severely altered eating/swallowing; tube feeding, TPN or hospitalization indicated | Life-threatening consequences; urgent operative intervention indicated | Death |
| Definition: A disorder characterized by inflammation of the esophageal wall. | | | | | |
| Fecal incontinence | Occasional use of pads required | Daily use of pads required | Severe symptoms; elective operative intervention indicated | - | - |
| Definition: A disorder characterized by inability to control the escape of stool from the rectum. | | | | | |
| Flatulence | Mild symptoms; intervention not indicated | Moderate; persistent; psychosocial sequelae | - | - | - |
| Definition: A disorder characterized by a state of excessive gas in the alimentary canal. | | | | | |
| Gastric fistula | Asymptomatic; clinical or diagnostic observations only; intervention not indicated | Symptomatic; altered GI function | Severely altered GI function; bowel rest; tube feeding, TPN or hospitalization indicated; elective operative intervention indicated | Life-threatening consequences; urgent operative intervention indicated | Death |
| Definition: A disorder characterized by an abnormal communication between the stomach and another organ or anatomic site. | | | | | |
| Gastric hemorrhage | Mild; intervention not indicated | Moderate symptoms; medical intervention or minor cauterization indicated | Transfusion, radiologic, endoscopic, or elective operative intervention indicated | Life-threatening consequences; urgent intervention indicated | Death |
| Definition: A disorder characterized by bleeding from the gastric wall. | | | | | |

| **Gastrointestinal disorders** | | | | | |
| --- | --- | --- | --- | --- | --- |
|  | **Grade** | | | | |
| **Adverse Event** | **1** | **2** | **3** | **4** | **5** |
| Gastric necrosis | - | - | Inability to aliment adequately by GI tract; radiologic, endoscopic, or operative intervention indicated | Life-threatening consequences; urgent operative intervention indicated | Death |
| Definition: A disorder characterized by a necrotic process occurring in the gastric wall. | | | | | |
| Gastric perforation | - | Symptomatic; medical intervention indicated | Severe symptoms; elective operative intervention indicated | Life-threatening consequences; urgent operative intervention indicated | Death |
| Definition: A disorder characterized by a rupture in the stomach wall. | | | | | |
| Gastric stenosis | Asymptomatic; clinical or diagnostic observations only; intervention not indicated | Symptomatic; altered GI function | Severely altered GI function; tube feeding; hospitalization indicated; elective operative intervention indicated | Life-threatening consequences; urgent operative intervention indicated | Death |
| Definition: A disorder characterized by a narrowing of the lumen of the stomach. | | | | | |
| Gastric ulcer | Asymptomatic; clinical or diagnostic observations only; intervention not indicated | Symptomatic; altered GI function; medical intervention indicated; limiting instrumental ADL | Severely altered GI function; TPN indicated; elective operative or endoscopic intervention indicated; limiting self care ADL; disabling | Life-threatening consequences; urgent operative intervention indicated | Death |
| Definition: A disorder characterized by a circumscribed, inflammatory and necrotic erosive lesion on the mucosal surface of the stomach. | | | | | |

| **Gastrointestinal disorders** | | | | | |
| --- | --- | --- | --- | --- | --- |
|  | **Grade** | | | | |
| **Adverse Event** | **1** | **2** | **3** | **4** | **5** |
| Gastritis | Asymptomatic; clinical or diagnostic observations only; intervention not indicated | Symptomatic; altered GI function; medical intervention indicated | Severely altered eating or gastric function; TPN or hospitalization indicated | Life-threatening consequences; urgent operative intervention indicated | Death |
| Definition: A disorder characterized by inflammation of the stomach. | | | | | |
| Gastroesophageal reflux disease | Mild symptoms; intervention not indicated | Moderate symptoms; medical intervention indicated | Severe symptoms; surgical intervention indicated | - | - |
| Definition: A disorder characterized by reflux of the gastric and/or duodenal contents into the distal esophagus. It is chronic in nature and usually caused by incompetence of the lower esophageal sphincter, and may result in injury to the esophageal mucosal. Symptoms include heartburn and acid indigestion. | | | | | |
| Gastrointestinal fistula | Asymptomatic; clinical or diagnostic observations only; intervention not indicated | Symptomatic; altered GI function | Severely altered GI function; tube feeding, TPN or hospitalization indicated | Life-threatening consequences; urgent operative intervention indicated | Death |
| Definition: A disorder characterized by an abnormal communication between any part of the gastrointestinal system and another organ or anatomic site. | | | | | |
| Gastrointestinal pain | Mild pain | Moderate pain; limiting instrumental ADL | Severe pain; limiting self care ADL | - | - |
| Definition: A disorder characterized by a sensation of marked discomfort in the gastrointestinal region. | | | | | |
| Gastroparesis | Mild nausea, early satiety and bloating, able to maintain caloric intake on regular diet | Moderate symptoms; able to maintain nutrition with dietary and lifestyle modifications; may need pharmacologic intervention | Weight loss; refractory to medical intervention; unable to maintain nutrition orally | - | - |

| **Gastrointestinal disorders** | | | | | |
| --- | --- | --- | --- | --- | --- |
|  | **Grade** | | | | |
| **Adverse Event** | **1** | **2** | **3** | **4** | **5** |
| Definition: A disorder characterized by an incomplete paralysis of the muscles of the stomach wall resulting in delayed emptying of the gastric contents into the small intestine. | | | | | |
| Gingival pain | Mild pain | Moderate pain interfering with oral intake | Severe pain; inability to aliment orally | - | - |
| Definition: A disorder characterized by a sensation of marked discomfort in the gingival region. | | | | | |
| Hemorrhoidal hemorrhage | Mild; intervention not indicated | Moderate symptoms; medical intervention or minor cauterization indicated | Transfusion, radiologic, endoscopic, or elective operative intervention indicated | Life-threatening consequences; urgent intervention indicated | Death |
| Definition: A disorder characterized by bleeding from the hemorrhoids. | | | | | |
| Hemorrhoids | Asymptomatic; clinical or diagnostic observations only; intervention not indicated | Symptomatic; banding or medical intervention indicated | Severe symptoms; radiologic, endoscopic or elective operative intervention indicated | - | - |
| Definition: A disorder characterized by the presence of dilated veins in the rectum and surrounding area. | | | | | |
| Ileal fistula | Asymptomatic; clinical or diagnostic observations only; intervention not indicated | Symptomatic; altered GI function | Severely altered GI function; TPN or hospitalization indicated; elective operative intervention indicated | Life-threatening consequences; urgent intervention indicated | Death |
| Definition: A disorder characterized by an abnormal communication between the ileum and another organ or anatomic site. | | | | | |

| **Gastrointestinal disorders** | | | | | |
| --- | --- | --- | --- | --- | --- |
|  | **Grade** | | | | |
| **Adverse Event** | **1** | **2** | **3** | **4** | **5** |
| Ileal hemorrhage | Mild; intervention not indicated | Moderate symptoms; medical intervention or minor cauterization indicated | Transfusion, radiologic, endoscopic, or elective operative intervention indicated | Life-threatening consequences; urgent intervention indicated | Death |
| Definition: A disorder characterized by bleeding from the ileal wall. | | | | | |
| Ileal obstruction | Asymptomatic; clinical or diagnostic observations only; intervention not indicated | Symptomatic; altered GI function; limiting instrumental ADL | Hospitalization indicated; elective operative intervention indicated; limiting self care ADL; disabling | Life-threatening consequences; urgent operative intervention indicated | Death |
| Definition: A disorder characterized by blockage of the normal flow of the intestinal contents in the ileum. | | | | | |
| Ileal perforation | - | Symptomatic; medical intervention indicated | Severe symptoms; elective operative intervention indicated | Life-threatening consequences; urgent operative intervention indicated | Death |
| Definition: A disorder characterized by a rupture in the ileal wall. | | | | | |
| Ileal stenosis | Asymptomatic; clinical or diagnostic observations only; intervention not indicated | Symptomatic; altered GI function | Severely altered GI function; tube feeding or hospitalization indicated; elective operative intervention indicated | Life-threatening consequences; urgent operative intervention indicated | Death |
| Definition: A disorder characterized by a narrowing of the lumen of the ileum. | | | | | |

| **Gastrointestinal disorders** | | | | | |
| --- | --- | --- | --- | --- | --- |
|  | **Grade** | | | | |
| **Adverse Event** | **1** | **2** | **3** | **4** | **5** |
| Ileal ulcer | Asymptomatic; clinical or diagnostic observations only; intervention not indicated | Symptomatic; altered GI function | Severely altered GI function; TPN indicated; elective operative or endoscopic intervention indicated; disabling | Life-threatening consequences; urgent operative intervention indicated | Death |
| Definition: A disorder characterized by a circumscribed, inflammatory and necrotic erosive lesion on the mucosal surface of the ileum. | | | | | |
| Ileus | - | Symptomatic; altered GI function; bowel rest indicated | Severely altered GI function; TPN indicated | Life-threatening consequences; urgent intervention indicated | Death |
| Definition: A disorder characterized by failure of the ileum to transport intestinal contents. | | | | | |
| Intra-abdominal hemorrhage | - | Medical intervention or minor cauterization indicated | Transfusion, radiologic, endoscopic, or elective operative intervention indicated | Life-threatening consequences; urgent intervention indicated | Death |
| Definition: A disorder characterized by bleeding in the abdominal cavity. | | | | | |
| Jejunal fistula | Asymptomatic; clinical or diagnostic observations only; intervention not indicated | Symptomatic; altered GI function | Severely altered GI function; TPN or hospitalization indicated; elective operative intervention indicated | Life-threatening consequences; urgent intervention indicated | Death |
| Definition: A disorder characterized by an abnormal communication between the jejunum and another organ or anatomic site. | | | | | |

| **Gastrointestinal disorders** | | | | | |
| --- | --- | --- | --- | --- | --- |
|  | **Grade** | | | | |
| **Adverse Event** | **1** | **2** | **3** | **4** | **5** |
| Jejunal hemorrhage | Mild; intervention not indicated | Moderate symptoms; medical intervention or minor cauterization indicated | Transfusion, radiologic, endoscopic, or elective operative intervention indicated | Life-threatening consequences; urgent intervention indicated | Death |
| Definition: A disorder characterized by bleeding from the jejunal wall. | | | | | |
| Jejunal obstruction | Asymptomatic; clinical or diagnostic observations only; intervention not indicated | Symptomatic; altered GI function; limiting instrumental ADL | Hospitalization indicated; elective operative intervention indicated; limiting self care ADL; disabling | Life-threatening consequences; urgent operative intervention indicated | Death |
| Definition: A disorder characterized by blockage of the normal flow of the intestinal contents in the jejunum. | | | | | |
| Jejunal perforation | - | Symptomatic; medical intervention indicated | Severe symptoms; elective operative intervention indicated | Life-threatening consequences; urgent operative intervention indicated | Death |
| Definition: A disorder characterized by a rupture in the jejunal wall. | | | | | |
| Jejunal stenosis | Asymptomatic; clinical or diagnostic observations only; intervention not indicated | Symptomatic; altered GI function | Severely altered GI function; tube feeding or hospitalization indicated; elective operative intervention indicated | Life-threatening consequences; urgent operative intervention indicated | Death |
| Definition: A disorder characterized by a narrowing of the lumen of the jejunum. | | | | | |

| **Gastrointestinal disorders** | | | | | |
| --- | --- | --- | --- | --- | --- |
|  | **Grade** | | | | |
| **Adverse Event** | **1** | **2** | **3** | **4** | **5** |
| Jejunal ulcer | Asymptomatic; clinical or diagnostic observations only; intervention not indicated | Symptomatic; altered GI function | Severely altered GI function; TPN indicated; elective operative or endoscopic intervention indicated; disabling | Life-threatening consequences; urgent operative intervention indicated | Death |
| Definition: A disorder characterized by a circumscribed, inflammatory and necrotic erosive lesion on the mucosal surface of the jejunum. | | | | | |
| Lip pain | Mild pain | Moderate pain; limiting instrumental ADL | Severe pain; limiting self care ADL | - | - |
| Definition: A disorder characterized by a sensation of marked discomfort of the lip. | | | | | |
| Lower gastrointestinal hemorrhage | Mild; intervention not indicated | Moderate symptoms; medical intervention or minor cauterization indicated | Transfusion, radiologic, endoscopic, or elective operative intervention indicated | Life-threatening consequences; urgent intervention indicated | Death |
| Definition: A disorder characterized by bleeding from the lower gastrointestinal tract (small intestine, large intestine, and anus). | | | | | |
| Malabsorption | - | Altered diet; oral intervention indicated | Inability to aliment adequately; TPN indicated | Life-threatening consequences; urgent intervention indicated | Death |
| Definition: A disorder characterized by inadequate absorption of nutrients in the small intestine. Symptoms include abdominal marked discomfort, bloating and diarrhea. | | | | | |
| Mucositis oral | Asymptomatic or mild symptoms; intervention not indicated | Moderate pain; not interfering with oral intake; modified diet indicated | Severe pain; interfering with oral intake | Life-threatening consequences; urgent intervention indicated | Death |
| Definition: A disorder characterized by inflammation of the oral mucosal. | | | | | |

| **Gastrointestinal disorders** | | | | | |
| --- | --- | --- | --- | --- | --- |
|  | **Grade** | | | | |
| **Adverse Event** | **1** | **2** | **3** | **4** | **5** |
| Nausea | Loss of appetite without alteration in eating habits | Oral intake decreased without significant weight loss, dehydration or malnutrition | Inadequate oral caloric or fluid intake; tube feeding, TPN, or hospitalization indicated | - | - |
| Definition: A disorder characterized by a queasy sensation and/or the urge to vomit. | | | | | |
| Obstruction gastric | Asymptomatic; clinical or diagnostic observations only; intervention not indicated | Symptomatic; altered GI function; limiting instrumental ADL | Hospitalization indicated; elective operative intervention indicated; limiting self care ADL; disabling | Life-threatening consequences; urgent operative intervention indicated | Death |
| Definition: A disorder characterized by blockage of the normal flow of the contents in the stomach. | | | | | |
| Oral cavity fistula | Asymptomatic; clinical or diagnostic observations only; intervention not indicated | Symptomatic; altered GI function | Severely altered GI function; TPN or hospitalization indicated; elective operative intervention indicated | Life-threatening consequences; urgent intervention indicated | Death |
| Definition: A disorder characterized by an abnormal communication between the oral cavity and another organ or anatomic site. | | | | | |
| Oral dysesthesia | Mild discomfort; not interfering with oral intake | Moderate pain; interfering with oral intake | Disabling pain; tube feeding or TPN indicated | - | - |
| Definition: A disorder characterized by a burning or tingling sensation on the lips, tongue or entire mouth. | | | | | |
| Oral hemorrhage | Mild; intervention not indicated | Moderate symptoms; medical intervention or minor cauterization indicated | Transfusion, radiologic, endoscopic, or elective operative intervention indicated | Life-threatening consequences; urgent intervention indicated | Death |
| Definition: A disorder characterized by bleeding from the mouth. | | | | | |

| **Gastrointestinal disorders** | | | | | |
| --- | --- | --- | --- | --- | --- |
|  | **Grade** | | | | |
| **Adverse Event** | **1** | **2** | **3** | **4** | **5** |
| Oral pain | Mild pain | Moderate pain; limiting instrumental ADL | Severe pain; limiting self care ADL | - | - |
| Definition: A disorder characterized by a sensation of marked discomfort in the mouth, tongue or lips. | | | | | |
| Pancreatic duct stenosis | Asymptomatic; clinical or diagnostic observations only; intervention not indicated | Symptomatic; altered GI function | Severely altered GI function; tube feeding or hospitalization indicated; elective operative intervention indicated | Life-threatening consequences; urgent operative intervention indicated | Death |
| Definition: A disorder characterized by a narrowing of the lumen of the pancreatic duct. | | | | | |
| Pancreatic fistula | Asymptomatic; clinical or diagnostic observations only; intervention not indicated | Symptomatic; altered GI function | Severely altered GI function; tube feeding or TPN or hospitalization indicated; elective operative intervention indicated | Life-threatening consequences; urgent operative intervention indicated | Death |
| Definition: A disorder characterized by an abnormal communication between the pancreas and another organ or anatomic site. | | | | | |
| Pancreatic hemorrhage | Mild; intervention not indicated | Moderate symptoms; medical intervention or minor cauterization indicated | Transfusion, radiologic, endoscopic, or elective operative intervention indicated | Life-threatening consequences; urgent intervention indicated | Death |
| Definition: A disorder characterized by bleeding from the pancreas. | | | | | |
| Pancreatic necrosis | - | - | Tube feeding or TPN indicated; radiologic, endoscopic, or operative intervention indicated | Life-threatening consequences; urgent operative intervention indicated | Death |

| **Gastrointestinal disorders** | | | | | |
| --- | --- | --- | --- | --- | --- |
|  | **Grade** | | | | |
| **Adverse Event** | **1** | **2** | **3** | **4** | **5** |
| Definition: A disorder characterized by a necrotic process occurring in the pancreas. | | | | | |
| Pancreatitis | - | Enzyme elevation or radiologic findings only | Severe pain; vomiting; medical intervention indicated (e.g., analgesia, nutritional support) | Life-threatening consequences; urgent intervention indicated | Death |
| Definition: A disorder characterized by inflammation of the pancreas. | | | | | |
| Periodontal disease | Gingival recession or gingivitis; limited bleeding on probing; mild local bone loss | Moderate gingival recession or gingivitis; multiple sites of bleeding on probing; moderate bone loss | Spontaneous bleeding; severe bone loss with or without tooth loss; osteonecrosis of maxilla or mandible | - | - |
| Definition: A disorder in the gingival tissue around the teeth. | | | | | |
| Peritoneal necrosis | - | - | Tube feeding or TPN indicated; radiologic, endoscopic, or operative intervention indicated | Life-threatening consequences; urgent operative intervention indicated | Death |
| Definition: A disorder characterized by a necrotic process occurring in the peritoneum. | | | | | |
| Proctitis | Rectal discomfort, intervention not indicated | Symptoms (e.g., rectal discomfort, passing blood or mucus); medical intervention indicated; limiting instrumental ADL | Severe symptoms; fecal urgency or stool incontinence; limiting self care ADL | Life-threatening consequences; urgent intervention indicated | Death |
| Definition: A disorder characterized by inflammation of the rectum. | | | | | |

| **Gastrointestinal disorders** | | | | | |
| --- | --- | --- | --- | --- | --- |
|  | **Grade** | | | | |
| **Adverse Event** | **1** | **2** | **3** | **4** | **5** |
| Rectal fistula | Asymptomatic; clinical or diagnostic observations only; intervention not indicated | Symptomatic; altered GI function | Severely altered GI function; TPN or hospitalization indicated; elective operative intervention indicated | Life-threatening consequences; urgent intervention indicated | Death |
| Definition: A disorder characterized by an abnormal communication between the rectum and another organ or anatomic site. | | | | | |
| Rectal hemorrhage | Mild; intervention not indicated | Moderate symptoms; medical intervention or minor cauterization indicated | Transfusion, radiologic, endoscopic, or elective operative intervention indicated | Life-threatening consequences; urgent intervention indicated | Death |
| Definition: A disorder characterized by bleeding from the rectal wall and discharged from the anus. | | | | | |
| Rectal mucositis | Asymptomatic or mild symptoms; intervention not indicated | Symptomatic; medical intervention indicated; limiting instrumental ADL | Severe symptoms; limiting self care ADL | Life-threatening consequences; urgent operative intervention indicated | Death |
| Definition: A disorder characterized by inflammation of the mucous membrane of the rectum. | | | | | |
| Rectal necrosis | - | - | Tube feeding or TPN indicated; radiologic, endoscopic, or operative intervention indicated | Life-threatening consequences; urgent operative intervention indicated | Death |
| Definition: A disorder characterized by a necrotic process occurring in the rectal wall. | | | | | |

| **Gastrointestinal disorders** | | | | | |
| --- | --- | --- | --- | --- | --- |
|  | **Grade** | | | | |
| **Adverse Event** | **1** | **2** | **3** | **4** | **5** |
| Rectal obstruction | Asymptomatic; clinical or diagnostic observations only; intervention not indicated | Symptomatic; altered GI function; limiting instrumental ADL | Hospitalization indicated; elective operative intervention indicated; limiting self care ADL; disabling | Life-threatening consequences; urgent operative intervention indicated | Death |
| Definition: A disorder characterized by blockage of the normal flow of the intestinal contents in the rectum. | | | | | |
| Rectal pain | Mild pain | Moderate pain; limiting instrumental ADL | Severe pain; limiting self care ADL | - | - |
| Definition: A disorder characterized by a sensation of marked discomfort in the rectal region. | | | | | |
| Rectal perforation | - | Symptomatic; medical intervention indicated | Severe symptoms; elective operative intervention indicated | Life-threatening consequences; urgent operative intervention indicated | Death |
| Definition: A disorder characterized by a rupture in the rectal wall. | | | | | |
| Rectal stenosis | Asymptomatic; clinical or diagnostic observations only; intervention not indicated | Symptomatic; altered GI function | Severely altered GI function; tube feeding or hospitalization indicated; elective operative intervention indicated | Life-threatening consequences; urgent operative intervention indicated | Death |
| Definition: A disorder characterized by a narrowing of the lumen of the rectum. | | | | | |
| Rectal ulcer | Asymptomatic; clinical or diagnostic observations only; intervention not indicated | Symptomatic; altered GI function (e.g. altered dietary habits, vomiting, diarrhea) | Severely altered GI function; TPN indicated; elective operative or endoscopic intervention indicated; disabling | Life-threatening consequences; urgent operative intervention indicated | Death |

| **Gastrointestinal disorders** | | | | | |
| --- | --- | --- | --- | --- | --- |
|  | **Grade** | | | | |
| **Adverse Event** | **1** | **2** | **3** | **4** | **5** |
| Definition: A disorder characterized by a circumscribed, inflammatory and necrotic erosive lesion on the mucosal surface of the rectum. | | | | | |
| Retroperitoneal hemorrhage | - | Self-limited; intervention indicated | Transfusion, medical, radiologic, endoscopic, or elective operative intervention indicated | Life-threatening consequences; urgent intervention indicated | Death |
| Definition: A disorder characterized by bleeding from the retroperitoneal area. | | | | | |
| Salivary duct inflammation | Slightly thickened saliva; slightly altered taste (e.g., metallic) | Thick, ropy, sticky saliva; markedly altered taste; alteration in diet indicated; secretion-induced symptoms; limiting instrumental ADL | Acute salivary gland necrosis; severe secretion-induced symptoms (e.g., thick saliva/oral secretions or gagging); tube feeding or TPN indicated; limiting self care ADL; disabling | Life-threatening consequences; urgent intervention indicated | Death |
| Definition: A disorder characterized by inflammation of the salivary duct. | | | | | |
| Salivary gland fistula | Asymptomatic; clinical or diagnostic observations only; intervention not indicated | Symptomatic; altered GI function; tube feeding indicated | Severely altered GI function; hospitalization indicated; elective operative intervention indicated | Life-threatening consequences; urgent operative intervention indicated | Death |
| Definition: A disorder characterized by an abnormal communication between a salivary gland and another organ or anatomic site. | | | | | |
| Small intestinal mucositis | Asymptomatic or mild symptoms; intervention not indicated | Symptomatic; medical intervention indicated; limiting instrumental ADL | Severe pain; interfering with oral intake; tube feeding, TPN or hospitalization indicated; limiting self care ADL | Life-threatening consequences; urgent intervention indicated | Death |

| **Gastrointestinal disorders** | | | | | |
| --- | --- | --- | --- | --- | --- |
|  | **Grade** | | | | |
| **Adverse Event** | **1** | **2** | **3** | **4** | **5** |
| Definition: A disorder characterized by inflammation of the mucous membrane of the small intestine. | | | | | |
| Small intestinal obstruction | Asymptomatic; clinical or diagnostic observations only; intervention not indicated | Symptomatic; altered GI function; limiting instrumental ADL | Hospitalization indicated; elective operative intervention indicated; limiting self care ADL; disabling | Life-threatening consequences; urgent operative intervention indicated | Death |
| Definition: A disorder characterized by blockage of the normal flow of the intestinal contents. | | | | | |
| Small intestinal perforation | - | Symptomatic; medical intervention indicated | Severe symptoms; elective operative intervention indicated | Life-threatening consequences; urgent operative intervention indicated | Death |
| Definition: A disorder characterized by a rupture in the small intestine wall. | | | | | |
| Small intestinal stenosis | Asymptomatic; clinical or diagnostic observations only; intervention not indicated | Symptomatic; altered GI function | Symptomatic and severely altered GI function; tube feeding, TPN or hospitalization indicated; non- emergent operative intervention indicated | Life-threatening consequences; urgent operative intervention indicated | Death |
| Definition: A disorder characterized by a narrowing of the lumen of the small intestine. | | | | | |
| Small intestine ulcer | Asymptomatic; clinical or diagnostic observations only; intervention not indicated | Symptomatic; altered GI function; limiting instrumental ADL | Severely altered GI function; TPN indicated; elective operative or endoscopic intervention indicated; limiting self care ADL; disabling | Life-threatening consequences; urgent operative intervention indicated | Death |

| **Gastrointestinal disorders** | | | | | |
| --- | --- | --- | --- | --- | --- |
|  | **Grade** | | | | |
| **Adverse Event** | **1** | **2** | **3** | **4** | **5** |
| Definition: A disorder characterized by a circumscribed, inflammatory and necrotic erosive lesion on the mucosal surface of the small intestine. | | | | | |
| Stomach pain | Mild pain | Moderate pain; limiting instrumental ADL | Severe pain; limiting self care ADL | - | - |
| Definition: A disorder characterized by a sensation of marked discomfort in the stomach. | | | | | |
| Tooth development disorder | Asymptomatic; hypoplasia of tooth or enamel | Impairment correctable with oral surgery | Maldevelopment with impairment not surgically correctable; disabling | - | - |
| Definition: A disorder characterized by a pathological process of the teeth occurring during tooth development. | | | | | |
| Tooth discoloration | Surface stains | - | - | - | - |
| Definition: A disorder characterized by a change in tooth hue or tint. | | | | | |
| Toothache | Mild pain | Moderate pain; limiting instrumental ADL | Severe pain; limiting self care ADL | - | - |
| Definition: A disorder characterized by a sensation of marked discomfort in the tooth. | | | | | |
| Typhlitis | - | - | Symptomatic (e.g., abdominal pain, fever, change in bowel habits with ileus); peritoneal signs | Life-threatening consequences; urgent operative intervention indicated | Death |
| Definition: A disorder characterized by inflammation of the cecum. | | | | | |
| Upper gastrointestinal hemorrhage | Mild; intervention not indicated | Moderate symptoms; medical intervention or minor cauterization indicated | Transfusion, radiologic, endoscopic, or elective operative intervention indicated | Life-threatening consequences; urgent intervention indicated | Death |

| **Gastrointestinal disorders** | | | | | |
| --- | --- | --- | --- | --- | --- |
|  | **Grade** | | | | |
| **Adverse Event** | **1** | **2** | **3** | **4** | **5** |
| Definition: A disorder characterized by bleeding from the upper gastrointestinal tract (oral cavity, pharynx, esophagus, and stomach). | | | | | |
| Vomiting | 1 - 2 episodes (separated by 5 minutes) in 24 hrs | 3 - 5 episodes (separated by 5 minutes) in 24 hrs | >=6 episodes (separated by 5 minutes) in 24 hrs; tube feeding, TPN or hospitalization indicated | Life-threatening consequences; urgent intervention indicated | Death |
| Definition: A disorder characterized by the reflexive act of ejecting the contents of the stomach through the mouth. | | | | | |
| Gastrointestinal disorders - Other, specify | Asymptomatic or mild symptoms; clinical or diagnostic observations only; intervention not indicated | Moderate; minimal, local or noninvasive intervention indicated; limiting age- appropriate instrumental ADL | Severe or medically significant but not immediately life- threatening; hospitalization or prolongation of existing hospitalization indicated; disabling; limiting self care ADL | Life-threatening consequences; urgent intervention indicated | Death |

| **Cardiac disorders** | | | | | |
| --- | --- | --- | --- | --- | --- |
|  | **Grade** | | | | |
| **Adverse Event** | **1** | **2** | **3** | **4** | **5** |
| Acute coronary syndrome | - | Symptomatic, progressive angina; cardiac enzymes normal; hemodynamically stable | Symptomatic, unstable angina and/or acute myocardial infarction, cardiac enzymes abnormal, hemodynamically stable | Symptomatic, unstable angina and/or acute myocardial infarction, cardiac enzymes abnormal, hemodynamically unstable | Death |
| Definition: A disorder characterized by signs and symptoms related to acute ischemia of the myocardium secondary to coronary artery disease. The clinical presentation covers a spectrum of heart diseases from unstable angina to myocardial infarction. | | | | | |
| Aortic valve disease | Asymptomatic valvular thickening with or without mild valvular regurgitation or stenosis by imaging | Asymptomatic; moderate regurgitation or stenosis by imaging | Symptomatic; severe regurgitation or stenosis by imaging; symptoms controlled with medical intervention | Life-threatening consequences; urgent intervention indicated (e.g., valve replacement, valvuloplasty) | Death |
| Definition: A disorder characterized by a defect in aortic valve function or structure. | | | | | |
| Asystole | Periods of asystole; non- urgent medical management indicated | - | - | Life-threatening consequences; urgent intervention indicated | Death |
| Definition: A disorder characterized by a dysrhythmia without cardiac electrical activity. Typically, this is accompanied by cessation of the pumping function of the heart. | | | | | |
| Atrial fibrillation | Asymptomatic, intervention not indicated | Non-urgent medical intervention indicated | Symptomatic and incompletely controlled medically, or controlled with device (e.g., pacemaker), or ablation | Life-threatening consequences; urgent intervention indicated | Death |

| **Cardiac disorders** | | | | | |
| --- | --- | --- | --- | --- | --- |
|  | **Grade** | | | | |
| **Adverse Event** | **1** | **2** | **3** | **4** | **5** |
| Definition: A disorder characterized by a dysrhythmia without discernible P waves and an irregular ventricular response due to multiple reentry circuits. The rhythm disturbance originates above the ventricles. | | | | | |
| Atrial flutter | Asymptomatic, intervention not indicated | Non-urgent medical intervention indicated | Symptomatic and incompletely controlled medically, or controlled with device (e.g., pacemaker), or ablation | Life-threatening consequences; urgent intervention indicated | Death |
| Definition: A disorder characterized by a dysrhythmia with organized rhythmic atrial contractions with a rate of 200-300 beats per minute. The rhythm disturbance originates in the atria. | | | | | |
| Atrioventricular block complete | - | Non-urgent intervention indicated | Symptomatic and incompletely controlled medically, or controlled with device (e.g., pacemaker) | Life-threatening consequences; urgent intervention indicated | Death |
| Definition: A disorder characterized by a dysrhythmia with complete failure of atrial electrical impulse conduction through the AV node to the ventricles. | | | | | |
| Atrioventricular block first degree | Asymptomatic, intervention not indicated | Non-urgent intervention indicated | - | - | - |
| Definition: A disorder characterized by a dysrhythmia with a delay in the time required for the conduction of an electrical impulse through the atrioventricular (AV) node beyond 0.2 seconds; prolongation of the PR interval greater than 200 milliseconds. | | | | | |
| Cardiac arrest | - | - | - | Life-threatening consequences; urgent intervention indicated | Death |
| Definition: A disorder characterized by cessation of the pumping function of the heart. | | | | | |

| **Cardiac disorders** | | | | | |
| --- | --- | --- | --- | --- | --- |
|  | **Grade** | | | | |
| **Adverse Event** | **1** | **2** | **3** | **4** | **5** |
| Chest pain - cardiac | Mild pain | Moderate pain; limiting instrumental ADL | Pain at rest; limiting self care ADL | - | - |
| Definition: A disorder characterized by substernal discomfort due to insufficient myocardial oxygenation. | | | | | |
| Conduction disorder | Mild symptoms; intervention not indicated | Moderate symptoms | Severe symptoms; intervention indicated | Life-threatening consequences; urgent intervention indicated | Death |
| Definition: A disorder characterized by pathological irregularities in the cardiac conduction system. | | | | | |
| Constrictive pericarditis | - | - | Symptomatic heart failure or other cardiac symptoms, responsive to intervention | Refractory heart failure or other poorly controlled cardiac symptoms | Death |
| Definition: A disorder characterized by a thickened and fibrotic pericardial sac; these fibrotic changes impede normal myocardial function by restricting myocardial muscle action. | | | | | |
| Heart failure | Asymptomatic with laboratory (e.g., BNP [B-Natriuretic Peptide ]) or cardiac imaging abnormalities | Symptoms with mild to moderate activity or exertion | Severe with symptoms at rest or with minimal activity or exertion; intervention indicated | Life-threatening consequences; urgent intervention indicated (e.g., continuous IV therapy or mechanical hemodynamic support) | Death |
| Definition: A disorder characterized by the inability of the heart to pump blood at an adequate volume to meet tissue metabolic requirements, or, the ability to do so only at an elevation in the filling pressure. | | | | | |

| **Cardiac disorders** | | | | | |
| --- | --- | --- | --- | --- | --- |
|  | **Grade** | | | | |
| **Adverse Event** | **1** | **2** | **3** | **4** | **5** |
| Left ventricular systolic dysfunction | - | - | Symptomatic due to drop in ejection fraction responsive to intervention | Refractory or poorly controlled heart failure due to drop in ejection fraction; intervention such as ventricular assist device, intravenous vasopressor support, or heart transplant indicated | Death |
| Definition: A disorder characterized by failure of the left ventricle to produce adequate output despite an increase in distending pressure and in end-diastolic volume. Clinical manifestations mayinclude dyspnea, orthopnea, and other signs and symptoms of pulmonary congestion and edema. | | | | | |
| Mitral valve disease | Asymptomatic valvular thickening with or without mild valvular regurgitation or stenosis by imaging | Asymptomatic; moderate regurgitation or stenosis by imaging | Symptomatic; severe regurgitation or stenosis by imaging; symptoms controlled with medical intervention | Life-threatening consequences; urgent intervention indicated (e.g., valve replacement, valvuloplasty) | Death |
| Definition: A disorder characterized by a defect in mitral valve function or structure. | | | | | |
| Mobitz (type) II atrioventricular block | Asymptomatic, intervention not indicated | Symptomatic; medical intervention indicated | Symptomatic and incompletely controlled medically, or controlled with device (e.g., pacemaker) | Life-threatening consequences; urgent intervention indicated | Death |
| Definition: A disorder characterized by a dysrhythmia with relatively constant PR interval prior to the block of an atrial impulse. This is the result of intermittent failure of atrial electrical impulse conduction through the atrioventricular (AV) node to the ventricles. | | | | | |

| **Cardiac disorders** | | | | | |
| --- | --- | --- | --- | --- | --- |
|  | **Grade** | | | | |
| **Adverse Event** | **1** | **2** | **3** | **4** | **5** |
| Mobitz type I | Asymptomatic, intervention not indicated | Symptomatic; medical intervention indicated | Symptomatic and incompletely controlled medically, or controlled with device (e.g., pacemaker) | Life-threatening consequences; urgent intervention indicated | Death |
| Definition: A disorder characterized by a dysrhythmia with a progressively lengthening PR interval prior to the blocking of an atrial impulse. This is the result of intermittent failure of atrial electrical impulse conduction through the atrioventricular (AV) node to the ventricles. | | | | | |
| Myocardial infarction | - | Asymptomatic and cardiac enzymes minimally abnormal and no evidence of ischemic ECG changes | Severe symptoms; cardiac enzymes abnormal; hemodynamically stable; ECG changes consistent with infarction | Life-threatening consequences; hemodynamically unstable | Death |
| Definition: A disorder characterized by gross necrosis of the myocardium; this is due to an interruption of blood supply to the area. | | | | | |
| Myocarditis | Asymptomatic with laboratory (e.g., BNP [B-Natriuretic Peptide ]) or cardiac imaging abnormalities | Symptoms with mild to moderate activity or exertion | Severe with symptoms at rest or with minimal activity or exertion; intervention indicated | Life-threatening consequences; urgent intervention indicated (e.g., continuous IV therapy or mechanical hemodynamic support) | Death |
| Definition: A disorder characterized by inflammation of the muscle tissue of the heart. | | | | | |
| Palpitations | Mild symptoms; intervention not indicated | Intervention indicated | - | - | - |
| Definition: A disorder characterized by an unpleasant sensation of irregular and/or forceful beating of the heart. | | | | | |

| **Cardiac disorders** | | | | | |
| --- | --- | --- | --- | --- | --- |
|  | **Grade** | | | | |
| **Adverse Event** | **1** | **2** | **3** | **4** | **5** |
| Paroxysmal atrial tachycardia | Asymptomatic, intervention not indicated | Symptomatic; medical management indicated | IV medication indicated | Life-threatening consequences; incompletely controlled medically; cardioversion indicated | Death |
| Definition: A disorder characterized by a dysrhythmia with abrupt onset and sudden termination of atrial contractions with a rate of 150-250 beats per minute. The rhythm disturbance originates in the atria. | | | | | |
| Pericardial effusion | - | Asymptomatic effusion size small to moderate | Effusion with physiologic consequences | Life-threatening consequences; urgent intervention indicated | Death |
| Definition: A disorder characterized by fluid collection within the pericardial sac, usually due to inflammation. | | | | | |
| Pericardial tamponade | - | - | - | Life-threatening consequences; urgent intervention indicated | Death |
| Definition: A disorder characterized by an increase in intrapericardial pressure due to the collection of blood or fluid in the pericardium. | | | | | |
| Pericarditis | Asymptomatic, ECG or physical findings (e.g., rub) consistent with pericarditis | Symptomatic pericarditis (e.g., chest pain) | Pericarditis with physiologic consequences (e.g., pericardial constriction) | Life-threatening consequences; urgent intervention indicated | Death |
| Definition: A disorder characterized by irritation to the layers of the pericardium (the protective sac around the heart). | | | | | |
| Pulmonary valve disease | Asymptomatic valvular thickening with or without mild valvular regurgitation or stenosis by imaging | Asymptomatic; moderate regurgitation or stenosis by imaging | Symptomatic; severe regurgitation or stenosis by imaging; symptoms controlled with medical intervention | Life-threatening consequences; urgent intervention indicated (e.g., valve replacement, valvuloplasty) | Death |

| **Cardiac disorders** | | | | | |
| --- | --- | --- | --- | --- | --- |
|  | **Grade** | | | | |
| **Adverse Event** | **1** | **2** | **3** | **4** | **5** |
| Definition: A disorder characterized by a defect in pulmonary valve function or structure. | | | | | |
| Restrictive cardiomyopathy | - | - | Symptomatic heart failure or other cardiac symptoms, responsive to intervention | Refractory heart failure or other poorly controlled cardiac symptoms | Death |
| Definition: A disorder characterized by an inability of the ventricles to fill with blood because the myocardium (heart muscle) stiffens and loses its flexibility. | | | | | |
| Right ventricular dysfunction | Asymptomatic with laboratory (e.g., BNP [B-Natriuretic Peptide ]) or cardiac imaging abnormalities | Symptoms with mild to moderate activity or exertion | Severe symptoms, associated with hypoxemia, right heart failure; oxygen indicated | Life-threatening consequences; urgent intervention indicated (e.g., ventricular assist device); heart transplant indicated | Death |
| Definition: A disorder characterized by impairment of right ventricular function associated with low ejection fraction and a decrease in motility of the right ventricular wall. | | | | | |
| Sick sinus syndrome | Asymptomatic, intervention not indicated | Non-urgent intervention indicated | Severe, medically significant; medical intervention indicated | Life-threatening consequences; urgent intervention indicated | Death |
| Definition: A disorder characterized by a dysrhythmia with alternating periods of bradycardia and atrial tachycardia accompanied by syncope, fatigue and dizziness. | | | | | |
| Sinus bradycardia | Asymptomatic, intervention not indicated | Symptomatic, medical intervention indicated | Severe, medically significant, medical intervention indicated | Life-threatening consequences; urgent intervention indicated | Death |
| Definition: A disorder characterized by a dysrhythmia with a heart rate less than 60 beats per minute that originates in the sinus node. | | | | | |
| Sinus tachycardia | Asymptomatic, intervention not indicated | Symptomatic; non-urgent medical intervention indicated | Urgent medical intervention indicated | - | - |
| Definition: A disorder characterized by a dysrhythmia with a heart rate greater than 100 beats per minute that originates in the sinus node. | | | | | |

| **Cardiac disorders** | | | | | |
| --- | --- | --- | --- | --- | --- |
|  | **Grade** | | | | |
| **Adverse Event** | **1** | **2** | **3** | **4** | **5** |
| Supraventricular tachycardia | Asymptomatic, intervention not indicated | Non-urgent medical intervention indicated | Medical intervention indicated | Life-threatening consequences; urgent intervention indicated | Death |
| Definition: A disorder characterized by a dysrhythmia with a heart rate greater than 100 beats per minute that originates above the ventricles. | | | | | |
| Tricuspid valve disease | Asymptomatic valvular thickening with or without mild valvular regurgitation or stenosis | Asymptomatic; moderate regurgitation or stenosis by imaging | Symptomatic; severe regurgitation or stenosis; symptoms controlled with medical intervention | Life-threatening consequences; urgent intervention indicated (e.g., valve replacement, valvuloplasty) | Death |
| Definition: A disorder characterized by a defect in tricuspid valve function or structure. | | | | | |
| Ventricular arrhythmia | Asymptomatic, intervention not indicated | Non-urgent medical intervention indicated | Medical intervention indicated | Life-threatening consequences; hemodynamic compromise; urgent intervention indicated | Death |
| Definition: A disorder characterized by a dysrhythmia that originates in the ventricles. | | | | | |
| Ventricular fibrillation | - | - | - | Life-threatening consequences; hemodynamic compromise; urgent intervention indicated | Death |
| Definition: A disorder characterized by a dysrhythmia without discernible QRS complexes due to rapid repetitive excitation of myocardial fibers without coordinated contraction of the ventricles. | | | | | |

| **Cardiac disorders** | | | | | |
| --- | --- | --- | --- | --- | --- |
|  | **Grade** | | | | |
| **Adverse Event** | **1** | **2** | **3** | **4** | **5** |
| Ventricular tachycardia | - | Non-urgent medical intervention indicated | Medical intervention indicated | Life-threatening consequences; hemodynamic compromise; urgent intervention indicated | Death |
| Definition: A disorder characterized by a dysrhythmia with a heart rate greater than 100 beats per minute that originates distal to the bundle of His. | | | | | |
| Wolff-Parkinson-White syndrome | Asymptomatic, intervention not indicated | Non-urgent medical intervention indicated | Symptomatic and incompletely controlled medically or controlled with procedure | Life-threatening consequences; urgent intervention indicated | Death |
| Definition: A disorder characterized by the presence of an accessory conductive pathway between the atria and the ventricles that causes premature ventricular activation. | | | | | |
| Cardiac disorders - Other, specify | Asymptomatic or mild symptoms; clinical or diagnostic observations only; intervention not indicated | Moderate; minimal, local or noninvasive intervention indicated; limiting age- appropriate instrumental ADL | Severe or medically significant but not immediately life- threatening; hospitalization or prolongation of existing hospitalization indicated; disabling; limiting self care ADL | Life-threatening consequences; urgent intervention indicated | Death |

| **Vascular disorders** | | | | | |
| --- | --- | --- | --- | --- | --- |
|  | **Grade** | | | | |
| **Adverse Event** | **1** | **2** | **3** | **4** | **5** |
| Capillary leak syndrome | - | Symptomatic; medical intervention indicated | Severe symptoms; intervention indicated | Life-threatening consequences; urgent intervention indicated | Death |
| Definition: A disorder characterized by leakage of intravascular fluids into the extravascular space. This syndrome is observed in patients who demonstrate a state of generalized leaky capillaries following shock syndromes, low-flow states, ischemia-reperfusion injuries, toxemias, medications, or poisoning. It can lead to generalized edema and multiple organ failure. | | | | | |
| Flushing | Asymptomatic; clinical or diagnostic observations only; intervention not indicated | Moderate symptoms; medical intervention indicated; limiting instrumental ADL | Symptomatic, associated with hypotension and/or tachycardia; limiting self care ADL | - | - |
| Definition: A disorder characterized by episodic reddening of the face. | | | | | |
| Hematoma | Mild symptoms; intervention not indicated | Minimally invasive evacuation or aspiration indicated | Transfusion, radiologic, endoscopic, or elective operative intervention indicated | Life-threatening consequences; urgent intervention indicated | Death |
| Definition: A disorder characterized by a localized collection of blood, usually clotted, in an organ, space, or tissue, due to a break in the wall of a blood vessel. | | | | | |
| Hot flashes | Mild symptoms; intervention not indicated | Moderate symptoms; limiting instrumental ADL | Severe symptoms; limiting self care ADL | - | - |
| Definition: A disorder characterized by an uncomfortable and temporary sensation of intense body warmth, flushing, sometimes accompanied by sweating upon cooling. | | | | | |

| **Vascular disorders** | | | | | |
| --- | --- | --- | --- | --- | --- |
|  | **Grade** | | | | |
| **Adverse Event** | **1** | **2** | **3** | **4** | **5** |
| Hypertension | Prehypertension (systolic BP 120 - 139 mm Hg or diastolic BP 80 - 89 mm Hg) | Stage 1 hypertension (systolic BP 140 - 159 mm Hg or diastolic BP 90 - 99 mm Hg); medical intervention indicated; recurrent or persistent (>=24 hrs); symptomatic increase by  >20 mm Hg (diastolic) or to  >140/90 mm Hg if previously WNL; monotherapy indicated Pediatric: recurrent or persistent (>=24 hrs) BP  >ULN; monotherapy indicated | Stage 2 hypertension (systolic BP >=160 mm Hg or diastolic BP >=100 mm Hg); medical intervention indicated; more than one drug or more intensive therapy than previously used indicated Pediatric: Same as adult | Life-threatening consequences (e.g., malignant hypertension, transient or permanent neurologic deficit, hypertensive crisis); urgent intervention indicated Pediatric: Same as adult | Death |
| Definition: A disorder characterized by a pathological increase in blood pressure; a repeatedly elevation in the blood pressure exceeding 140 over 90 mm Hg. | | | | | |
| Hypotension | Asymptomatic, intervention not indicated | Non-urgent medical intervention indicated | Medical intervention or hospitalization indicated | Life-threatening and urgent intervention indicated | Death |
| Definition: A disorder characterized by a blood pressure that is below the normal expected for an individual in a given environment. | | | | | |
| Lymph leakage | - | Symptomatic; medical intervention indicated | Severe symptoms; radiologic, endoscopic or elective operative intervention indicated | Life-threatening consequences; urgent intervention indicated | Death |
| Definition: A disorder characterized by the loss of lymph fluid into the surrounding tissue or body cavity. | | | | | |

| **Vascular disorders** | | | | | |
| --- | --- | --- | --- | --- | --- |
|  | **Grade** | | | | |
| **Adverse Event** | **1** | **2** | **3** | **4** | **5** |
| Lymphedema | Trace thickening or faint discoloration | Marked discoloration; leathery skin texture; papillary formation; limiting instrumental ADL | Severe symptoms; limiting self care ADL | - | - |
| Definition: A disorder characterized by excessive fluid collection in tissues that causes swelling. | | | | | |
| Lymphocele | Asymptomatic; clinical or diagnostic observations only; intervention not indicated | Symptomatic; medical intervention indicated | Severe symptoms; radiologic, endoscopic or elective operative intervention indicated | - | - |
| Definition: A disorder characterized by a cystic lesion containing lymph. | | | | | |
| Peripheral ischemia | - | Brief (<24 hrs) episode of ischemia managed non- surgically and without permanent deficit | Recurring or prolonged (>=24 hrs) and/or invasive intervention indicated | Life-threatening consequences; evidence of end organ damage; urgent operative intervention indicated | Death |
| Definition: A disorder characterized by impaired circulation to an extremity. | | | | | |
| Phlebitis | - | Present | - | - | - |
| Definition: A disorder characterized by inflammation of the wall of a vein. | | | | | |
| Superficial thrombophlebitis | - | Present | - | - | - |
| Definition: A disorder characterized by a blood clot and inflammation involving a superficial vein of the extremities. | | | | | |

| **Vascular disorders** | | | | | |
| --- | --- | --- | --- | --- | --- |
|  | **Grade** | | | | |
| **Adverse Event** | **1** | **2** | **3** | **4** | **5** |
| Superior vena cava syndrome | Asymptomatic; incidental finding of SVC thrombosis | Symptomatic; medical intervention indicated (e.g., anticoagulation, radiation or chemotherapy) | Severe symptoms; multi- modality intervention indicated (e.g., anticoagulation, chemotherapy, radiation, stenting) | Life-threatening consequences; urgent multi- modality intervention indicated (e.g., lysis, thrombectomy, surgery) | Death |
| Definition: A disorder characterized by obstruction of the blood flow in the superior vena cava. Signs and symptoms include swelling and cyanosis of the face, neck, and upper arms, cough, orthopnea and headache. | | | | | |
| Thromboembolic event | Venous thrombosis (e.g., superficial thrombosis) | Venous thrombosis (e.g., uncomplicated deep vein thrombosis), medical intervention indicated | Thrombosis (e.g., uncomplicated pulmonary embolism [venous], non- embolic cardiac mural [arterial] thrombus), medical intervention indicated | Life-threatening (e.g., pulmonary embolism, cerebrovascular event, arterial insufficiency); hemodynamic or neurologic instability; urgent intervention indicated | Death |
| Definition: A disorder characterized by occlusion of a vessel by a thrombus that has migrated from a distal site via the blood stream. | | | | | |
| Vasculitis | Asymptomatic, intervention not indicated | Moderate symptoms, medical intervention indicated | Severe symptoms, medical intervention indicated (e.g., steroids) | Life-threatening; evidence of peripheral or visceral ischemia; urgent intervention indicated | Death |
| Definition: A disorder characterized by inflammation involving the wall of a vessel. | | | | | |
| Visceral arterial ischemia | - | Brief (<24 hrs) episode of ischemia managed medically and without permanent deficit | Prolonged (>=24 hrs) or recurring symptoms and/or invasive intervention indicated | Life-threatening consequences; evidence of end organ damage; urgent operative intervention indicated | Death |

| **Vascular disorders** | | | | | |
| --- | --- | --- | --- | --- | --- |
|  | **Grade** | | | | |
| **Adverse Event** | **1** | **2** | **3** | **4** | **5** |
| Definition: A disorder characterized by a decrease in blood supply due to narrowing or blockage of a visceral (mesenteric) artery. | | | | | |
| Vascular disorders - Other, specify | Asymptomatic or mild symptoms; clinical or diagnostic observations only; intervention not indicated | Moderate; minimal, local or noninvasive intervention indicated; limiting age- appropriate instrumental ADL | Severe or medically significant but not immediately life- threatening; hospitalization or prolongation of existing hospitalization indicated; disabling; limiting self care ADL | Life-threatening consequences; urgent intervention indicated | Death |

| **Skin and subcutaneous tissue disorders** | | | | | |
| --- | --- | --- | --- | --- | --- |
|  | **Grade** | | | | |
| **Adverse Event** | **1** | **2** | **3** | **4** | **5** |
| Alopecia | Hair loss of <50% of normal for that individual that is not obvious from a distance but only on close inspection; a different hair style may be required to cover the hair loss but it does not require a wig or hair piece to camouflage | Hair loss of >=50% normal for that individual that is readily apparent to others; a wig or hair piece is necessary if the patient desires to completely camouflage the hair loss; associated with psychosocial impact | - | - | - |
| Definition: A disorder characterized by a decrease in density of hair compared to normal for a given individual at a given age and body location. | | | | | |
| Body odor | Mild odor; physician intervention not indicated; self care interventions | Pronounced odor; psychosocial impact; patient seeks medical intervention | - | - | - |
| Definition: A disorder characterized by an abnormal body smell resulting from the growth of bacteria on the body. | | | | | |
| Bullous dermatitis | Asymptomatic; blisters covering <10% BSA | Blisters covering 10 - 30% BSA; painful blisters; limiting instrumental ADL | Blisters covering >30% BSA; limiting self care ADL | Blisters covering >30% BSA; associated with fluid or electrolyte abnormalities; ICU care or burn unit indicated | Death |
| Definition: A disorder characterized by inflammation of the skin characterized by the presence of bullae which are filled with fluid. | | | | | |
| Dry skin | Covering <10% BSA and no associated erythema or pruritus | Covering 10 - 30% BSA and associated with erythema or pruritus; limiting instrumental ADL | Covering >30% BSA and associated with pruritus; limiting self care ADL | - | - |
| Definition: A disorder characterized by flaky and dull skin; the pores are generally fine, the texture is a papery thin texture. | | | | | |

| **Skin and subcutaneous tissue disorders** | | | | | |
| --- | --- | --- | --- | --- | --- |
|  | **Grade** | | | | |
| **Adverse Event** | **1** | **2** | **3** | **4** | **5** |
| Erythema multiforme | Target lesions covering <10% BSA and not associated with skin tenderness | Target lesions covering 10 - 30% BSA and associated with skin tenderness | Target lesions covering >30% BSA and associated with oral or genital erosions | Target lesions covering >30% BSA; associated with fluid or electrolyte abnormalities; ICU care or burn unit indicated | Death |
| Definition: A disorder characterized by target lesions (a pink-red ring around a pale center). | | | | | |
| Erythroderma | - | Erythema covering >90% BSA without associated symptoms; limiting instrumental ADL | Erythema covering >90% BSA with associated symptoms (e.g., pruritus or tenderness); limiting self care ADL | Erythema covering >90% BSA with associated fluid or electrolyte abnormalities; ICU care or burn unit indicated | Death |
| Definition: A disorder characterized by generalized inflammatory erythema and exfoliation. The inflammatory process involves > 90% of the body surface area. | | | | | |
| Fat atrophy | Covering <10% BSA and asymptomatic | Covering 10 - 30% BSA and associated with erythema or tenderness; limiting instrumental ADL | Covering >30% BSA; associated with erythema or tenderness; limiting self-care ADL | - | - |
| Definition: A disorder characterized by shrinking of adipose tissue. | | | | | |
| Hirsutism | In women, increase in length, thickness or density of hair in a male distribution that the patient is able to camouflage by periodic shaving, bleaching, or removal of hair | In women, increase in length, thickness or density of hair in a male distribution that requires daily shaving or consistent destructive means of hair removal to camouflage; associated with psychosocial impact | - | - | - |

| **Skin and subcutaneous tissue disorders** | | | | | |
| --- | --- | --- | --- | --- | --- |
|  | **Grade** | | | | |
| **Adverse Event** | **1** | **2** | **3** | **4** | **5** |
| Definition: A disorder characterized by the presence of excess hair growth in women in anatomic sites where growth is considered to be a secondary male characteristic and under androgen control (beard, moustache, chest, abdomen) | | | | | |
| Hyperhidrosis | Limited to one site (palms, soles, or axillae); self care interventions | Involving >1 site; patient seeks medical intervention; associated with psychosocial impact | Generalized involving sites other than palms, soles, or axillae; associated with electrolyte/hemodynamic imbalance | - | - |
| Definition: A disorder characterized by excessive perspiration. | | | | | |
| Hypertrichosis | Increase in length, thickness or density of hair that the patient is either able to camouflage by periodic shaving or removal of hairs or is not concerned enough about the overgrowth to use any form of hair removal | Increase in length, thickness or density of hair at least on the usual exposed areas of the body [face (not limited to beard/moustache area) plus/minus arms] that requires frequent shaving or use of destructive means of hair removal to camouflage; associated with psychosocial impact | - | - | - |
| Definition: A disorder characterized by hair density or length beyond the accepted limits of normal in a particular body region, for a particular age or race. | | | | | |
| Hypohidrosis | - | Symptomatic; limiting instrumental ADL | Increase in body temperature; limiting self care ADL | Heat stroke | Death |
| Definition: A disorder characterized by reduced sweating. | | | | | |

| **Skin and subcutaneous tissue disorders** | | | | | |
| --- | --- | --- | --- | --- | --- |
|  | **Grade** | | | | |
| **Adverse Event** | **1** | **2** | **3** | **4** | **5** |
| Lipohypertrophy | Asymptomatic and covering  <10% BSA | Covering 10 - 30% BSA and associated tenderness; limiting instrumental ADL | Covering >30% BSA and associated tenderness and narcotics or NSAIDs indicated; lipohypertrophy; limiting self care ADL | - | - |
| Definition: A disorder characterized by hypertrophy of the subcutaneous adipose tissue at the site of multiple subcutaneous injections of insulin. | | | | | |
| Nail discoloration | Asymptomatic; clinical or diagnostic observations only; intervention not indicated | - | - | - | - |
| Definition: A disorder characterized by a change in the color of the nail plate. | | | | | |
| Nail loss | Asymptomatic separation of the nail bed from the nail plate or nail loss | Symptomatic separation of the nail bed from the nail plate or nail loss; limiting instrumental ADL | - | - | - |
| Definition: A disorder characterized by loss of all or a portion of the nail. | | | | | |
| Nail ridging | Asymptomatic; clinical or diagnostic observations only; intervention not indicated | - | - | - | - |
| Definition: A disorder characterized by vertical or horizontal ridges on the nails. | | | | | |
| Pain of skin | Mild pain | Moderate pain; limiting instrumental ADL | Severe pain; limiting self care ADL | - | - |
| Definition: A disorder characterized by marked discomfort sensation in the skin. | | | | | |

| **Skin and subcutaneous tissue disorders** | | | | | |
| --- | --- | --- | --- | --- | --- |
|  | **Grade** | | | | |
| **Adverse Event** | **1** | **2** | **3** | **4** | **5** |
| Palmar-plantar erythrodysesthesia syndrome | Minimal skin changes or dermatitis (e.g., erythema, edema, or hyperkeratosis) without pain | Skin changes (e.g., peeling, blisters, bleeding, edema, or hyperkeratosis) with pain; limiting instrumental ADL | Severe skin changes (e.g., peeling, blisters, bleeding, edema, or hyperkeratosis) with pain; limiting self care ADL | - | - |
| Definition: A disorder characterized by redness, marked discomfort, swelling, and tingling in the palms of the hands or the soles of the feet. | | | | | |
| Periorbital edema | Soft or non-pitting | Indurated or pitting edema; topical intervention indicated | Edema associated with visual disturbance; increased intraocular pressure, glaucoma or retinal hemorrhage; optic neuritis; diuretics indicated; operative intervention indicated | - | - |
| Definition: A disorder characterized by swelling due to an excessive accumulation of fluid around the orbits of the face. | | | | | |
| Photosensitivity | Painless erythema and erythema covering <10% BSA | Tender erythema covering 10  - 30% BSA | Erythema covering >30% BSA and erythema with blistering; photosensitivity; oral corticosteroid therapy indicated; pain control indicated (e.g., narcotics or NSAIDs) | Life-threatening consequences; urgent intervention indicated | Death |
| Definition: A disorder characterized by an increase in sensitivity of the skin to light. | | | | | |

| **Skin and subcutaneous tissue disorders** | | | | | |
| --- | --- | --- | --- | --- | --- |
|  | **Grade** | | | | |
| **Adverse Event** | **1** | **2** | **3** | **4** | **5** |
| Pruritus | Mild or localized; topical intervention indicated | Intense or widespread; intermittent; skin changes from scratching (e.g., edema, papulation, excoriations, lichenification, oozing/crusts); oral intervention indicated; limiting instrumental ADL | Intense or widespread; constant; limiting self care ADL or sleep; oral corticosteroid or immunosuppressive therapy indicated | - | - |
| Definition: A disorder characterized by an intense itching sensation. | | | | | |
| Purpura | Combined area of lesions covering <10% BSA | Combined area of lesions covering 10 - 30% BSA; bleeding with trauma | Combined area of lesions covering >30% BSA; spontaneous bleeding | - | - |
| Definition: A disorder characterized by hemorrhagic areas of the skin and mucous membrane. Newer lesions appear reddish in color. Older lesions are usually a darker purple color and eventually become a brownish-yellow color. | | | | | |
| Rash acneiform | Papules and/or pustules covering <10% BSA, which may or may not be associated with symptoms of pruritus or tenderness | Papules and/or pustules covering 10 - 30% BSA, which may or may not be associated with symptoms of pruritus or tenderness; associated with psychosocial impact; limiting instrumental ADL | Papules and/or pustules covering >30% BSA, which may or may not be associated with symptoms of pruritus or tenderness; limiting self care ADL; associated with local superinfection with oral antibiotics indicated | Papules and/or pustules covering any % BSA, which may or may not be associated with symptoms of pruritus or tenderness and are associated with extensive superinfection with IV antibiotics indicated; life- threatening consequences | Death |
| Definition: A disorder characterized by an eruption of papules and pustules, typically appearing in face, scalp, upper chest and back. | | | | | |

| **Skin and subcutaneous tissue disorders** | | | | | |
| --- | --- | --- | --- | --- | --- |
|  | **Grade** | | | | |
| **Adverse Event** | **1** | **2** | **3** | **4** | **5** |
| Rash maculo-papular | Macules/papules covering  <10% BSA with or without symptoms (e.g., pruritus, burning, tightness) | Macules/papules covering 10  - 30% BSA with or without symptoms (e.g., pruritus, burning, tightness); limiting instrumental ADL | Macules/papules covering  >30% BSA with or without associated symptoms; limiting self care ADL | - | - |
| Definition: A disorder characterized by the presence of macules (flat) and papules (elevated). Also known as morbillform rash, it is one of the most common cutaneous adverse events, frequently affecting the upper trunk, spreading centripetally and associated with pruritus. | | | | | |
| Scalp pain | Mild pain | Moderate pain; limiting instrumental ADL | Severe pain; limiting self care ADL | - | - |
| Definition: A disorder characterized by marked discomfort sensation in the skin covering the top and the back of the head. | | | | | |
| Skin atrophy | Covering <10% BSA; associated with telangiectasias or changes in skin color | Covering 10 - 30% BSA; associated with striae or adnexal structure loss | Covering >30% BSA; associated with ulceration | - | - |
| Definition: A disorder characterized by the degeneration and thinning of the epidermis and dermis. | | | | | |
| Skin hyperpigmentation | Hyperpigmentation covering  <10% BSA; no psychosocial impact | Hyperpigmentation covering  >10% BSA; associated psychosocial impact | - | - | - |
| Definition: A disorder characterized by darkening of the skin due to excessive melanin deposition. | | | | | |
| Skin hypopigmentation | Hypopigmentation or depigmentation covering  <10% BSA; no psychosocial  impact | Hypopigmentation or depigmentation covering  >10% BSA; associated  psychosocial impact | - | - | - |

| **Skin and subcutaneous tissue disorders** | | | | | |
| --- | --- | --- | --- | --- | --- |
|  | **Grade** | | | | |
| **Adverse Event** | **1** | **2** | **3** | **4** | **5** |
| Definition: A disorder characterized by loss of skin pigment. | | | | | |
| Skin induration | Mild induration, able to move skin parallel to plane (sliding) and perpendicular to skin (pinching up) | Moderate induration, able to slide skin, unable to pinch skin; limiting instrumental ADL | Severe induration, unable to slide or pinch skin; limiting joint movement or orifice (e.g., mouth, anus); limiting self care ADL | Generalized; associated with signs or symptoms of impaired breathing or feeding | Death |
| Definition: A disorder characterized by an area of hardness in the skin. | | | | | |
| Skin ulceration | Combined area of ulcers <1 cm; nonblanchable erythema of intact skin with associated warmth or edema | Combined area of ulcers 1 - 2 cm; partial thickness skin loss involving skin or subcutaneous fat | Combined area of ulcers >2 cm; full-thickness skin loss involving damage to or necrosis of subcutaneous tissue that may extend down to fascia | Any size ulcer with extensive destruction, tissue necrosis, or damage to muscle, bone, or supporting structures with or without full thickness skin loss | Death |
| Definition: A disorder characterized by circumscribed, inflammatory and necrotic erosive lesion on the skin. | | | | | |
| Stevens-Johnson syndrome | - | - | Skin sloughing covering <10% BSA with associated signs (e.g., erythema, purpura, epidermal detachment and mucous membrane detachment) | Skin sloughing covering 10 - 30% BSA with associated signs (e.g., erythema, purpura, epidermal detachment and mucous membrane detachment) | Death |
| Definition: A disorder characterized by less than 10% total body skin area separation of dermis. The syndrome is thought to be a hypersensitivity complex affecting the skin and the mucous membranes. | | | | | |

| **Skin and subcutaneous tissue disorders** | | | | | |
| --- | --- | --- | --- | --- | --- |
|  | **Grade** | | | | |
| **Adverse Event** | **1** | **2** | **3** | **4** | **5** |
| Telangiectasia | Telangiectasias covering  <10% BSA | Telangiectasias covering  >10% BSA; associated with psychosocial impact | - | - | - |
| Definition: A disorder characterized by local dilatation of small vessels resulting in red discoloration of the skin or mucous membranes. | | | | | |
| Toxic epidermal necrolysis | - | - | - | Skin sloughing covering  >=30% BSA with associated symptoms (e.g., erythema, purpura, or epidermal detachment) | Death |
| Definition: A disorder characterized by greater than 30% total body skin area separation of dermis. The syndrome is thought to be a hypersensitivity complex affecting the skin and the mucous membranes. | | | | | |
| Urticaria | Urticarial lesions covering  <10% BSA; topical intervention indicated | Urticarial lesions covering 10 - 30% BSA; oral intervention indicated | Urticarial lesions covering  >30% BSA; IV intervention indicated | - | - |
| Definition: A disorder characterized by an itchy skin eruption characterized by wheals with pale interiors and well-defined red margins. | | | | | |
| Skin and subcutaneous tissue disorders - Other, specify | Asymptomatic or mild symptoms; clinical or diagnostic observations only; intervention not indicated | Moderate; minimal, local or noninvasive intervention indicated; limiting age- appropriate instrumental ADL | Severe or medically significant but not immediately life- threatening; hospitalization or prolongation of existing hospitalization indicated; disabling; limiting self care ADL | Life-threatening consequences; urgent intervention indicated | Death |

| **Social circumstances** | | | | | |
| --- | --- | --- | --- | --- | --- |
|  | **Grade** | | | | |
| **Adverse Event** | **1** | **2** | **3** | **4** | **5** |
| Menopause | Menopause occurring at age 46 - 53 years of age | Menopause occurring at age 40 - 45 years of age | Menopause occurring before age 40 years of age | - | - |
| Definition: A disorder characterized by the permanent cessation of menses, usually defined by 12 consecutive months of amenorrhea in a woman over 45 years of age. | | | | | |
| Social circumstances - Other, specify | Asymptomatic or mild symptoms; clinical or diagnostic observations only; intervention not indicated | Moderate; minimal, local or noninvasive intervention indicated; limiting age- appropriate instrumental ADL | Severe or medically significant but not immediately life- threatening; hospitalization or prolongation of existing hospitalization indicated; disabling; limiting self care ADL | Life-threatening consequences; urgent intervention indicated | Death |

| **Immune system disorders** | | | | | |
| --- | --- | --- | --- | --- | --- |
|  | **Grade** | | | | |
| **Adverse Event** | **1** | **2** | **3** | **4** | **5** |
| Allergic reaction | Transient flushing or rash, drug fever <38 degrees C (<100.4 degrees F); intervention not indicated | Intervention or infusion interruption indicated; responds promptly to symptomatic treatment (e.g., antihistamines, NSAIDS, narcotics); prophylactic medications indicated for  <=24 hrs | Prolonged (e.g., not rapidly responsive to symptomatic medication and/or brief interruption of infusion); recurrence of symptoms following initial improvement; hospitalization indicated for clinical sequelae (e.g., renal impairment, pulmonary infiltrates) | Life-threatening consequences; urgent intervention indicated | Death |
| Definition: A disorder characterized by an adverse local or general response from exposure to an allergen. | | | | | |
| Anaphylaxis | - | - | Symptomatic bronchospasm, with or without urticaria; parenteral intervention indicated; allergy-related edema/angioedema; hypotension | Life-threatening consequences; urgent intervention indicated | Death |
| Definition: A disorder characterized by an acute inflammatory reaction resulting from the release of histamine and histamine-like substances from mast cells, causing a hypersensitivity immune response. Clinically, it presents with breathing difficulty, dizziness, hypotension, cyanosis and loss of consciousness and may lead to death. | | | | | |
| Autoimmune disorder | Asymptomatic; serologic or other evidence of autoimmune reaction, with normal organ function; intervention not indicated | Evidence of autoimmune reaction involving a non- essential organ or function (e.g., hypothyroidism) | Autoimmune reactions involving major organ (e.g., colitis, anemia, myocarditis, kidney) | Life-threatening consequences; urgent intervention indicated | Death |

| **Immune system disorders** | | | | | |
| --- | --- | --- | --- | --- | --- |
|  | **Grade** | | | | |
| **Adverse Event** | **1** | **2** | **3** | **4** | **5** |
| Definition: A disorder resulting from loss of function or tissue destruction of an organ or multiple organs, arising from humoral or cellular immune responses of the individual to his own tissue constituents. | | | | | |
| Cytokine release syndrome | Mild reaction; infusion interruption not indicated; intervention not indicated | Therapy or infusion interruption indicated but responds promptly to symptomatic treatment (e.g., antihistamines, NSAIDS, narcotics, IV fluids); prophylactic medications indicated for <=24 hrs | Prolonged (e.g., not rapidly responsive to symptomatic medication and/or brief interruption of infusion); recurrence of symptoms following initial improvement; hospitalization indicated for clinical sequelae (e.g., renal impairment, pulmonary infiltrates) | Life-threatening consequences; pressor or ventilatory support indicated | Death |
| Definition: A disorder characterized by nausea, headache, tachycardia, hypotension, rash, and shortness of breath; it is caused by the release of cytokines from the cells. | | | | | |
| Serum sickness | Asymptomatic; clinical or diagnostic observations only; intervention not indicated | Moderate arthralgia; fever, rash, urticaria, antihistamines indicated | Severe arthralgia or arthritis; extensive rash; steroids or IV fluids indicated | Life-threatening consequences; pressor or ventilatory support indicated | Death |
| Definition: A disorder characterized by a delayed-type hypersensitivity reaction to foreign proteins derived from an animal serum. It occurs approximately six to twenty-one days following the administration of the foreign antigen. Symptoms include fever, arthralgias, myalgias, skin eruptions, lymphadenopathy, chest marked discomfort and dyspnea. | | | | | |

| **Immune system disorders** | | | | | |
| --- | --- | --- | --- | --- | --- |
|  | **Grade** | | | | |
| **Adverse Event** | **1** | **2** | **3** | **4** | **5** |
| Immune system disorders - Other, specify | Asymptomatic or mild symptoms; clinical or diagnostic observations only; intervention not indicated | Moderate; minimal, local or noninvasive intervention indicated; limiting age- appropriate instrumental ADL | Severe or medically significant but not immediately life- threatening; hospitalization or prolongation of existing hospitalization indicated; disabling; limiting self care ADL | Life-threatening consequences; urgent intervention indicated | Death |

| **Infections and infestations** | | | | | |
| --- | --- | --- | --- | --- | --- |
|  | **Grade** | | | | |
| **Adverse Event** | **1** | **2** | **3** | **4** | **5** |
| Abdominal infection | - | - | IV antibiotic, antifungal, or antiviral intervention indicated; radiologic or operative intervention indicated | Life-threatening consequences; urgent intervention indicated | Death |
| Definition: A disorder characterized by an infectious process involving the abdominal cavity. | | | | | |
| Anorectal infection | Localized; local intervention indicated | Oral intervention indicated (e.g., antibiotic, antifungal, antiviral) | IV antibiotic, antifungal, or antiviral intervention indicated; radiologic, endoscopic, or operative intervention indicated | Life-threatening consequences; urgent intervention indicated | Death |
| Definition: A disorder characterized by an infectious process involving the anal area and the rectum. | | | | | |
| Appendicitis | - | - | IV antibiotic, antifungal, or antiviral intervention indicated; radiologic or operative intervention indicated | Life-threatening consequences; urgent intervention indicated | Death |
| Definition: A disorder characterized by acute inflammation to the vermiform appendix caused by a pathogenic agent. | | | | | |
| Appendicitis perforated | - | Symptomatic; medical intervention indicated | Severe symptoms; elective operative intervention indicated | Life-threatening consequences; urgent intervention indicated | Death |
| Definition: A disorder characterized by acute inflammation to the vermiform appendix caused by a pathogenic agent with gangrenous changes resulting in the rupture of the appendiceal wall. The appendiceal wall rupture causes the release of inflammatory and bacterial contents from the appendiceal lumen into the abdominal cavity. | | | | | |

| **Infections and infestations** | | | | | |
| --- | --- | --- | --- | --- | --- |
|  | **Grade** | | | | |
| **Adverse Event** | **1** | **2** | **3** | **4** | **5** |
| Arteritis infective | - | - | IV antibiotic, antifungal, or antiviral intervention indicated; radiologic or operative intervention indicated | Life-threatening consequences; urgent intervention indicated | Death |
| Definition: A disorder characterized by an infectious process involving an artery. | | | | | |
| Biliary tract infection | - | - | IV antibiotic, antifungal, or antiviral intervention indicated; radiologic or operative intervention indicated | Life-threatening consequences; urgent intervention indicated | Death |
| Definition: A disorder characterized by an infectious process involving the biliary tract. | | | | | |
| Bladder infection | - | Oral intervention indicated (e.g., antibiotic, antifungal, antiviral) | IV antibiotic, antifungal, or antiviral intervention indicated; radiologic, endoscopic, or operative intervention indicated | Life-threatening consequences; urgent intervention indicated | Death |
| Definition: A disorder characterized by an infectious process involving the bladder. | | | | | |
| Bone infection | - | - | IV antibiotic, antifungal, or antiviral intervention indicated; radiologic or operative intervention indicated | Life-threatening consequences; urgent intervention indicated | Death |
| Definition: A disorder characterized by an infectious process involving the bones. | | | | | |

| **Infections and infestations** | | | | | |
| --- | --- | --- | --- | --- | --- |
|  | **Grade** | | | | |
| **Adverse Event** | **1** | **2** | **3** | **4** | **5** |
| Breast infection | - | Local infection with moderate symptoms; oral intervention indicated (e.g., antibiotic, antifungal, antiviral) | Severe infection; axillary adenitis; IV antibacterial, antifungal, or antiviral intervention indicated | Life-threatening consequences; urgent intervention indicated | Death |
| Definition: A disorder characterized by an infectious process involving the breast. | | | | | |
| Bronchial infection | - | Moderate symptoms; oral intervention indicated (e.g., antibiotic, antifungal, antiviral) | IV antibiotic, antifungal, or antiviral intervention indicated; radiologic, endoscopic, or operative intervention indicated | Life-threatening consequences; urgent intervention indicated | Death |
| Definition: A disorder characterized by an infectious process involving the bronchi. | | | | | |
| Catheter related infection | - | Localized; local intervention indicated; oral intervention indicated (e.g., antibiotic, antifungal, antiviral) | IV antibiotic, antifungal, or antiviral intervention indicated; radiologic or operative intervention indicated | Life-threatening consequences; urgent intervention indicated | Death |
| Definition: A disorder characterized by an infectious process that arises secondary to catheter use. | | | | | |
| Cecal infection | - | - | IV antibiotic, antifungal, or antiviral intervention indicated; radiologic, endoscopic, or operative intervention indicated | Life-threatening consequences; urgent intervention indicated | Death |
| Definition: A disorder characterized by an infectious process involving the cecum. | | | | | |

| **Infections and infestations** | | | | | |
| --- | --- | --- | --- | --- | --- |
|  | **Grade** | | | | |
| **Adverse Event** | **1** | **2** | **3** | **4** | **5** |
| Cervicitis infection | - | Localized; local intervention indicated (e.g., topical antibiotic, antifungal, or antiviral) | IV antibiotic, antifungal, or antiviral intervention indicated; radiologic or operative intervention indicated | Life-threatening consequences; urgent intervention indicated | Death |
| Definition: A disorder characterized by an infectious process involving the uterine cervix. | | | | | |
| Conjunctivitis infective | - | Localized; local intervention indicated (e.g., topical antibiotic, antifungal, or antiviral) | IV antibiotic, antifungal, or antiviral intervention indicated; radiologic or operative intervention indicated | Life-threatening consequences; urgent intervention indicated | Death |
| Definition: A disorder characterized by an infectious process involving the conjunctiva. Clinical manifestations include pink or red color in the eyes. | | | | | |
| Corneal infection | - | Localized; local intervention indicated (e.g., topical antibiotic, antifungal, or antiviral) | IV antibiotic, antifungal, or antiviral intervention indicated; radiologic or operative intervention indicated | Life-threatening consequences; urgent intervention indicated | Death |
| Definition: A disorder characterized by an infectious process involving the cornea. | | | | | |
| Cranial nerve infection | - | - | IV antibiotic, antifungal, or antiviral intervention indicated; radiologic or operative intervention indicated | Life-threatening consequences; urgent intervention indicated | Death |
| Definition: A disorder characterized by an infectious process involving a cranial nerve. | | | | | |

| **Infections and infestations** | | | | | |
| --- | --- | --- | --- | --- | --- |
|  | **Grade** | | | | |
| **Adverse Event** | **1** | **2** | **3** | **4** | **5** |
| Device related infection | - | - | IV antibiotic, antifungal, or antiviral intervention indicated; radiologic or operative intervention indicated | Life-threatening consequences; urgent intervention indicated | Death |
| Definition: A disorder characterized by an infectious process involving the use of a medical device. | | | | | |
| Duodenal infection | - | Moderate symptoms; medical intervention indicated (e.g., oral antibiotics) | IV antibiotic, antifungal, or antiviral intervention indicated; radiologic or operative intervention indicated | Life-threatening consequences; urgent intervention indicated | Death |
| Definition: A disorder characterized by an infectious process involving the duodenum. | | | | | |
| Encephalitis infection | - | - | IV antibiotic, antifungal, or antiviral intervention indicated; severe changes in mental status; self-limited seizure activity; focal neurologic abnormalities | Life-threatening consequences; urgent intervention indicated | Death |
| Definition: A disorder characterized by an infectious process involving the brain tissue. | | | | | |
| Encephalomyelitis infection | - | - | IV antibiotic, antifungal, or antiviral intervention indicated; radiologic or operative intervention indicated | Life-threatening consequences; urgent intervention indicated | Death |
| Definition: A disorder characterized by an infectious process involving the brain and spinal cord tissues. | | | | | |

| **Infections and infestations** | | | | | |
| --- | --- | --- | --- | --- | --- |
|  | **Grade** | | | | |
| **Adverse Event** | **1** | **2** | **3** | **4** | **5** |
| Endocarditis infective | - | - | IV antibiotic, antifungal, or antiviral intervention indicated; radiologic or operative intervention indicated | Life-threatening consequences; urgent intervention indicated | Death |
| Definition: A disorder characterized by an infectious process involving the endocardial layer of the heart. | | | | | |
| Endophthalmitis | - | Local intervention indicated | Systemic intervention or hospitalization indicated | Blindness (20/200 or worse) | - |
| Definition: A disorder characterized by an infectious process involving the internal structures of the eye. | | | | | |
| Enterocolitis infectious | - | Passage of >3 unformed stools per 24 hrs or duration of illness >48 hrs; moderate abdominal pain | IV antibiotic, antifungal, or antiviral intervention indicated; radiologic, endoscopic, or operative intervention indicated; profuse watery diarrhea with signs of hypovolemia; bloody diarrhea; fever; severe abdominal pain; hospitalization indicated | Life-threatening consequences; urgent intervention indicated | Death |
| Definition: A disorder characterized by an infectious process involving the small and large intestines. | | | | | |
| Esophageal infection | - | Local intervention indicated (e.g., oral antibiotic, antifungal, antiviral) | IV antibiotic, antifungal, or antiviral intervention indicated; radiologic or operative intervention indicated | Life-threatening consequences; urgent intervention indicated | Death |

| **Infections and infestations** | | | | | |
| --- | --- | --- | --- | --- | --- |
|  | **Grade** | | | | |
| **Adverse Event** | **1** | **2** | **3** | **4** | **5** |
| Definition: A disorder characterized by an infectious process involving the esophagus. | | | | | |
| Eye infection | - | Localized; local intervention indicated (e.g., topical antibiotic, antifungal, or antiviral) | IV antibiotic, antifungal, or antiviral intervention indicated; radiologic or operative intervention indicated | Life-threatening consequences; urgent intervention indicated; enucleation | Death |
| Definition: A disorder characterized by an infectious process involving the eye. | | | | | |
| Gallbladder infection | - | - | IV antibiotic, antifungal, or antiviral intervention indicated; radiologic, endoscopic, or operative intervention indicated | Life-threatening consequences; urgent intervention indicated | Death |
| Definition: A disorder characterized by an infectious process involving the gallbladder. | | | | | |
| Gum infection | Local therapy indicated (swish and swallow) | Moderate symptoms; oral intervention indicated (e.g., antibiotic, antifungal, antiviral) | IV antibiotic, antifungal, or antiviral intervention indicated; radiologic or operative intervention indicated | Life-threatening consequences; urgent intervention indicated | Death |
| Definition: A disorder characterized by an infectious process involving the gums. | | | | | |
| Hepatic infection | - | - | IV antibiotic, antifungal, or antiviral intervention indicated; radiologic or operative intervention indicated | Life-threatening consequences; urgent intervention indicated | Death |
| Definition: A disorder characterized by an infectious process involving the liver. | | | | | |

| **Infections and infestations** | | | | | |
| --- | --- | --- | --- | --- | --- |
|  | **Grade** | | | | |
| **Adverse Event** | **1** | **2** | **3** | **4** | **5** |
| Hepatitis viral | Asymptomatic, treatment not indicated | - | Symptomatic liver dysfunction; fibrosis by biopsy; compensated cirrhosis; reactivation of chronic hepatitis | Decompensated liver function (e.g., ascites, coagulopathy, encephalopathy, coma) | Death |
| Definition: A disorder characterized by a viral pathologic process involving the liver parenchyma. | | | | | |
| Infective myositis | - | Localized; local intervention indicated (e.g., topical antibiotic, antifungal, or antiviral) | IV antibiotic, antifungal, or antiviral intervention indicated; radiologic or operative intervention indicated | Life-threatening consequences; urgent intervention indicated | Death |
| Definition: A disorder characterized by an infectious process involving the skeletal muscles. | | | | | |
| Joint infection | - | Localized; local intervention indicated; oral intervention indicated (e.g., antibiotic, antifungal, antiviral); needle aspiration indicated (single or multiple) | Arthroscopic intervention indicated (e.g., drainage) or arthrotomy (e.g., open surgical drainage) | Life-threatening consequences; urgent intervention indicated | Death |
| Definition: A disorder characterized by an infectious process involving a joint. | | | | | |
| Kidney infection | - | - | IV antibiotic, antifungal, or antiviral intervention indicated; radiologic, endoscopic, or operative intervention indicated | Life-threatening consequences; urgent intervention indicated | Death |

| **Infections and infestations** | | | | | |
| --- | --- | --- | --- | --- | --- |
|  | **Grade** | | | | |
| **Adverse Event** | **1** | **2** | **3** | **4** | **5** |
| Definition: A disorder characterized by an infectious process involving the kidney. | | | | | |
| Laryngitis | - | Moderate symptoms; oral intervention indicated (e.g., antibiotic, antifungal, antiviral) | IV antibiotic, antifungal, or antiviral intervention indicated; radiologic or operative intervention indicated | Life-threatening consequences; urgent intervention indicated | Death |
| Definition: A disorder characterized by an inflammatory process involving the larynx. | | | | | |
| Lip infection | Localized, local intervention indicated | Oral intervention indicated (e.g., antibiotic, antifungal, antiviral) | IV antibiotic, antifungal, or antiviral intervention indicated; radiologic or operative intervention indicated | - | - |
| Definition: A disorder characterized by an infectious process involving the lips. | | | | | |
| Lung infection | - | Moderate symptoms; oral intervention indicated (e.g., antibiotic, antifungal, antiviral) | IV antibiotic, antifungal, or antiviral intervention indicated; radiologic, endoscopic, or operative intervention indicated | Life-threatening consequences; urgent intervention indicated | Death |
| Definition: A disorder characterized by an infectious process involving the lungs. | | | | | |
| Lymph gland infection | - | Localized; local intervention indicated (e.g., topical antibiotic, antifungal, or antiviral) | IV antibiotic, antifungal, or antiviral intervention indicated; radiologic or operative intervention indicated | Life-threatening consequences; urgent intervention indicated | Death |
| Definition: A disorder characterized by an infectious process involving the lymph nodes. | | | | | |

| **Infections and infestations** | | | | | |
| --- | --- | --- | --- | --- | --- |
|  | **Grade** | | | | |
| **Adverse Event** | **1** | **2** | **3** | **4** | **5** |
| Mediastinal infection | - | - | IV antibiotic, antifungal, or antiviral intervention indicated; radiologic or operative intervention indicated | Life-threatening consequences; urgent intervention indicated | Death |
| Definition: A disorder characterized by an infectious process involving the mediastinum. | | | | | |
| Meningitis | - | - | IV antibiotic, antifungal, or antiviral intervention indicated; radiologic or operative intervention indicated; focal neurologic deficit | Life-threatening consequences; urgent intervention indicated | Death |
| Definition: A disorder characterized by acute inflammation of the meninges of the brain and/or spinal cord. | | | | | |
| Mucosal infection | Localized, local intervention indicated | Oral intervention indicated (e.g., antibiotic, antifungal, antiviral) | IV antibiotic, antifungal, or antiviral intervention indicated; radiologic or operative intervention indicated | Life-threatening consequences; urgent intervention indicated | Death |
| Definition: A disorder characterized by an infectious process involving a mucosal surface. | | | | | |
| Nail infection | Localized, local intervention indicated | Oral intervention indicated (e.g., antibiotic, antifungal, antiviral) | IV antibiotic, antifungal, or antiviral intervention indicated; radiologic or operative intervention indicated | - | - |
| Definition: A disorder characterized by an infectious process involving the nail. | | | | | |

| **Infections and infestations** | | | | | |
| --- | --- | --- | --- | --- | --- |
|  | **Grade** | | | | |
| **Adverse Event** | **1** | **2** | **3** | **4** | **5** |
| Otitis externa | - | Localized; local intervention indicated (e.g., topical antibiotic, antifungal, or antiviral) | IV antibiotic, antifungal, or antiviral intervention indicated; radiologic or operative intervention indicated | Life-threatening consequences; urgent intervention indicated | Death |
| Definition: A disorder characterized by an infectious process involving the outer ear and ear canal. Contributory factors include excessive water exposure (swimmer's ear infection) and cuts in the ear canal. Symptoms include fullness, itching, swelling and marked discomfort in the ear and ear drainage. | | | | | |
| Otitis media | - | Localized; local intervention indicated (e.g., topical antibiotic, antifungal, or antiviral) | IV antibiotic, antifungal, or antiviral intervention indicated; radiologic or operative intervention indicated | Life-threatening consequences; urgent intervention indicated | Death |
| Definition: A disorder characterized by an infectious process involving the middle ear. | | | | | |
| Ovarian infection | - | Localized; local intervention indicated (e.g., topical antibiotic, antifungal, or antiviral) | IV antibiotic, antifungal, or antiviral intervention indicated; radiologic or operative intervention indicated | Life-threatening consequences; urgent intervention indicated | Death |
| Definition: A disorder characterized by an infectious process involving the ovary. | | | | | |
| Pancreas infection | - | - | IV antibiotic, antifungal, or antiviral intervention indicated; radiologic or operative intervention indicated | Life-threatening consequences; urgent intervention indicated | Death |
| Definition: A disorder characterized by an infectious process involving the pancreas. | | | | | |

| **Infections and infestations** | | | | | |
| --- | --- | --- | --- | --- | --- |
|  | **Grade** | | | | |
| **Adverse Event** | **1** | **2** | **3** | **4** | **5** |
| Papulopustular rash | Papules and/or pustules covering <10% BSA, which may or may not be associated with symptoms of pruritus or tenderness | Papules and/or pustules covering 10-30% BSA, which may or may not be associated with symptoms of pruritus or tenderness; associated with psychosocial impact; limiting instrumental ADL | Papules and/or pustules covering >30% BSA, which may or may not be associated with symptoms of pruritus or tenderness; limiting self-care ADL; associated with local superinfection with oral antibiotics indicated | Papules and/or pustules covering any % BSA, which may or may not be associated with symptoms of pruritus or tenderness and are associated with extensive superinfection with IV antibiotics indicated; life- threatening consequences | Death |
| Definition: A disorder characterized by an eruption consisting of papules (a small, raised pimple) and pustules (a small pus filled blister), typically appearing in face, scalp, and upper chest and back Unlike acne, this rash does not present with whiteheads or blackheads, and can be symptomatic, with itchy or tender lesions. | | | | | |
| Paronychia | Nail fold edema or erythema; disruption of the cuticle | Localized intervention indicated; oral intervention indicated (e.g., antibiotic, antifungal, antiviral); nail fold edema or erythema with pain; associated with discharge or nail plate separation; limiting instrumental ADL | Surgical intervention or IV antibiotics indicated; limiting self care ADL | - | - |
| Definition: A disorder characterized by an infectious process involving the soft tissues around the nail. | | | | | |
| Pelvic infection | - | Moderate symptoms; oral intervention indicated (e.g., antibiotic, antifungal, antiviral) | IV antibiotic, antifungal, or antiviral intervention indicated; radiologic or operative intervention indicated | Life-threatening consequences; urgent intervention indicated | Death |

| **Infections and infestations** | | | | | |
| --- | --- | --- | --- | --- | --- |
|  | **Grade** | | | | |
| **Adverse Event** | **1** | **2** | **3** | **4** | **5** |
| Pharyngitis | - | Localized; local intervention indicated (e.g., topical antibiotic, antifungal, or antiviral) | IV antibiotic, antifungal, or antiviral intervention indicated; radiologic or operative intervention indicated | Life-threatening consequences; urgent intervention indicated | Death |
| Definition: A disorder characterized by inflammation of the throat. | | | | | |
| Phlebitis infective | - | Localized; local intervention indicated (e.g., topical antibiotic, antifungal, or antiviral) | IV antibiotic, antifungal, or antiviral intervention indicated; radiologic or operative intervention indicated | Life-threatening consequences; urgent intervention indicated | Death |
| Definition: A disorder characterized by an infectious process involving the vein. Clinical manifestations include erythema, marked discomfort, swelling, and induration along the course of the infected vein. | | | | | |
| Pleural infection | - | Localized; local intervention indicated (e.g., topical antibiotic, antifungal, or antiviral) | IV antibiotic, antifungal, or antiviral intervention indicated; radiologic, endoscopic, or operative intervention indicated | Life-threatening consequences; urgent intervention indicated | Death |
| Definition: A disorder characterized by an infectious process involving the pleura. | | | | | |
| Prostate infection | - | Moderate symptoms; oral intervention indicated (e.g., antibiotic, antifungal, antiviral) | IV antibiotic, antifungal, or antiviral intervention indicated; radiologic, endoscopic, or operative intervention indicated | Life-threatening consequences; urgent intervention indicated | Death |
| Definition: A disorder characterized by an infectious process involving the prostate gland. | | | | | |

| **Infections and infestations** | | | | | |
| --- | --- | --- | --- | --- | --- |
|  | **Grade** | | | | |
| **Adverse Event** | **1** | **2** | **3** | **4** | **5** |
| Rash pustular | - | Localized; local intervention indicated (e.g., topical antibiotic, antifungal, or antiviral) | IV antibiotic, antifungal, or antiviral intervention indicated; radiologic or operative intervention indicated | - | - |
| Definition: A disorder characterized by a circumscribed and elevated skin lesion filled with pus. | | | | | |
| Rhinitis infective | - | Localized; local intervention indicated (e.g., topical antibiotic, antifungal, or antiviral) | - | - | - |
| Definition: A disorder characterized by an infectious process involving the nasal mucosal. | | | | | |
| Salivary gland infection | - | Moderate symptoms; oral intervention indicated (e.g., antibiotic, antifungal, antiviral) | IV antibiotic, antifungal, or antiviral intervention indicated; radiologic or operative intervention indicated | Life-threatening consequences; urgent intervention indicated | Death |
| Definition: A disorder characterized by an infectious process involving the salivary gland. | | | | | |
| Scrotal infection | - | Localized; local intervention indicated (e.g., topical antibiotic, antifungal, or antiviral) | IV antibiotic, antifungal, or antiviral intervention indicated; radiologic or operative intervention indicated | Life-threatening consequences; urgent intervention indicated | Death |
| Definition: A disorder characterized by an infectious process involving the scrotum. | | | | | |
| Sepsis | - | - | - | Life-threatening consequences; urgent intervention indicated | Death |

| **Infections and infestations** | | | | | |
| --- | --- | --- | --- | --- | --- |
|  | **Grade** | | | | |
| **Adverse Event** | **1** | **2** | **3** | **4** | **5** |
| Definition: A disorder characterized by the presence of pathogenic microorganisms in the blood stream that cause a rapidly progressing systemic reaction that may lead to shock. | | | | | |
| Sinusitis | - | Localized; local intervention indicated (e.g., topical antibiotic, antifungal, or antiviral) | IV antibiotic, antifungal, or antiviral intervention indicated; radiologic, endoscopic, or operative intervention indicated | Life-threatening consequences; urgent intervention indicated | Death |
| Definition: A disorder characterized by an infectious process involving the mucous membranes of the paranasal sinuses. | | | | | |
| Skin infection | Localized, local intervention indicated | Oral intervention indicated (e.g., antibiotic, antifungal, antiviral) | IV antibiotic, antifungal, or antiviral intervention indicated; radiologic or operative intervention indicated | Life-threatening consequences; urgent intervention indicated | Death |
| Definition: A disorder characterized by an infectious process involving the skin. | | | | | |
| Small intestine infection | - | Moderate symptoms; oral intervention indicated (e.g., antibiotic, antifungal, antiviral) | IV antibiotic, antifungal, or antiviral intervention indicated; radiologic or operative intervention indicated | Life-threatening consequences; urgent intervention indicated | Death |
| Definition: A disorder characterized by an infectious process involving the small intestine. | | | | | |
| Soft tissue infection | - | Localized; local intervention indicated (e.g., topical antibiotic, antifungal, or antiviral) | IV antibiotic, antifungal, or antiviral intervention indicated; radiologic or operative intervention indicated | Life-threatening consequences; urgent intervention indicated | Death |
| Definition: A disorder characterized by an infectious process involving soft tissues. | | | | | |

| **Infections and infestations** | | | | | |
| --- | --- | --- | --- | --- | --- |
|  | **Grade** | | | | |
| **Adverse Event** | **1** | **2** | **3** | **4** | **5** |
| Splenic infection | - | - | IV antibiotic, antifungal, or antiviral intervention indicated; radiologic or operative intervention indicated | Life-threatening consequences; urgent intervention indicated | Death |
| Definition: A disorder characterized by an infectious process involving the spleen. | | | | | |
| Stoma site infection | Localized, local intervention indicated | Oral intervention indicated (e.g., antibiotic, antifungal, antiviral) | IV antibiotic, antifungal, or antiviral intervention indicated; radiologic, endoscopic, or operative intervention indicated | Life-threatening consequences; urgent intervention indicated | Death |
| Definition: A disorder characterized by an infectious process involving a stoma (surgically created opening on the surface of the body). | | | | | |
| Tooth infection | - | Localized; local intervention indicated (e.g., topical antibiotic, antifungal, or antiviral) | IV antibiotic, antifungal, or antiviral intervention indicated; radiologic or operative intervention indicated | Life-threatening consequences; urgent intervention indicated | Death |
| Definition: A disorder characterized by an infectious process involving a tooth. | | | | | |
| Tracheitis | - | Moderate symptoms; oral intervention indicated (e.g., antibiotic, antifungal, antiviral) | IV antibiotic, antifungal, or antiviral intervention indicated; radiologic, endoscopic, or operative intervention indicated | Life-threatening consequences; urgent intervention indicated | Death |
| Definition: A disorder characterized by an infectious process involving the trachea. | | | | | |

| **Infections and infestations** | | | | | |
| --- | --- | --- | --- | --- | --- |
|  | **Grade** | | | | |
| **Adverse Event** | **1** | **2** | **3** | **4** | **5** |
| Upper respiratory infection | - | Moderate symptoms; oral intervention indicated (e.g., antibiotic, antifungal, antiviral) | IV antibiotic, antifungal, or antiviral intervention indicated; radiologic, endoscopic, or operative intervention indicated | Life-threatening consequences; urgent intervention indicated | Death |
| Definition: A disorder characterized by an infectious process involving the upper respiratory tract (nose, paranasal sinuses, pharynx, larynx, or trachea). | | | | | |
| Urethral infection | - | Localized; local intervention indicated (e.g., topical antibiotic, antifungal, or antiviral) | IV antibiotic, antifungal, or antiviral intervention indicated; radiologic, endoscopic, or operative intervention indicated | Life-threatening consequences; urgent intervention indicated | Death |
| Definition: A disorder characterized by an infectious process involving the urethra. | | | | | |
| Urinary tract infection | - | Localized; local intervention indicated (e.g., topical antibiotic, antifungal, or antiviral) | IV antibiotic, antifungal, or antiviral intervention indicated; radiologic or operative intervention indicated | Life-threatening consequences; urgent intervention indicated | Death |
| Definition: A disorder characterized by an infectious process involving the urinary tract, most commonly the bladder and the urethra. | | | | | |
| Uterine infection | - | Moderate symptoms; oral intervention indicated (e.g., antibiotic, antifungal, antiviral) | IV antibiotic, antifungal, or antiviral intervention indicated; radiologic or operative intervention indicated | Life-threatening consequences; urgent intervention indicated | Death |
| Definition: A disorder characterized by an infectious process involving the endometrium. It may extend to the myometrium and parametrial tissues. | | | | | |

| **Infections and infestations** | | | | | |
| --- | --- | --- | --- | --- | --- |
|  | **Grade** | | | | |
| **Adverse Event** | **1** | **2** | **3** | **4** | **5** |
| Vaginal infection | - | Localized; local intervention indicated (e.g., topical antibiotic, antifungal, or antiviral) | IV antibiotic, antifungal, or antiviral intervention indicated; radiologic or operative intervention indicated | Life-threatening consequences; urgent intervention indicated | Death |
| Definition: A disorder characterized by an infectious process involving the vagina. | | | | | |
| Vulval infection | Localized, local intervention indicated | Oral intervention indicated (e.g., antibiotic, antifungal, antiviral) | IV antibiotic, antifungal, or antiviral intervention indicated; radiologic or operative intervention indicated | Life-threatening consequences; urgent intervention indicated | Death |
| Definition: A disorder characterized by an infectious process involving the vulva. | | | | | |
| Wound infection | - | Localized; local intervention indicated (e.g., topical antibiotic, antifungal, or antiviral) | IV antibiotic, antifungal, or antiviral intervention indicated; radiologic or operative intervention indicated | Life-threatening consequences; urgent intervention indicated | Death |
| Definition: A disorder characterized by an infectious process involving the wound. | | | | | |
| Infections and infestations - Other, specify | Asymptomatic or mild symptoms; clinical or diagnostic observations only; intervention not indicated | Moderate; minimal, local or noninvasive intervention indicated; limiting age- appropriate instrumental ADL | Severe or medically significant but not immediately life- threatening; hospitalization or prolongation of existing hospitalization indicated; disabling; limiting self care ADL | Life-threatening consequences; urgent intervention indicated | Death |

| **Nervous system disorders** | | | | | |
| --- | --- | --- | --- | --- | --- |
|  | **Grade** | | | | |
| **Adverse Event** | **1** | **2** | **3** | **4** | **5** |
| Abducens nerve disorder | Asymptomatic; clinical or diagnostic observations only; intervention not indicated | Moderate symptoms; limiting instrumental ADL | Severe symptoms; limiting self care ADL | - | - |
| Definition: A disorder characterized by involvement of the abducens nerve (sixth cranial nerve). | | | | | |
| Accessory nerve disorder | Asymptomatic; clinical or diagnostic observations only; intervention not indicated | Moderate symptoms; limiting instrumental ADL | Severe symptoms; limiting self care ADL | - | - |
| Definition: A disorder characterized by involvement of the accessory nerve (eleventh cranial nerve). | | | | | |
| Acoustic nerve disorder NOS | Asymptomatic; clinical or diagnostic observations only; intervention not indicated | Moderate symptoms; limiting instrumental ADL | Severe symptoms; limiting self care ADL | - | - |
| Definition: A disorder characterized by involvement of the acoustic nerve (eighth cranial nerve). | | | | | |
| Akathisia | Mild restlessness or increased motor activity | Moderate restlessness or increased motor activity; limiting instrumental ADL | Severe restlessness or increased motor activity; limiting self care ADL | - | - |
| Definition: A disorder characterized by an uncomfortable feeling of inner restlessness and inability to stay still; this is a side effect of some psychotropic drugs. | | | | | |
| Amnesia | Mild; transient memory loss | Moderate; short term memory loss; limiting instrumental ADL | Severe; long term memory loss; limiting self care ADL | - | - |
| Definition: A disorder characterized by systematic and extensive loss of memory. | | | | | |
| Aphonia | - | - | Voicelessness; unable to speak | - | - |
| Definition: A disorder characterized by the inability to speak. It may result from injuries to the vocal cords or may be functional (psychogenic). | | | | | |

| **Nervous system disorders** | | | | | |
| --- | --- | --- | --- | --- | --- |
|  | **Grade** | | | | |
| **Adverse Event** | **1** | **2** | **3** | **4** | **5** |
| Arachnoiditis | Mild symptoms | Moderate symptoms; limiting instrumental ADL | Severe symptoms; limiting self care ADL | Life-threatening consequences; urgent intervention indicated | Death |
| Definition: A disorder characterized by inflammation of the arachnoid membrane and adjacent subarachnoid space. | | | | | |
| Ataxia | Asymptomatic; clinical or diagnostic observations only; intervention not indicated | Moderate symptoms; limiting instrumental ADL | Severe symptoms; limiting self care ADL; mechanical assistance indicated | - | - |
| Definition: A disorder characterized by lack of coordination of muscle movements resulting in the impairment or inability to perform voluntary activities. | | | | | |
| Brachial plexopathy | Asymptomatic; clinical or diagnostic observations only; intervention not indicated | Moderate symptoms; limiting instrumental ADL | Severe symptoms; limiting self care ADL | - | - |
| Definition: A disorder characterized by regional paresthesia of the brachial plexus, marked discomfort and muscle weakness, and limited movement in the arm or hand. | | | | | |
| Central nervous system necrosis | Asymptomatic; clinical or diagnostic observations only; intervention not indicated | Moderate symptoms; corticosteroids indicated | Severe symptoms; medical intervention indicated | Life-threatening consequences; urgent intervention indicated | Death |
| Definition: A disorder characterized by a necrotic process occurring in the brain and/or spinal cord. | | | | | |
| Cerebrospinal fluid leakage | Post-craniotomy: asymptomatic; Post-lumbar puncture: transient headache; postural care indicated | Post-craniotomy: moderate symptoms; medical intervention indicated; Post- lumbar puncture: persistent moderate symptoms; blood patch indicated | Severe symptoms; medical intervention indicated | Life-threatening consequences; urgent intervention indicated | Death |
| Definition: A disorder characterized by loss of cerebrospinal fluid into the surrounding tissues. | | | | | |

| **Nervous system disorders** | | | | | |
| --- | --- | --- | --- | --- | --- |
|  | **Grade** | | | | |
| **Adverse Event** | **1** | **2** | **3** | **4** | **5** |
| Cognitive disturbance | Mild cognitive disability; not interfering with work/school/life performance; specialized educational services/devices not indicated | Moderate cognitive disability; interfering with work/school/life performance but capable of independent living; specialized resources on part time basis indicated | Severe cognitive disability; significant impairment of work/school/life performance | - | - |
| Definition: A disorder characterized by a conspicuous change in cognitive function. | | | | | |
| Concentration impairment | Mild inattention or decreased level of concentration | Moderate impairment in attention or decreased level of concentration; limiting instrumental ADL | Severe impairment in attention or decreased level of concentration; limiting self care ADL | - | - |
| Definition: A disorder characterized by a deterioration in the ability to concentrate. | | | | | |
| Depressed level of consciousness | Decreased level of alertness | Sedation; slow response to stimuli; limiting instrumental ADL | Difficult to arouse | Life-threatening consequences | Death |
| Definition: A disorder characterized by a decrease in ability to perceive and respond. | | | | | |
| Dizziness | Mild unsteadiness or sensation of movement | Moderate unsteadiness or sensation of movement; limiting instrumental ADL | Severe unsteadiness or sensation of movement; limiting self care ADL | - | - |
| Definition: A disorder characterized by a disturbing sensation of lightheadedness, unsteadiness, giddiness, spinning or rocking. | | | | | |
| Dysarthria | Mild slurred speech | Moderate impairment of articulation or slurred speech | Severe impairment of articulation or slurred speech | - | - |
| Definition: A disorder characterized by slow and slurred speech resulting from an inability to coordinate the muscles used in speech. | | | | | |

| **Nervous system disorders** | | | | | |
| --- | --- | --- | --- | --- | --- |
|  | **Grade** | | | | |
| **Adverse Event** | **1** | **2** | **3** | **4** | **5** |
| Dysesthesia | Mild sensory alteration | Moderate sensory alteration; limiting instrumental ADL | Severe sensory alteration; limiting self care ADL | - | - |
| Definition: A disorder characterized by distortion of sensory perception, resulting in an abnormal and unpleasant sensation. | | | | | |
| Dysgeusia | Altered taste but no change in diet | Altered taste with change in diet (e.g., oral supplements); noxious or unpleasant taste; loss of taste | - | - | - |
| Definition: A disorder characterized by abnormal sensual experience with the taste of foodstuffs; it can be related to a decrease in the sense of smell. | | | | | |
| Dysphasia | Awareness of receptive or expressive characteristics; not impairing ability to communicate | Moderate receptive or expressive characteristics; impairing ability to communicate spontaneously | Severe receptive or expressive characteristics; impairing ability to read, write or communicate intelligibly | - | - |
| Definition: A disorder characterized by impairment of verbal communication skills, often resulting from brain damage. | | | | | |
| Edema cerebral | - | - | - | Life-threatening consequences; urgent intervention indicated | - |
| Definition: A disorder characterized by swelling due to an excessive accumulation of fluid in the brain. | | | | | |
| Encephalopathy | Mild symptoms | Moderate symptoms; limiting instrumental ADL | Severe symptoms; limiting self care ADL | Life-threatening consequences; urgent intervention indicated | Death |
| Definition: A disorder characterized by a pathologic process involving the brain. | | | | | |

| **Nervous system disorders** | | | | | |
| --- | --- | --- | --- | --- | --- |
|  | **Grade** | | | | |
| **Adverse Event** | **1** | **2** | **3** | **4** | **5** |
| Extrapyramidal disorder | Mild involuntary movements | Moderate involuntary movements; limiting instrumental ADL | Severe involuntary movements or torticollis; limiting self care ADL | Life-threatening consequences; urgent intervention indicated | Death |
| Definition: A disorder characterized by abnormal, repetitive, involuntary muscle movements, frenzied speech and extreme restlessness. | | | | | |
| Facial muscle weakness | Asymptomatic; clinical or diagnostic observations only; intervention not indicated | Moderate symptoms; limiting instrumental ADL | Severe symptoms; limiting self care ADL | - | - |
| Definition: A disorder characterized by a reduction in the strength of the facial muscles. | | | | | |
| Facial nerve disorder | Asymptomatic; clinical or diagnostic observations only; intervention not indicated | Moderate symptoms; limiting instrumental ADL | Severe symptoms; limiting self care ADL | - | - |
| Definition: A disorder characterized by involvement of the facial nerve (seventh cranial nerve). | | | | | |
| Glossopharyngeal nerve disorder | Asymptomatic; clinical or diagnostic observations only; intervention not indicated | Moderate symptoms; limiting instrumental ADL | Severe symptoms; limiting self care ADL | Life-threatening consequences; urgent intervention indicated | Death |
| Definition: A disorder characterized by involvement of the glossopharyngeal nerve (ninth cranial nerve). | | | | | |
| Headache | Mild pain | Moderate pain; limiting instrumental ADL | Severe pain; limiting self care ADL | - | - |
| Definition: A disorder characterized by a sensation of marked discomfort in various parts of the head, not confined to the area of distribution of any nerve. | | | | | |
| Hydrocephalus | Asymptomatic; clinical or diagnostic observations only; intervention not indicated | Moderate symptoms; intervention not indicated | Severe symptoms or neurological deficit; intervention indicated | Life-threatening consequences; urgent intervention indicated | Death |

| **Nervous system disorders** | | | | | |
| --- | --- | --- | --- | --- | --- |
|  | **Grade** | | | | |
| **Adverse Event** | **1** | **2** | **3** | **4** | **5** |
| Definition: A disorder characterized by an abnormal increase of cerebrospinal fluid in the ventricles of the brain. | | | | | |
| Hypersomnia | Mild increased need for sleep | Moderate increased need for sleep | Severe increased need for sleep | - | - |
| Definition: A disorder characterized by characterized by excessive sleepiness during the daytime. | | | | | |
| Hypoglossal nerve disorder | Asymptomatic; clinical or diagnostic observations only; intervention not indicated | Moderate symptoms; limiting instrumental ADL | Severe symptoms; limiting self care ADL | - | - |
| Definition: A disorder characterized by involvement of the hypoglossal nerve (twelfth cranial nerve). | | | | | |
| Intracranial hemorrhage | Asymptomatic; clinical or diagnostic observations only; intervention not indicated | Moderate symptoms; medical intervention indicated | Ventriculostomy, ICP monitoring, intraventricular thrombolysis, or operative intervention indicated | Life-threatening consequences; urgent intervention indicated | Death |
| Definition: A disorder characterized by bleeding from the cranium. | | | | | |
| Ischemia cerebrovascular | Asymptomatic; clinical or diagnostic observations only; intervention not indicated | Moderate symptoms | - | - | - |
| Definition: A disorder characterized by a decrease or absence of blood supply to the brain caused by obstruction (thrombosis or embolism) of an artery resulting in neurological damage. | | | | | |
| IVth nerve disorder | Asymptomatic; clinical or diagnostic observations only; intervention not indicated | Moderate symptoms; limiting instrumental ADL | Severe symptoms; limiting self care ADL | - | - |
| Definition: A disorder characterized by involvement of the trochlear nerve (fourth cranial nerve). | | | | | |

| **Nervous system disorders** | | | | | |
| --- | --- | --- | --- | --- | --- |
|  | **Grade** | | | | |
| **Adverse Event** | **1** | **2** | **3** | **4** | **5** |
| Lethargy | Mild symptoms; reduced alertness and awareness | Moderate symptoms; limiting instrumental ADL | - | - | - |
| Definition: A disorder characterized by a decrease in consciousness characterized by mental and physical inertness. | | | | | |
| Leukoencephalopathy | Asymptomatic; small focal T2/FLAIR hyperintensities; involving periventricular white matter or <1/3 of susceptible areas of cerebrum +/- mild increase in subarachnoid space (SAS) and/or mild ventriculomegaly | Moderate symptoms; focal T2/FLAIR hyperintensities, involving periventricular white matter extending into centrum semiovale or involving 1/3 to 2/3 of susceptible areas of cerebrum +/- moderate increase in SAS and/or moderate ventriculomegaly | Severe symptoms; extensive T2/FLAIR hyperintensities, involving periventricular white matter involving 2/3 or more of susceptible areas of cerebrum  +/- moderate to severe increase in SAS and/or moderate to severe ventriculomegaly | Life-threatening consequences; extensive T2/FLAIR hyperintensities, involving periventricular white matter involving most of susceptible areas of cerebrum  +/- moderate to severe increase in SAS and/or moderate to severe  ventriculomegaly | Death |
| Definition: A disorder characterized by diffuse reactive astrocytosis with multiple areas of necrotic foci without inflammation. | | | | | |
| Memory impairment | Mild memory impairment | Moderate memory impairment; limiting instrumental ADL | Severe memory impairment; limiting self care ADL | - | - |
| Definition: A disorder characterized by a deterioration in memory function. | | | | | |
| Meningismus | Mild symptoms | Moderate symptoms; limiting instrumental ADL | Severe symptoms; limiting self care ADL | Life-threatening consequences; urgent intervention indicated | Death |
| Definition: A disorder characterized by neck stiffness, headache, and photophobia resulting from irritation of the cerebral meninges. | | | | | |

| **Nervous system disorders** | | | | | |
| --- | --- | --- | --- | --- | --- |
|  | **Grade** | | | | |
| **Adverse Event** | **1** | **2** | **3** | **4** | **5** |
| Movements involuntary | Mild symptoms | Moderate symptoms; limiting instrumental ADL | Severe symptoms; limiting self care ADL | - | - |
| Definition: A disorder characterized by uncontrolled and purposeless movements. | | | | | |
| Myelitis | Asymptomatic; mild signs (e.g., Babinski's reflex or Lhermitte's sign) | Moderate weakness or sensory loss; limiting instrumental ADL | Severe weakness or sensory loss; limiting self care ADL | Life-threatening consequences; urgent intervention indicated | Death |
| Definition: A disorder characterized by inflammation involving the spinal cord. Symptoms include weakness, paresthesia, sensory loss, marked discomfort and incontinence. | | | | | |
| Neuralgia | Mild pain | Moderate pain; limiting instrumental ADL | Severe pain; limiting self care ADL | - | - |
| Definition: A disorder characterized by intense painful sensation along a nerve or group of nerves. | | | | | |
| Nystagmus | - | Moderate symptoms; limiting instrumental ADL | Severe symptoms; limiting self care ADL | - | - |
| Definition: A disorder characterized by involuntary movements of the eyeballs. | | | | | |
| Oculomotor nerve disorder | Asymptomatic; clinical or diagnostic observations only; intervention not indicated | Moderate symptoms; limiting instrumental ADL | Severe symptoms; limiting self care ADL | - | - |
| Definition: A disorder characterized by involvement of the oculomotor nerve (third cranial nerve). | | | | | |
| Olfactory nerve disorder | - | Moderate symptoms; limiting instrumental ADL | Severe symptoms; limiting self care ADL | - | - |
| Definition: A disorder characterized by involvement of the olfactory nerve (first cranial nerve). | | | | | |
| Paresthesia | Mild symptoms | Moderate symptoms; limiting instrumental ADL | Severe symptoms; limiting self care ADL | - | - |

| **Nervous system disorders** | | | | | |
| --- | --- | --- | --- | --- | --- |
|  | **Grade** | | | | |
| **Adverse Event** | **1** | **2** | **3** | **4** | **5** |
| Definition: A disorder characterized by functional disturbances of sensory neurons resulting in abnormal cutaneous sensations of tingling, numbness, pressure, cold, and warmth that are experienced in the absence of a stimulus. | | | | | |
| Peripheral motor neuropathy | Asymptomatic; clinical or diagnostic observations only; intervention not indicated | Moderate symptoms; limiting instrumental ADL | Severe symptoms; limiting self care ADL; assistive device indicated | Life-threatening consequences; urgent intervention indicated | Death |
| Definition: A disorder characterized by inflammation or degeneration of the peripheral motor nerves. | | | | | |
| Peripheral sensory neuropathy | Asymptomatic; loss of deep tendon reflexes or paresthesia | Moderate symptoms; limiting instrumental ADL | Severe symptoms; limiting self care ADL | Life-threatening consequences; urgent intervention indicated | Death |
| Definition: A disorder characterized by inflammation or degeneration of the peripheral sensory nerves. | | | | | |
| Phantom pain | Mild pain | Moderate pain; limiting instrumental ADL | Severe pain; limiting self care ADL | - | - |
| Definition: A disorder characterized by marked discomfort related to a limb or an organ that is removed from or is not physically part of the body. | | | | | |
| Presyncope | - | Present (e.g., near fainting) | - | - | - |
| Definition: A disorder characterized by an episode of lightheadedness and dizziness which may precede an episode of syncope. | | | | | |
| Pyramidal tract syndrome | Asymptomatic; clinical or diagnostic observations only; intervention not indicated | Moderate symptoms; limiting instrumental ADL | Severe symptoms; limiting self care ADL | Life-threatening consequences; urgent intervention indicated | Death |
| Definition: A disorder characterized by dysfunction of the corticospinal (pyramidal) tracts of the spinal cord. Symptoms include an increase in the muscle tone in the lower extremities, hyperreflexia, positive Babinski and a decrease in fine motor coordination. | | | | | |

| **Nervous system disorders** | | | | | |
| --- | --- | --- | --- | --- | --- |
|  | **Grade** | | | | |
| **Adverse Event** | **1** | **2** | **3** | **4** | **5** |
| Radiculitis | Mild symptoms | Moderate symptoms; limiting instrumental ADL; medical intervention indicated | Severe symptoms; limiting self care ADL | Life-threatening consequences; urgent intervention indicated | Death |
| Definition: A disorder characterized by inflammation involving a nerve root. Patients experience marked discomfort radiating along a nerve path because of spinal pressure on the connecting nerve root. | | | | | |
| Recurrent laryngeal nerve palsy | Asymptomatic; clinical or diagnostic observations only; intervention not indicated | Moderate symptoms | Severe symptoms; medical intervention indicated (e.g., thyroplasty, vocal cord injection) | Life-threatening consequences; urgent intervention indicated | Death |
| Definition: A disorder characterized by paralysis of the recurrent laryngeal nerve. | | | | | |
| Reversible posterior leukoencephalopathy syndrome | Asymptomatic; clinical or diagnostic observations only; intervention not indicated | Moderate symptoms; abnormal imaging studies; limiting instrumental ADL | Severe symptoms; very abnormal imaging studies; limiting self care ADL | Life-threatening consequences; urgent intervention indicated | Death |
| Definition: A disorder characterized by headaches, mental status changes, visual disturbances, and seizures associated with imaging findings of posterior leukoencephalopathy. It has been observed in association with hypertensive encephalopathy, eclampsia, and immunosuppressive and cytotoxic drug treatment. It is an acute or subacute reversible condition. | | | | | |
| Seizure | Brief partial seizure; no loss of consciousness | Brief generalized seizure | Multiple seizures despite medical intervention | Life-threatening; prolonged repetitive seizures | Death |
| Definition: A disorder characterized by a sudden, involuntary skeletal muscular contractions of cerebral or brain stem origin. | | | | | |
| Sinus pain | Mild pain | Moderate pain; limiting instrumental ADL | Severe pain; limiting self care ADL | - | - |
| Definition: A disorder characterized by marked discomfort in the face, between the eyes, or upper teeth originating from the sinuses. | | | | | |

| **Nervous system disorders** | | | | | |
| --- | --- | --- | --- | --- | --- |
|  | **Grade** | | | | |
| **Adverse Event** | **1** | **2** | **3** | **4** | **5** |
| Somnolence | Mild but more than usual drowsiness or sleepiness | Moderate sedation; limiting instrumental ADL | Obtundation or stupor | Life-threatening consequences; urgent intervention indicated | Death |
| Definition: A disorder characterized by characterized by excessive sleepiness and drowsiness. | | | | | |
| Spasticity | Mild or slight increase in muscle tone | Moderate increase in muscle tone and increase in resistance through range of motion | Severe increase in muscle tone and increase in resistance through range of motion | Life-threatening; unable to move active or passive range of motion | Death |
| Definition: A disorder characterized by increased involuntary muscle tone that affects the regions interfering with voluntary movement. It results in gait, movement, and speech disturbances. | | | | | |
| Stroke | Asymptomatic or mild neurologic deficit; radiographic findings only | Moderate neurologic deficit | Severe neurologic deficit | Life-threatening consequences; urgent intervention indicated | Death |
| Definition: A disorder characterized by a sudden loss of sensory function due to an intracranial vascular event. | | | | | |
| Syncope | - | - | Fainting; orthostatic collapse | - | - |
| Definition: A disorder characterized by spontaneous loss of consciousness caused by insufficient blood supply to the brain. | | | | | |
| Transient ischemic attacks | Mild neurologic deficit with or without imaging confirmation | Moderate neurologic deficit with or without imaging confirmation | - | - | - |
| Definition: A disorder characterized by a brief attack (less than 24 hours) of cerebral dysfunction of vascular origin, with no persistent neurological deficit. | | | | | |
| Tremor | Mild symptoms | Moderate symptoms; limiting instrumental ADL | Severe symptoms; limiting self care ADL | - | - |

| **Nervous system disorders** | | | | | |
| --- | --- | --- | --- | --- | --- |
|  | **Grade** | | | | |
| **Adverse Event** | **1** | **2** | **3** | **4** | **5** |
| Definition: A disorder characterized by the uncontrolled shaking movement of the whole body or individual parts. | | | | | |
| Trigeminal nerve disorder | Asymptomatic; clinical or diagnostic observations only; intervention not indicated | Moderate symptoms; limiting instrumental ADL | Severe symptoms; limiting self care ADL | - | - |
| Definition: A disorder characterized by involvement of the trigeminal nerve (fifth cranial nerve). | | | | | |
| Vagus nerve disorder | Asymptomatic; clinical or diagnostic observations only; intervention not indicated | Moderate symptoms; limiting instrumental ADL | Severe symptoms; limiting self care ADL | Life-threatening consequences; urgent intervention indicated | Death |
| Definition: A disorder characterized by involvement of the vagus nerve (tenth cranial nerve). | | | | | |
| Vasovagal reaction | - | - | Present | Life-threatening consequences; urgent intervention indicated | Death |
| Definition: A disorder characterized by a sudden drop of the blood pressure, bradycardia, and peripheral vasodilation that may lead to loss of consciousness. It results from an increase in the stimulation of the vagus nerve. | | | | | |
| Nervous system disorders - Other, specify | Asymptomatic or mild symptoms; clinical or diagnostic observations only; intervention not indicated | Moderate; minimal, local or noninvasive intervention indicated; limiting age- appropriate instrumental ADL | Severe or medically significant but not immediately life- threatening; hospitalization or prolongation of existing hospitalization indicated; disabling; limiting self care ADL | Life-threatening consequences; urgent intervention indicated | Death |

| **Respiratory, thoracic and mediastinal disorders** | | | | | |
| --- | --- | --- | --- | --- | --- |
|  | **Grade** | | | | |
| **Adverse Event** | **1** | **2** | **3** | **4** | **5** |
| Adult respiratory distress syndrome | - | - | Present with radiologic findings; intubation not indicated | Life-threatening respiratory or hemodynamic compromise; intubation or urgent intervention indicated | Death |
| Definition: A disorder characterized by progressive and life-threatening pulmonary distress in the absence of an underlying pulmonary condition, usually following major trauma or surgery. | | | | | |
| Allergic rhinitis | Mild symptoms; intervention not indicated | Moderate symptoms; medical intervention indicated | - | - | - |
| Definition: A disorder characterized by an inflammation of the nasal mucous membranes caused by an IgE-mediated response to external allergens. The inflammation may also involve the mucous membranes of the sinuses, eyes, middle ear, and pharynx. Symptoms include sneezing, nasal congestion, rhinorrhea and itching. | | | | | |
| Apnea | - | - | Present; medical intervention indicated | Life-threatening respiratory or hemodynamic compromise; intubation or urgent intervention indicated | Death |
| Definition: A disorder characterized by cessation of breathing. | | | | | |
| Aspiration | Asymptomatic; clinical or diagnostic observations only; intervention not indicated | Altered eating habits; coughing or choking episodes after eating or swallowing; medical intervention indicated (e.g., suction or oxygen) | Dyspnea and pneumonia symptoms (e.g., aspiration pneumonia); hospitalization indicated; unable to aliment orally | Life-threatening respiratory or hemodynamic compromise; intubation or urgent intervention indicated | Death |
| Definition: A disorder characterized by inhalation of solids or liquids into the lungs. | | | | | |

| **Respiratory, thoracic and mediastinal disorders** | | | | | |
| --- | --- | --- | --- | --- | --- |
|  | **Grade** | | | | |
| **Adverse Event** | **1** | **2** | **3** | **4** | **5** |
| Atelectasis | Asymptomatic; clinical or diagnostic observations only; intervention not indicated | Symptomatic (e.g., dyspnea, cough); medical intervention indicated (e.g., chest physiotherapy, suctioning); bronchoscopic suctioning | Oxygen indicated; hospitalization or elective operative intervention indicated (e.g., stent, laser) | Life-threatening respiratory or hemodynamic compromise; intubation or urgent intervention indicated | Death |
| Definition: A disorder characterized by the collapse of part or the entire lung. | | | | | |
| Bronchial fistula | Asymptomatic; clinical or diagnostic observations only; intervention not indicated | Symptomatic; tube thoracostomy or medical management indicated; limiting instrumental ADL | Severe symptoms; limiting self care ADL; endoscopic or operative intervention indicated (e.g., stent or primary closure) | Life-threatening consequences; urgent operative intervention with thoracoplasty, chronic open drainage or multiple thoracotomies indicated | Death |
| Definition: A disorder characterized by an abnormal communication between the bronchus and another organ or anatomic site. | | | | | |
| Bronchial obstruction | Asymptomatic; clinical or diagnostic observations only; intervention not indicated | Symptomatic (e.g., mild wheezing); endoscopic evaluation indicated; radiographic evidence of atelectasis/lobar collapse; medical management indicated (e.g., steroids, bronchodilators) | Shortness of breath with stridor; endoscopic intervention indicated (e.g., laser, stent placement) | Life-threatening respiratory or hemodynamic compromise; intubation or urgent intervention indicated | Death |
| Definition: A disorder characterized by blockage of a bronchus passage, most often by bronchial secretions and exudates. | | | | | |

| **Respiratory, thoracic and mediastinal disorders** | | | | | |
| --- | --- | --- | --- | --- | --- |
|  | **Grade** | | | | |
| **Adverse Event** | **1** | **2** | **3** | **4** | **5** |
| Bronchial stricture | Asymptomatic; clinical or diagnostic observations only; intervention not indicated | Symptomatic (e.g., rhonchi or wheezing) but without respiratory distress; medical intervention indicated (e.g., steroids, bronchodilators) | Shortness of breath with stridor; endoscopic intervention indicated (e.g., laser, stent placement) | Life-threatening respiratory or hemodynamic compromise; intubation or urgent intervention indicated | Death |
| Definition: A disorder characterized by a narrowing of the bronchial tube. | | | | | |
| Bronchopleural fistula | Asymptomatic; clinical or diagnostic observations only; intervention not indicated | Symptomatic; tube thoracostomy or medical intervention indicated; limiting instrumental ADL | Severe symptoms; limiting self care ADL; endoscopic or operative intervention indicated (e.g., stent or primary closure) | Life-threatening consequences; urgent operative intervention with thoracoplasty, chronic open drainage or multiple thoracotomies indicated | Death |
| Definition: A disorder characterized by an abnormal communication between a bronchus and the pleural cavity. | | | | | |
| Bronchopulmonary hemorrhage | Mild symptoms; intervention not indicated | Moderate symptoms; medical intervention indicated | Transfusion, radiologic, endoscopic, or operative intervention indicated (e.g., hemostasis of bleeding site) | Life-threatening respiratory or hemodynamic compromise; intubation or urgent intervention indicated | Death |
| Definition: A disorder characterized by bleeding from the bronchial wall and/or lung parenchyma. | | | | | |
| Bronchospasm | Mild symptoms; intervention not indicated | Symptomatic; medical intervention indicated; limiting instrumental ADL | Limiting self care ADL; oxygen saturation decreased | Life-threatening respiratory or hemodynamic compromise; intubation or urgent intervention indicated | Death |
| Definition: A disorder characterized by a sudden contraction of the smooth muscles of the bronchial wall. | | | | | |

| **Respiratory, thoracic and mediastinal disorders** | | | | | |
| --- | --- | --- | --- | --- | --- |
|  | **Grade** | | | | |
| **Adverse Event** | **1** | **2** | **3** | **4** | **5** |
| Chylothorax | Asymptomatic; clinical or diagnostic observations only; intervention not indicated | Symptomatic; thoracentesis or tube drainage indicated | Severe symptoms; elective operative intervention indicated | Life-threatening respiratory or hemodynamic compromise; intubation or urgent intervention indicated | Death |
| Definition: A disorder characterized by milky pleural effusion (abnormal collection of fluid) resulting from accumulation of lymph fluid in the pleural cavity. | | | | | |
| Cough | Mild symptoms; nonprescription intervention indicated | Moderate symptoms, medical intervention indicated; limiting instrumental ADL | Severe symptoms; limiting self care ADL | - | - |
| Definition: A disorder characterized by sudden, often repetitive, spasmodic contraction of the thoracic cavity, resulting in violent release of air from the lungs and usually accompanied by a distinctive sound. | | | | | |
| Dyspnea | Shortness of breath with moderate exertion | Shortness of breath with minimal exertion; limiting instrumental ADL | Shortness of breath at rest; limiting self care ADL | Life-threatening consequences; urgent intervention indicated | Death |
| Definition: A disorder characterized by an uncomfortable sensation of difficulty breathing. | | | | | |
| Epistaxis | Mild symptoms; intervention not indicated | Moderate symptoms; medical intervention indicated (e.g., nasal packing, cauterization; topical vasoconstrictors) | Transfusion, radiologic, endoscopic, or operative intervention indicated (e.g., hemostasis of bleeding site) | Life-threatening consequences; urgent intervention indicated | Death |
| Definition: A disorder characterized by bleeding from the nose. | | | | | |
| Hiccups | Mild symptoms; intervention not indicated | Moderate symptoms; medical intervention indicated; limiting instrumental ADL | Severe symptoms; interfering with sleep; limiting self care ADL | - | - |
| Definition: A disorder characterized by repeated gulp sounds that result from an involuntary opening and closing of the glottis. This is attributed to a spasm of the diaphragm. | | | | | |

| **Respiratory, thoracic and mediastinal disorders** | | | | | |
| --- | --- | --- | --- | --- | --- |
|  | **Grade** | | | | |
| **Adverse Event** | **1** | **2** | **3** | **4** | **5** |
| Hoarseness | Mild or intermittent voice change; fully understandable; self-resolves | Moderate or persistent voice changes; may require occasional repetition but understandable on telephone; medical evaluation indicated | Severe voice changes including predominantly whispered speech | - | - |
| Definition: A disorder characterized by harsh and raspy voice arising from or spreading to the larynx. | | | | | |
| Hypoxia | - | Decreased oxygen saturation with exercise (e.g., pulse oximeter <88%); intermittent supplemental oxygen | Decreased oxygen saturation at rest (e.g., pulse oximeter  <88% or PaO2 <=55 mm Hg) | Life-threatening airway compromise; urgent intervention indicated (e.g., tracheotomy or intubation) | Death |
| Definition: A disorder characterized by a decrease in the level of oxygen in the body. | | | | | |
| Laryngeal edema | Asymptomatic; clinical or diagnostic observations only; intervention not indicated | Symptomatic; medical intervention indicated (e.g., dexamethasone, epinephrine, antihistamines) | Stridor; respiratory distress; hospitalization indicated | Life-threatening airway compromise; urgent intervention indicated (e.g., tracheotomy or intubation) | Death |
| Definition: A disorder characterized by swelling due to an excessive accumulation of fluid in the larynx. | | | | | |
| Laryngeal fistula | Asymptomatic; clinical or diagnostic observations only; intervention not indicated | Symptomatic; tube thoracostomy or medical management indicated; limiting instrumental ADL | Severe symptoms; limiting self care ADL; endoscopic or operative intervention indicated (e.g., stent or primary closure) | Life-threatening consequences; urgent operative intervention indicated (e.g., thoracoplasty, chronic open drainage or multiple thoracotomies) | Death |
| Definition: A disorder characterized by an abnormal communication between the larynx and another organ or anatomic site. | | | | | |

| **Respiratory, thoracic and mediastinal disorders** | | | | | |
| --- | --- | --- | --- | --- | --- |
|  | **Grade** | | | | |
| **Adverse Event** | **1** | **2** | **3** | **4** | **5** |
| Laryngeal hemorrhage | Mild cough or trace hemoptysis; laryngoscopic findings | Moderate symptoms; medical intervention indicated | Transfusion, radiologic, endoscopic, or operative intervention indicated (e.g., hemostasis of bleeding site) | Life-threatening airway compromise; urgent intervention indicated (e.g., tracheotomy or intubation) | Death |
| Definition: A disorder characterized by bleeding from the larynx. | | | | | |
| Laryngeal inflammation | Mild sore throat; raspy voice | Moderate sore throat; analgesics indicated | Severe throat pain; endoscopic intervention indicated | - | - |
| Definition: A disorder characterized by an inflammation involving the larynx. | | | | | |
| Laryngeal mucositis | Endoscopic findings only; mild discomfort with normal intake | Moderate discomfort; altered oral intake | Severe pain; severely altered eating/swallowing; medical intervention indicated | Life-threatening airway compromise; urgent intervention indicated (e.g., tracheotomy or intubation) | Death |
| Definition: A disorder characterized by an inflammation involving the mucous membrane of the larynx. | | | | | |
| Laryngeal obstruction | Asymptomatic; clinical or diagnostic observations only; intervention not indicated | Symptomatic (e.g., noisy airway breathing), but causing no respiratory distress; medical management indicated (e.g., steroids); limiting instrumental ADL | Limiting self care ADL; stridor; endoscopic intervention indicated (e.g., stent, laser) | Life-threatening consequences; urgent intervention indicated | Death |
| Definition: A disorder characterized by blockage of the laryngeal airway. | | | | | |

| **Respiratory, thoracic and mediastinal disorders** | | | | | |
| --- | --- | --- | --- | --- | --- |
|  | **Grade** | | | | |
| **Adverse Event** | **1** | **2** | **3** | **4** | **5** |
| Laryngeal stenosis | Asymptomatic; clinical or diagnostic observations only; intervention not indicated | Symptomatic (e.g., noisy airway breathing), but causing no respiratory distress; medical management indicated (e.g., steroids) | Limiting self care ADL; stridor; endoscopic intervention indicated (e.g., stent, laser) | Life-threatening consequences; urgent intervention indicated | Death |
| Definition: A disorder characterized by a narrowing of the laryngeal airway. | | | | | |
| Laryngopharyngeal dysesthesia | Mild symptoms; no anxiety; intervention not indicated | Moderate symptoms; mild anxiety, but no dyspnea; short duration of observation and or anxiolytic indicated; limiting instrumental ADL | Severe symptoms; dyspnea and swallowing difficulty; limiting self care ADL | Life-threatening consequences | Death |
| Definition: A disorder characterized by an uncomfortable persistent sensation in the area of the laryngopharynx. | | | | | |
| Laryngospasm | - | Transient episode; intervention not indicated | Recurrent episodes; noninvasive intervention indicated (e.g., breathing technique, pressure point massage) | Persistent or severe episodes associated with syncope; urgent intervention indicated (e.g., fiberoptic laryngoscopy, intubation, botox injection) | Death |
| Definition: A disorder characterized by paroxysmal spasmodic muscular contraction of the vocal cords. | | | | | |
| Mediastinal hemorrhage | Radiologic evidence only; minimal symptoms; intervention not indicated | Moderate symptoms; medical intervention indicated | Transfusion, radiologic, endoscopic, or elective operative intervention indicated (e.g., hemostasis of bleeding site) | Life-threatening consequences; urgent intervention indicated | Death |

| **Respiratory, thoracic and mediastinal disorders** | | | | | |
| --- | --- | --- | --- | --- | --- |
|  | **Grade** | | | | |
| **Adverse Event** | **1** | **2** | **3** | **4** | **5** |
| Definition: A disorder characterized by bleeding from the mediastinum. | | | | | |
| Nasal congestion | Mild symptoms; intervention not indicated | Moderate symptoms; medical intervention indicated | Associated with bloody nasal discharge or epistaxis | - | - |
| Definition: A disorder characterized by obstruction of the nasal passage due to mucosal edema. | | | | | |
| Pharyngeal fistula | Asymptomatic; clinical or diagnostic observations only; intervention not indicated | Symptomatic; tube thoracostomy or medical intervention indicated; limiting instrumental ADL | Severe symptoms; limiting self care ADL; endoscopic or operative intervention indicated (e.g., stent or primary closure) | Life-threatening consequences; urgent intervention indicated | Death |
| Definition: A disorder characterized by an abnormal communication between the pharynx and another organ or anatomic site. | | | | | |
| Pharyngeal hemorrhage | Mild symptoms; intervention not indicated | Moderate symptoms; medical intervention indicated | Transfusion, radiologic, endoscopic, or operative intervention indicated (e.g., hemostasis of bleeding site) | Life-threatening respiratory or hemodynamic compromise; intubation or urgent intervention indicated | Death |
| Definition: A disorder characterized by bleeding from the pharynx. | | | | | |
| Pharyngeal mucositis | Endoscopic findings only; minimal symptoms with normal oral intake; mild pain but analgesics not indicated | Moderate pain and analgesics indicated; altered oral intake; limiting instrumental ADL | Severe pain; unable to adequately aliment or hydrate orally; limiting self care ADL | Life-threatening consequences; urgent intervention indicated | Death |
| Definition: A disorder characterized by an inflammation involving the mucous membrane of the pharynx. | | | | | |

| **Respiratory, thoracic and mediastinal disorders** | | | | | |
| --- | --- | --- | --- | --- | --- |
|  | **Grade** | | | | |
| **Adverse Event** | **1** | **2** | **3** | **4** | **5** |
| Pharyngeal necrosis | - | - | Inability to aliment adequately by GI tract; tube feeding or TPN indicated; radiologic, endoscopic, or operative intervention indicated | Life-threatening consequences; urgent operative intervention indicated | Death |
| Definition: A disorder characterized by a necrotic process occurring in the pharynx. | | | | | |
| Pharyngeal stenosis | Asymptomatic; clinical or diagnostic observations only; intervention not indicated | Symptomatic (e.g., noisy airway breathing), but causing no respiratory distress; medical management indicated (e.g., steroids); limiting instrumental ADL | Limiting self care ADL; stridor; endoscopic intervention indicated (e.g., stent, laser) | Life-threatening airway compromise; urgent intervention indicated (e.g., tracheotomy or intubation) | Death |
| Definition: A disorder characterized by a narrowing of the pharyngeal airway. | | | | | |
| Pharyngolaryngeal pain | Mild pain | Moderate pain; limiting instrumental ADL | Severe pain; limiting self care ADL | - | - |
| Definition: A disorder characterized by marked discomfort sensation in the pharyngolaryngeal region. | | | | | |
| Pleural effusion | Asymptomatic; clinical or diagnostic observations only; intervention not indicated | Symptomatic; intervention indicated (e.g., diuretics or limited therapeutic thoracentesis) | Symptomatic with respiratory distress and hypoxia; surgical intervention including chest tube or pleurodesis indicated | Life-threatening respiratory or hemodynamic compromise; intubation or urgent intervention indicated | Death |
| Definition: A disorder characterized by an increase in amounts of fluid within the pleural cavity. Symptoms include shortness of breath, cough and marked chest discomfort. | | | | | |

| **Respiratory, thoracic and mediastinal disorders** | | | | | |
| --- | --- | --- | --- | --- | --- |
|  | **Grade** | | | | |
| **Adverse Event** | **1** | **2** | **3** | **4** | **5** |
| Pleural hemorrhage | Asymptomatic; mild hemorrhage confirmed by thoracentesis | Symptomatic or associated with pneumothorax; chest tube drainage indicated | >1000 ml of blood evacuated; persistent bleeding (150-200 ml/hr for 2 - 4 hr); persistent transfusion indicated; elective operative intervention indicated | Life-threatening respiratory or hemodynamic compromise; intubation or urgent intervention indicated | Death |
| Definition: A disorder characterized by bleeding from the pleural cavity. | | | | | |
| Pleuritic pain | Mild pain | Moderate pain; limiting instrumental ADL | Severe pain; limiting self care ADL | - | - |
| Definition: A disorder characterized by marked discomfort sensation in the pleura. | | | | | |
| Pneumonitis | Asymptomatic; clinical or diagnostic observations only; intervention not indicated | Symptomatic; medical intervention indicated; limiting instrumental ADL | Severe symptoms; limiting self care ADL; oxygen indicated | Life-threatening respiratory compromise; urgent intervention indicated (e.g., tracheotomy or intubation) | Death |
| Definition: A disorder characterized by inflammation focally or diffusely affecting the lung parenchyma. | | | | | |
| Pneumothorax | Asymptomatic; clinical or diagnostic observations only; intervention not indicated | Symptomatic; intervention indicated (e.g., tube placement without sclerosis) | Sclerosis and/or operative intervention indicated; hospitalization indicated | Life-threatening consequences; urgent intervention indicated | Death |
| Definition: A disorder characterized by abnormal presence of air in the pleural cavity resulting in the collapse of the lung. | | | | | |
| Postnasal drip | Mild symptoms; intervention not indicated | Moderate symptoms; medical intervention indicated | - | - | - |
| Definition: A disorder characterized by excessive mucous secretion in the back of the nasal cavity or throat, causing sore throat and/or coughing. | | | | | |

| **Respiratory, thoracic and mediastinal disorders** | | | | | |
| --- | --- | --- | --- | --- | --- |
|  | **Grade** | | | | |
| **Adverse Event** | **1** | **2** | **3** | **4** | **5** |
| Productive cough | Occasional/minimal production of sputum with cough | Moderate sputum production; limiting instrumental ADL | Persistent or copious production of sputum; limiting self care ADL | - | - |
| Definition: A disorder characterized by expectorated secretions upon coughing. | | | | | |
| Pulmonary edema | Radiologic findings only; minimal dyspnea on exertion | Moderate dyspnea on exertion; medical intervention indicated; limiting instrumental ADL | Severe dyspnea or dyspnea at rest; oxygen indicated; limiting self care ADL | Life-threatening respiratory compromise; urgent intervention or intubation with ventilatory support indicated | Death |
| Definition: A disorder characterized by accumulation of fluid in the lung tissues that causes a disturbance of the gas exchange that may lead to respiratory failure. | | | | | |
| Pulmonary fibrosis | Mild hypoxemia; radiologic pulmonary fibrosis <25% of lung volume | Moderate hypoxemia; evidence of pulmonary hypertension; radiographic pulmonary fibrosis 25 - 50% | Severe hypoxemia; evidence of right-sided heart failure; radiographic pulmonary fibrosis >50 - 75% | Life-threatening consequences (e.g., hemodynamic/pulmonary complications); intubation with ventilatory support indicated; radiographic pulmonary fibrosis >75% with severe honeycombing | Death |
| Definition: A disorder characterized by the replacement of the lung tissue by connective tissue, leading to progressive dyspnea, respiratory failure or right heart failure. | | | | | |
| Pulmonary fistula | Asymptomatic; clinical or diagnostic observations only; intervention not indicated | Symptomatic; tube thoracostomy or medical management indicated; limiting instrumental ADL | Limiting self care ADL; endoscopic stenting or operative intervention indicated | Life-threatening consequences; urgent operative intervention indicated | Death |
| Definition: A disorder characterized by an abnormal communication between the lung and another organ or anatomic site. | | | | | |

| **Respiratory, thoracic and mediastinal disorders** | | | | | |
| --- | --- | --- | --- | --- | --- |
|  | **Grade** | | | | |
| **Adverse Event** | **1** | **2** | **3** | **4** | **5** |
| Pulmonary hypertension | Minimal dyspnea; findings on physical exam or other evaluation | Moderate dyspnea, cough; requiring evaluation by cardiac catheterization and medical intervention | Severe symptoms, associated with hypoxemia, right heart failure; oxygen indicated | Life-threatening airway consequences; urgent intervention indicated (e.g., tracheotomy or intubation) | Death |
| Definition: A disorder characterized by an increase in pressure within the pulmonary circulation due to lung or heart disorder. | | | | | |
| Respiratory failure | - | - | - | Life-threatening consequences; urgent intervention, intubation, or ventilatory support indicated | Death |
| Definition: A disorder characterized by impaired gas exchange by the respiratory system resulting in hypoxemia and a decrease in oxygenation of the tissues that may be associated with an increase in arterial levels of carbon dioxide. | | | | | |
| Retinoic acid syndrome | Fluid retention; <3 kg of weight gain; intervention with fluid restriction and/or diuretics indicated | Moderate signs or symptoms; steroids indicated | Severe symptoms; hospitalization indicated | Life-threatening consequences; ventilatory support indicated | Death |
| Definition: A disorder characterized by weight gain, dyspnea, pleural and pericardial effusions, leukocytosis and/or renal failure originally described in patients treated with all-trans retinoic acid. | | | | | |
| Sinus disorder | Asymptomatic mucosal crusting; blood-tinged secretions | Symptomatic stenosis or edema/narrowing interfering with airflow; limiting instrumental ADL | Stenosis with significant nasal obstruction; limiting self care ADL | Necrosis of soft tissue or bone; urgent operative intervention indicated | Death |
| Definition: A disorder characterized by involvement of the paranasal sinuses. | | | | | |

| **Respiratory, thoracic and mediastinal disorders** | | | | | |
| --- | --- | --- | --- | --- | --- |
|  | **Grade** | | | | |
| **Adverse Event** | **1** | **2** | **3** | **4** | **5** |
| Sleep apnea | Snoring and nocturnal sleep arousal without apneic periods | Moderate apnea and oxygen desaturation; excessive daytime sleepiness; medical evaluation indicated; limiting instrumental ADL | Oxygen desaturation; associated with hypertension; medical intervention indicated; limiting self care ADL | Cardiovascular or neuropsychiatric symptoms; urgent operative intervention indicated | Death |
| Definition: A disorder characterized by cessation of breathing for short periods during sleep. | | | | | |
| Sneezing | Mild symptoms; intervention not indicated | Moderate symptoms; medical intervention indicated | - | - | - |
| Definition: A disorder characterized by the involuntary expulsion of air from the nose. | | | | | |
| Sore throat | Mild pain | Moderate pain; limiting instrumental ADL | Severe pain; limiting self care ADL; limiting ability to swallow | - | - |
| Definition: A disorder characterized by of marked discomfort in the throat | | | | | |
| Stridor | - | - | Respiratory distress limiting self care ADL; medical intervention indicated | Life-threatening airway compromise; urgent intervention indicated (e.g., tracheotomy or intubation) | Death |
| Definition: A disorder characterized by a high pitched breathing sound due to laryngeal or upper airway obstruction. | | | | | |
| Tracheal fistula | Asymptomatic; clinical or diagnostic observations only; intervention not indicated | Symptomatic; tube thoracostomy or medical intervention indicated; limiting instrumental ADL | Severe symptoms; limiting self care ADL; endoscopic or operative intervention indicated (e.g., stent or primary closure) | Life-threatening consequences; urgent operative intervention indicated (e.g., thoracoplasty, chronic open drainage or multiple thoracotomies) | Death |

| **Respiratory, thoracic and mediastinal disorders** | | | | | |
| --- | --- | --- | --- | --- | --- |
|  | **Grade** | | | | |
| **Adverse Event** | **1** | **2** | **3** | **4** | **5** |
| Definition: A disorder characterized by an abnormal communication between the trachea and another organ or anatomic site. | | | | | |
| Tracheal mucositis | Endoscopic findings only; minimal hemoptysis, pain, or respiratory symptoms | Moderate symptoms; medical intervention indicated; limiting instrumental ADL | Severe pain; hemorrhage or respiratory symptoms; limiting self care ADL | Life-threatening consequences; urgent intervention indicated | Death |
| Definition: A disorder characterized by an inflammation involving the mucous membrane of the trachea. | | | | | |
| Tracheal stenosis | Asymptomatic; clinical or diagnostic observations only; intervention not indicated | Symptomatic (e.g., noisy airway breathing), but causing no respiratory distress; medical management indicated (e.g., steroids) | Stridor or respiratory distress limiting self care ADL; endoscopic intervention indicated (e.g., stent, laser) | Life-threatening airway compromise; urgent intervention indicated (e.g., tracheotomy or intubation) | Death |
| Definition: A disorder characterized by a narrowing of the trachea. | | | | | |
| Voice alteration | Mild or intermittent change from normal voice | Moderate or persistent change from normal voice; still understandable | Severe voice changes including predominantly whispered speech; may require frequent repetition or face-to-face contact for understandability; may require assistive technology | - | - |
| Definition: A disorder characterized by a change in the sound and/or speed of the voice. | | | | | |
| Wheezing | Detectable airway noise with minimal symptoms | Moderate symptoms; medical intervention indicated; limiting instrumental ADL | Severe respiratory symptoms limiting self care ADL; oxygen therapy or hospitalization indicated | Life-threatening consequences; urgent intervention indicated | Death |

| **Respiratory, thoracic and mediastinal disorders** | | | | | |
| --- | --- | --- | --- | --- | --- |
|  | **Grade** | | | | |
| **Adverse Event** | **1** | **2** | **3** | **4** | **5** |
| Definition: A disorder characterized by a high-pitched, whistling sound during breathing. It results from the narrowing or obstruction of the respiratory airways. | | | | | |
| Respiratory, thoracic and mediastinal disorders - Other, specify | Asymptomatic or mild symptoms; clinical or diagnostic observations only; intervention not indicated | Moderate; minimal, local or noninvasive intervention indicated; limiting age- appropriate instrumental ADL | Severe or medically significant but not immediately life- threatening; hospitalization or prolongation of existing hospitalization indicated; disabling; limiting self care ADL | Life-threatening consequences; urgent intervention indicated | Death |

**APPENDIX IV Response Evaluation Criteria in Solid Tumors**

（Response Evaluation Criteria In Solid Tumors, RECIST criteria version 1.1.）

**1 Measurability of tumor at baseline**

- 1. **Definitions**

At baseline, Tumour lesions/lymph nodes will be categorized as measurable or non-measurable as follows:

**1.1.1 Measurable**

Tumor lesions: Must be accurately measured in at least one dimension (longest diameter in the plane of measurement is to be recorded) with a minimum size of:

- 10 mm by CT scan (CT scan slice thickness no greater than 5 mm; see Appendix II on imaging guidance).
- 10 mm caliper measurement by clinical exam (lesions that cannot be accurately measured with calipers should be recorded as non-measurable).
- 20 mm by chest X-ray.

*Malignant lymph nodes*: To be considered pathologically enlarged *and* measurable, a lymph node must be P15 mm in *short* axis when assessed by CT scan (CT scan slice thickness recommended to be no greater than 5 mm). At baseline and in follow-up, only the *short* axis will be measured and followed (see Schwartz et al. in this Special Issue[^15^](#_bookmark16)). See also notes below on ‘Baseline documentation of target and non-target lesions’ for information on lymph node measurement.

**1.1.2 Non-measurable**

All other lesions, including small lesions (longest diameter

<10 mm or pathological lymph nodes with P10 to <15 mm short axis) as well as truly non-measurable lesions. Lesions considered truly non-measurable include leptomeningeal disease, ascites, pleural or pericardial effusion, inflammatory breast disease, lymphangitic involvement of skin or lung, abdominal masses/abdominal organomegaly identified by a physical examination that is not measurable by reproducible imaging techniques.

1.1.3 *Special considerations regarding lesion measurability* Bone lesions, cystic lesions, and lesions previously treated with local therapy require particular comments:

**Bone lesions:**

- - Bone scans, PET scans or plain films are not considered adequate imaging techniques to measure bone lesions. How- ever, these techniques can be used to confirm the presence or disappearance of bone lesions.
  - Lytic bone lesions or mixed lytic-blastic lesions, with *identi**fiable soft tissue components* that can be evaluated by cross-sectional imaging techniques such as CT or MRI, can be considered as measurable lesions if the *soft tissue component* meets the definition of measurability described above.
  - Blastic bone lesions are non-measurable.

**Cystic lesions:**

- - Lesions that meet the criteria for radiographically defined simple cysts should not be considered as malignant lesions (neither measurable nor non-measurable) since they are, by definition, simple cysts.
  - ‘Cystic lesions’ thought to represent cystic metastases can be considered as measurable lesions if they meet the definition of measurability described above. However, if non-cystic lesions are present in the same patient, these are preferred for selection as target lesions.

**Lesions with prior local treatment:**

Tumour lesions situated in a previously irradiated area or in an area subjected to other loco-regional therapy, are usually not considered measurable unless there has been demonstrated progression in the lesion. Study protocols should detail the conditions under which such lesions would be considered measurable

**1.2 Specifications by methods of measurements**

1.2.1 *Measurement of lesions*

All measurements should be recorded in metric notation, using calipers if clinically assessed. All baseline evaluations should be performed as close as possible to the treatment start and never more than 4 weeks before the beginning of the treatment.

- - 1. **Method of assessment**

The same method of assessment and the same technique should be used to characterize each identified and reported lesion at baseline and during follow-up. Imaging-based evaluation should always be done rather than clinical examination unless the lesion(s) being followed cannot be imaged but are assessable by clinical exam.

*Clinical lesions:* Clinical lesions will only be considered measurable when they are superficial and P10 mm diameter as assessed using calipers (e.g., skin nodules). For the case of skin lesions, documentation by color photography, including a ruler to estimate the size of the lesion is suggested. As noted above, when both clinical examinations and imaging can evaluate lesions, imaging evaluation should be undertaken since it is more objective and may also be reviewed at the end of the study.

*Chest X-ray:* Chest CT is preferred over chest X-ray, particularly when progression is an important end-point, since CT is more sensitive than X-ray, particularly in identifying new lesions. However, lesions on chest X-ray may be considered measurable if they are clearly defined and surrounded by aerated lung. See Appendix II for more details.

*CT, MRI:* CT is the best currently available and reproducible method to measure lesions selected for response assessment. This guideline has defined the measurability of lesions on CT scans based on the assumption that CT slice thickness is 5 mm or less. As is described in Appendix II, when CT scans have a slice thickness greater than 5 mm, the minimum size for a measurable lesion should be twice the slice thickness. MRI is also acceptable in certain situations (e.g., for body scans). More details concerning the use of both CT and MRI for the assessment of objective tumor response evaluation are provided in Appendix II.

*Ultrasound:* Ultrasound is not useful in the assessment of lesion size and should not be used as a method of measurement. Ultrasound examinations cannot be reproduced in their entirety for an independent review at a later date, and because they are operator dependent, it cannot be guaranteed that the same technique and measurements will be taken from one assessment to the next (described in greater detail in Appendix II). If new lesions are identified by ultrasound in the course of the study, confirmation by CT or MRI is advised. If there is concern about radiation exposure at CT, MRI may be used instead of CT in selected instances.

*Endoscopy and laparoscopy:* The utilization of these techniques for objective tumor evaluation is not advised. However, they can be useful to confirm complete pathological response when biopsies are obtained or to determine relapse in trials where recurrence following complete response or surgical resection is an end-point.

*Tumor markers:* Tumour markers alone cannot be used to assess objective tumor response. If markers are initially above

the upper normal limit, however, they must normalize for a patient to be considered in complete response. Because tumor markers are disease-specific, instructions for their measurement should be incorporated into protocols on a disease-specific basis. Specific guidelines for both CA-125 response (in recurrent ovarian cancer) and PSA response (in recurrent prostate cancer) have been published.[^16–18^](#_bookmark16) In addition, the Gynecologic Cancer Intergroup has developed CA125 progression criteria which are to be integrated with objective tumor assessment for use in first-line trials in ovarian cancer.[^19^](#_bookmark16)

*Cytology, histology:* These techniques can be used to differentiate between PR and CR in rare cases if required by protocol (for example, residual lesions in tumor types such as germ cell tumors, where known residual benign tumors can re- main). When effusions are known to be a potential adverse effect of treatment (e.g., with certain taxane compounds or angiogenesis inhibitors), the cytological confirmation of the neoplastic origin of any effusion that appears or worsens during treatment can be considered if the measurable tumor has met criteria for response or stable disease in order to differentiate between response (or stable disease) and progressive disease.

2. Tumour response evaluation

**2．1 Assessment of overall tumor burden and measurable disease**

To assess objective response or future progression, it is necessary to estimate the *overall tumor burden at baseline* and use this as a comparator for subsequent measurements. Only patients with measurable disease at baseline should be included in protocols where objective tumor response is the primary end-point. Measurable disease is defined by the presence of at least one measurable lesion (as detailed above in Section 3). In studies where the primary end-point is tumor progression (either time to progression or proportion with progression at a fixed date), the protocol must specify if entry is restricted to those with measurable disease or whether patients having non-measurable disease only are also eligible.

- 1. **Baseline documentation of ‘target’ and ‘non-target’ lesions**

When more than one measurable lesion is present at baseline, all lesions up to a maximum of five lesions total (and a maximum of two lesions per organ) representative of all involved organs should be identified as *target lesions* and will be recorded and measured at baseline (this means in instances where patients have only one or two organ sites involved a *maximum* of two and four lesions respectively will be re- corded). For evidence to support the selection of only five target lesions, see analyses on a large prospective database in the article by Bogaerts et al.[^10^](#_bookmark12).

Target lesions should be selected on the basis of their size (lesions with the longest diameter), be representative of all involved organs, but addition should be those that lend themselves to *reproducible repeated measurements*. It may be the case that, on occasion, the largest lesion does not lend itself to reproducible measurement in which circumstance the next largest lesion which can be measured reproducibly should be selected. To illustrate this point, see the example in Fig. 3 of Appendix II.

*Lymph nodes* merit special mention since they are normal anatomical structures that may be visible by imaging even if not involved by tumor. As noted in Section 3, pathological nodes which are defined as measurable and may be identified as target lesions must meet the criterion of a short axis of P15 mm by CT scan. Only the *short* axis of these nodes will contribute to the baseline sum. The short axis of the node is the diameter normally used by radiologists to judge if a node is involved in a solid tumor. Nodal size is normally reported as two dimensions in the plane in which the image is obtained (for CT scan, this is almost always the axial plane; for MRI, the plane of acquisition may be axial, sagittal, or coronal). The smaller of these measures is the short axis. For example, an abdominal node which is reported as being 20 mm · 30 mm has a short axis of 20 mm and qualifies as a malignant, measurable node. In this example, 20 mm should be recorded as the node measurement (See also the example in Fig. 4 in Appendix II). All other pathological nodes (those with short axis P10 mm but <15 mm) should be considered non-target lesions. Nodes that have a short axis <10 mm are considered non-pathological and should not be recorded or followed.

A *sum of the diameters* (longest for non-nodal lesions, short

axis for nodal lesions) for all target lesions will be calculated and reported as the *baseline sum diameters*. If lymph nodes are to be included in the sum, then as noted above, only the *short* axis is added to the sum. The baseline sum diameters will be used as a reference to further characterize any objective tumor regression in the measurable dimension of the disease.

All other lesions (or sites of disease), including pathological lymph nodes, should be identified as *non-target lesions* and should also be recorded at baseline. Measurements are not required and these lesions should be followed as ‘present’, ‘absent’, or in rare cases, ‘unequivocal progression’ (more details to follow). In addition, it is possible to record multiple non-target lesions involving the same organ as a single item on the case record form (e.g., ‘multiple enlarged pelvic lymph nodes’ or ‘multiple liver metastases’).

- 1. **Response criteria**

This section provides the definitions of the criteria used to determine objective tumor response for target lesions.

- - 1. **Evaluation of target lesions**

Complete Response (CR): Disappearance of all target lesions. Any pathological lymph nodes (whether target or non-target) must have a reduction in short axis to

<10 mm.

Partial Response (PR): At least a 30% decrease in the sum of diameters of target lesions, taking as reference the baseline sum diameters.

Progressive Disease (PD): At least a 20% increase in the sum of diameters of target lesions, taking as reference the *smallest sum obtained in the study* (this includes the baseline sum if that is the smallest in the study). In addition to the relative increase of 20%, the sum must also demonstrate an absolute increase of at least 5 mm. (*Note:* the appearance of one or more new lesions is also considered progression).

Stable Disease (SD): Neither sufficient shrinkage to qualify for PR nor sufficient increase to qualify for PD, taking as reference the smallest sum diameters while in this study.

- - 1. *Special notes on the assessment of target lesions Lymph nodes.*

Lymph nodes identified as target lesions should always have the actual short axis measurement recorded (measured in the same anatomical plane as the baseline examination), even if the nodes regress to below 10 mm during the study. This means that when lymph nodes are included as target lesions, the ‘sum’ of lesions may not be zero even if complete response criteria are met since a normal lymph node is defined as having a short axis of <10 mm. Case report forms or other data collection methods may therefore be designed to have target nodal lesions recorded in a separate section where, in order to qualify for CR, each node must achieve a short axis <10 mm. For PR, SD, and PD, the actual short-axis measurement of the nodes are to be included in the sum of target lesions.

*Target lesions that become ‘too small to measure’.* During the study, all lesions (nodal and non-nodal) recorded at baseline should have their actual measurements recorded at each subsequent evaluation, even when very small (e.g., 2 mm). However, sometimes lesions or lymph nodes which are recorded as target lesions at baseline become so faint on a CT scan that the radiologist may not feel comfortable assigning an exact measure and may report them as being ‘too small to measure’. When this occurs, it is important that a value be recorded on the case report form. If it is the opinion of the radiologist that the lesion has likely disappeared, the measurement should be recorded as 0 mm. If the lesion is believed to be present and is faintly seen but too small to measure, a default value of 5 mm should be assigned (*Note:* It is less likely that this rule will be used for lymph nodes since they usually have a definable size when normal and are frequently surrounded by fat such as in the retroperitoneum; however, if a lymph node is believed to be present and is faintly seen but too small to measure, a de- fault value of 5 mm should be assigned in this circumstance as well). This default value is derived from the 5 mm CT slice thickness (but should not be changed with varying CT slice thickness). The measurement of these lesions is potentially non-reproducible, therefore, providing this default value will prevent false responses or progressions based upon measurement error. To reiterate, however, if the radiologist *is* able to provide an actual measure, that should be recorded, even if it is below 5 mm.

*Lesions that split or coalesce on treatment.* As noted in Appendix II, when non-nodal lesions ‘fragment’, the longest diameters of the fragmented portions should be added together to calculate the target lesion sum. Similarly, as lesions coalesce, a plane between them may be maintained that would aid in obtaining maximal diameter measurements of each individual lesion. If the lesions have truly coalesced such that they are no longer separable, the vector of the longest diameter in this instance should be the maximal longest diameter for the ‘coalesced lesion’.

- - 1. **Evaluation of non-target lesions**

This section provides the definitions of the criteria used to determine the tumor response for the group of non-target lesions. While some non-target lesions may be measurable, they need not be measured and instead should be assessed only *qualitatively* at the time points specified in the protocol.

Complete Response (CR): Disappearance of all non-target lesions and normalization of tumor marker level. All lymph nodes must be non-pathological in size (<10 mm short axis).

Non-CR/Non-PD: Persistence of one or more non-target lesion(s) and/or maintenance of tumor marker level above the normal limits.

Progressive Disease (PD): *Unequivocal progression* (see comments below) of existing non-target lesions. (*Note:* the appearance of one or more new lesions is also considered progression).

- - 1. **Special notes on assessment of progression of non-target disease**

The concept of progression of non-target disease requires additional explanation as follows:

*When the patient also has measurable disease.* In this setting, to achieve ‘unequivocal progression’ on the basis of the non-target disease, there must be an overall level of substantial worsening in non-target disease such that, even in the presence of SD or PR in the target disease, the overall tumor burden has increased sufficiently to merit discontinuation of therapy (see examples in Appendix II and further details below). A modest ‘increase’ in the size of one or more non-target lesions is usually not sufficient to qualify for unequivocal progression status. The designation of overall progression *solely* on the basis of change in non-target disease in the face of SD or PR of target disease will therefore be extremely rare.

*When the patient has only non-measurable disease.* This circum- stance arises in some phase III trials when it is not a criterion of study entry to have measurable disease. The same general concepts apply here as noted above, however, in this instance, there is no measurable disease assessment to factor into the interpretation of an increase in non-measurable disease burden. Because worsening in non-target disease cannot be easily quantified (by definition: if all lesions are truly non-measurable), a useful test that can be applied when assessing patients for unequivocal progression is to consider if the increase in overall disease burden based on the change in non-measurable disease is comparable in magnitude to the increase that would be required to declare PD for measurable disease: i.e., an increase in tumor burden representing an additional 73% increase in ‘volume’ (which is equivalent to a 20% increase diameter in a measurable lesion). Examples include an increase in a pleural effusion from ‘trace’ to ‘large’, an increase in lymphangitic disease from localized to widespread, or may be described in protocols as ‘sufficient to require a change in therapy’. Some illustrative examples are shown in Figs. 5 and 6 in Appendix II. If ‘unequivocal progression’ is seen, the patient should be considered to have had overall PD at that point. While it would be ideal to have objective criteria to apply to non-measurable diseases, the very nature of that disease makes it impossible to do so, therefore the increase must be substantial.

- - 1. **New lesions**

The appearance of new malignant lesions denotes disease progression; therefore, some comments on the detection of new lesions are important. There are no specific criteria for the identification of new radiographic lesions; however, the finding of a new lesion should be unequivocal: i.e., not attributable to differences in scanning technique, change in imaging modality, or findings thought to represent something other than tumor (for example, some ‘new’ bone lesions may be simply healing or flare of pre-existing lesions). This is particularly important when the patient’s baseline lesions show partial or complete response. For example, necrosis of a liver lesion may be reported on a CT scan report as a ‘new’ cystic lesion, which it is not.

A lesion identified on a follow-up study in an anatomical location that was *not* scanned at baseline is considered a new lesion and will indicate disease progression. An example of this is the patient who has the visceral disease at baseline and, while in the study, has a CT or MRI brain ordered, which reveals metastases. The patient’s brain metastases are considered to be evidence of PD even if he/she did not have brain imaging at baseline.

If a new lesion is equivocal, for example, because of its small size, continued therapy and follow-up evaluation will clarify if it represents a truly new disease. If repeat scans confirm there is definitely a new lesion, then progression should be declared using the date of the initial scan.

While FDG-PET response assessments need additional study, it is sometimes reasonable to incorporate the use of FDG-PET scanning to complement CT scanning in the assessment of progression (particularly possible ‘new’ disease). New lesions on the basis of FDG-PET imaging can be identified according to the following algorithm:

a. Negative FDG-PET at baseline, with a positive FDG-PET at follow-up, is a sign of PD based on a new lesion.

b. No FDG-PET at baseline and a positive FDG-PET at follow-up

If the positive FDG-PET at follow-up corresponds to a new site of disease confirmed by CT, this is PD.

If the positive FDG-PET at follow-up is not confirmed as a new site of disease on CT, additional follow-up CT scans are needed to determine if there is true progression occurring at that site (if so, the date of PD will be the date of the initial abnormal FDG-PET scan).

If the positive FDG-PET at follow-up corresponds to a pre-existing site of disease on CT that is not progressing on the basis of the anatomic images, this is not PD.

- 1. **Evaluation of best overall response**

The best overall response is the best response recorded from the start of the study treatment until the end of treatment, considering any requirement for confirmation. On occasion, a response may not be documented until after the end of therapy so protocols should be clear if post-treatment assessments are to be considered in the determination of best overall response. Protocols must specify how any new therapy introduced before progression will affect the best response designation. The patient’s best overall response assignment will depend on the findings of both target and non-target disease and will also take into consideration the appearance of new lesions. Furthermore, depending on the nature of the study and the protocol requirements, it may also require confirmatory measurement (see Section 4.6). Specifically, in non-randomized trials where the response is the primary end-point, confirmation of PR or CR is needed to deem either one the ‘best overall response’. This is described further below.

- - 1. **Timepoint response**

It is assumed that at each protocol-specified time point, a response assessment occurs. Table 1 on the next page provides a summary of the overall response status calculation at each time point for patients who have measurable disease at baseline.

When patients have non-measurable (therefore non-tar- get) disease only, Table 2 is to be used.

- - 1. **Missing assessments and unevaluable designation**

When no imaging/measurement is done at all at a particular time point, the patient is not evaluable (NE) at that time point. If only a subset of lesion measurements are made at an assessment, usually the case is also considered NE at that time point, unless a convincing argument can be made that the contribution of the individual missing lesion(s) would not change the assigned time point response. This would be most likely to happen in the case of PD. For example, if a patient had a baseline sum of 50 mm with three measured lesions and at follow-up only two lesions were assessed, but those gave a sum of 80 mm, the patient will have achieved PD status, regardless of the contribution of the missing lesion.

- - 1. **Best overall response: all-time points**

The *best overall response* is determined once all the data for the patient is known.

*Best response determination in trials where confirmation of complete or partial response IS NOT required*: Best response in these trials is defined as the best response across all time points (for example, a patient who has SD at the first assessment, PR at the second assessment, and PD on last assessment has a best overall response of PR). When SD is believed to be the best response, it must also meet the protocol-specified minimum time from baseline. If the minimum time is not met when SD is otherwise the best time point response, the patient’s best response depends on the subsequent assessments. For example, a patient who has SD at the first assessment, PD at the second, and does not meet the minimum duration for SD, will have the best response of PD. The same patient lost to follow-up after the first SD assessment would be considered unevaluable.

*Best response determination in trials where confirmation of complete or partial response IS required*: Complete or partial responses may be claimed only if the criteria for each are met at a subsequent time point as specified in the protocol (generally 4 weeks later). In this circumstance, the best overall response can be interpreted as in Table 3.

**2.4.4 Special notes on response assessment**

When nodal disease is included in the sum of target lesions and the nodes decrease to ‘normal’ size (<10 mm), they may still have a measurement reported on scans. This measurement should be recorded even though the nodes are normal in order not to overstate progression should it be based on the increase in the size of the nodes. As noted earlier, this means that patients with CR may not have a total sum of ‘zero’ on the case report form (CRF).

In trials where confirmation of response is required, re- peated ‘NE’ time point assessments may complicate best response determination. The analysis plan for the trial must address how missing data/assessments will be addressed in the determination of response and progression. For example, in most trials, it is reasonable to consider a patient with time point responses of PR-NE-PR as a confirmed response.

Patients with a global deterioration of health status requiring discontinuation of treatment without objective evidence of disease progression at that time should be reported as ‘symptomatic deterioration’. Every effort should be made to document objective progression even after discontinuation of treatment. Symptomatic deterioration is *not* a descriptor of an objective response: it is a reason for stopping study therapy. The objective response status of such patients is to be determined by evaluation of target and non-target disease as shown in Tables 1–3.

Conditions that define ‘early progression, early death and evaluability are study specific and should be clearly described in each protocol (depending on treatment duration, and treatment periodicity).

In some circumstances it may be difficult to distinguish residual disease from normal tissue. When the evaluation of complete response depends upon this determination, it is recommended that the residual lesion be investigated (fine needle aspirate/biopsy) before assigning a status of complete response. FDG-PET may be used to upgrade a response to a CR in a manner similar to a biopsy in cases where a residual radiographic abnormality is thought to represent fibrosis or scarring. The use of FDG-PET in this circumstance should be prospectively described in the protocol and supported by dis- ease specific medical literature for the indication. However, it must be acknowledged that both approaches may lead to false positive CR due to limitations of FDG-PET and biopsy resolution/sensitivity.

For equivocal findings of progression (e.g. very small and uncertain new lesions; cystic changes or necrosis in existing lesions), treatment may continue until the next scheduled assessment. If at the next scheduled assessment, progression is confirmed, the date of progression should be the earlier date when progression was suspected.

**2.5. Frequency of tumor re-evaluation**

Frequency of tumor re-evaluation while on treatment should be protocol specific and adapted to the type and schedule of treatment. However, in the context of phase II studies where the beneficial effect of therapy is not known, follow-up every 6–8 weeks (timed to coincide with the end of a cycle) is reasonable. Smaller or greater time intervals than these could be justified in specific regimens or circumstances. The protocol should specify which organ sites are to be evaluated at baseline (usually those most likely to be involved with metastatic disease for the tumor type under study) and how often evaluations are repeated. Normally, all target and non-target sites are evaluated at each assessment. In selected circum- stances certain non-target organs may be evaluated less frequently. For example, bone scans may need to be repeated only when complete response is identified in target disease or when progression in bone is suspected.

After the end of the treatment, the need for repetitive tumor evaluations depends on whether the trial has as a goal the response rate or the time to an event (progression/death). If ‘time to an event’ (e.g., time to progression, disease-free survival, progression-free survival) is the main end-point of the study, then routinely scheduled re-evaluation of protocol-specified sites of disease is warranted. In randomized comparative trials in particular, the scheduled assessments should be performed as identified on a calendar schedule (for example: every 6–8 weeks on treatment or every 3–4 months after treatment) and should not be affected by delays in therapy, drug holidays or any other events that might lead to imbalance in a treatment arm in the timing of disease assessment.

**2.6. Confirmatory measurement/duration of response**

**2.6.1**. Confirmation

In non-randomized trials where response is the primary end-point, confirmation of PR and CR is required to ensure responses identified are not the result of measurement error. This will also permit appropriate interpretation of results in the context of historical data where response has traditionally required confirmation in such trials (see the paper by Bogaerts et al. in this Special Issue[^10^](#_bookmark12)). However, in all other circum- stances, i.e., in randomized trials (phase II or III) or studies where stable disease or progression are the primary end-points, confirmation of response is not required since it will not add value to the interpretation of trial results. However, elimination of the requirement for response confirmation may increase the importance of a central review to protect against bias, in particular in studies that are not blinded.

In the case of SD, measurements must have met the SD criteria at least once after study entry at a minimum interval (in general not less than 6–8 weeks) that is defined in the study protocol.

- - 1. **Duration of overall response**

The duration of overall response is measured from the time measurement criteria are first met for CR/PR (whichever is first recorded) until the first date that recurrent or progressive dis- ease is objectively documented (taking as reference for progressive disease the smallest measurements recorded in the study).

The duration of the overall complete response is measured from the time measurement criteria are first met for CR until the first date that recurrent disease is objectively documented.

- - 1. **Duration of stable disease**

Stable disease is measured from the start of the treatment (in randomized trials, from the date of randomization) until the criteria for progression are met, taking as reference the *smallest sum in the study* (if the baseline sum is the smallest, this is the reference for calculation of PD).

The clinical relevance of the duration of stable disease varies in different studies and diseases. If the proportion of patients achieving stable disease for a minimum period of time is an end-point of importance in a particular trial, the protocol should specify the minimum time interval required between two measurements for determination of stable disease.

*Note*: The duration of response and stable disease as well as the progression-free survival are influenced by the frequency of follow-up after baseline evaluation. It is not in the scope of this guideline to define a standard follow-up frequency. The frequency should take into account many parameters including disease types and stages, treatment periodicity and standard practice. However, these limitations of the precision of the measured end-point should be taken into account if comparisons between trials are to be made.

- 1. **Progression-free survival/proportion progression-free**
     1. *Phase II trials*

This guideline is focused primarily on the use of objective response end-points for phase II trials. In some circumstances, ‘response rate’ may not be the optimal method to assess the potential anticancer activity of new agents/regimens. In such cases ‘progression-free survival’ (PFS) or the ‘proportion progression-free’ at landmark time points, might be considered appropriate alternatives to provide an initial signal of biological effect of new agents. It is clear, however, that in an uncontrolled trial, these measures are subject to criticism since an apparently promising observation may be related to biological factors such as patient selection and not the impact of the intervention. Thus, phase II screening trials utilizing these end-points are best designed with a randomized control. Exceptions may exist where the behavior patterns of certain cancers are so consistent (and usually consistently poor), that a non-randomized trial is justifiable (see for example van Glabbeke et al.[^20^](#_bookmark17)). However, in these cases it will be essential to document with care the basis for estimating the expected PFS or proportion progression-free in the absence of a treatment effect.

**2.7.2 Phase III trials**

Phase III trials in advanced cancers are increasingly designed to evaluate progression-free survival or time to progression as the primary outcome of interest. Assessment of progression is relatively straightforward if the protocol requires all patients to have measurable disease. However, restricting entry to this subset of patients is subject to criticism: it may result in a trial where the results are less likely to be generalizable if, in the disease under study, a substantial proportion of patients would be excluded. Moreover, the restriction to entry will slow recruitment to the study. Increasingly, therefore, trials allow entry of both patients with measurable disease as well as those with non-measurable disease only. In this circumstance, care must be taken to explicitly describe the findings which would qualify for progressive disease for those patients *without* measurable lesions. Furthermore, in this set- ting, protocols must indicate if the maximum number of re- corded target lesions for those patients with measurable disease may be relaxed from five to three (based on the data found in Bogaerts et al.[^10^](#_bookmark12) and Moskowitz et al.[^11^](#_bookmark13)). As found in the ‘special notes on assessment of progression’, these guidelines offer recommendations for assessment of progression in this setting. Furthermore, if available, validated tumor marker measures of progression (as has been proposed for ovarian cancer) may be useful to integrate into the definition of progression. Centralized blinded review of imaging studies or of source imaging reports to verify ‘unequivocal progression’ may be needed if important drug development or drug approval decisions are to be based on the study outcome. Finally, as noted earlier, because the date of progression is subject to ascertainment bias, the timing of investigations in study arms should be the same. The article by Dancey et al. in this special issue[^21^](#_bookmark17) provides a more detailed discussion of the assessment of progression in randomized trials.

- 1. **Independent review of response and progression**

For trials where *objective response* (CR + PR) is the primary end-point, and in particular where key drug development decisions are based on the observation of a minimum number of responders, it is recommended that all claimed responses be reviewed by an expert(s) independent of the study. If the study is a randomized trial, ideally reviewers should be blinded to treatment assignment. Simultaneous review of the patients’ files and radiological images is the best approach.

Independent review of progression presents some more complex issues: for example, there are statistical problems with the use of central-review-based progression time in place of investigator-based progression time due to the potential introduction of informative censoring when the former precedes the latter. An overview of these factors and other lessons learned from independent review is provided in an article by Ford et al. in this special issue.

- 1. **Reporting best response results**
     1. *Phase II trials*

When response is the primary end-point, and thus all patients must have a measurable disease to enter the trial, all patients included in the study must be accounted for in the report of the results, even if there are major protocol treatment deviations or if they are not evaluable. Each patient will be assigned one of the following categories:

1. Complete response
2. Partial response
3. Stable disease
4. Progression
5. Unevaluable for response: specify reasons (for example: early death, malignant disease; early death, toxicity; tumor assessments not repeated/incomplete; other (specify)).

Normally, all *eligible* patients should be included in the denominator for the calculation of the response rate for phase II trials (in some protocols it will be appropriate to include all treated patients). It is generally preferred that 95% two-sided confidence limits are given for the calculated response rate. Trial conclusions should be based on the response rate for all eligible (or all treated) patients and should *not* be based on a selected ‘evaluable’ subset.

- - 1. **Phase III trials**

Response evaluation in phase III trials may be an indicator of the relative anti-tumor activity of the treatments evaluated and is almost always a secondary end-point. Observed differences in response rate may not predict the clinically relevant therapeutic benefit for the population studied. If objective response is selected as a primary end-point for a phase III study (only in circumstances where a direct relationship between objective tumor response and a clinically relevant therapeutic benefit can be unambiguously demonstrated for the population studied), the same criteria as those applying to phase II trials should be used and all patients entered should have at least one measurable lesion.

In those many cases where response is a secondary end-

point, and not all trial patients have measurable disease, the method for reporting overall best response rates must be pre-specified in the protocol. In practice, response rate may be reported using either an ‘intent to treat’ analysis (all randomized patients in the denominator) or an analysis where only the subset of patients with measurable disease at baseline are included. The protocol should clearly specify how response results will be reported, including any subset analyses that are planned.

The original version of RECIST suggested that in phase III trials, one could write protocols using a ‘relaxed’ interpretation of the RECIST guidelines (for example, reducing the number of lesions measured), but this should no longer be done since these revised guidelines have been amended in such a way that it is clear how these criteria should be applied for all trials in which anatomical assessment of tumor response or progression are end-points.


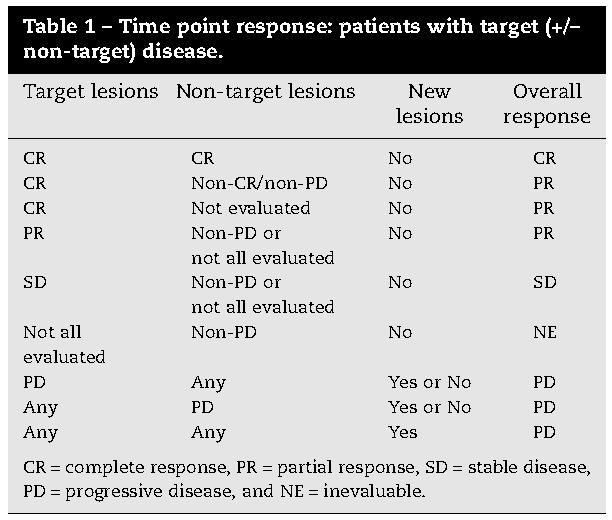


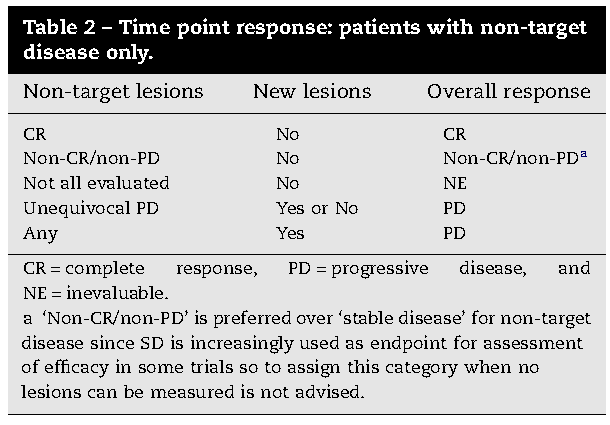


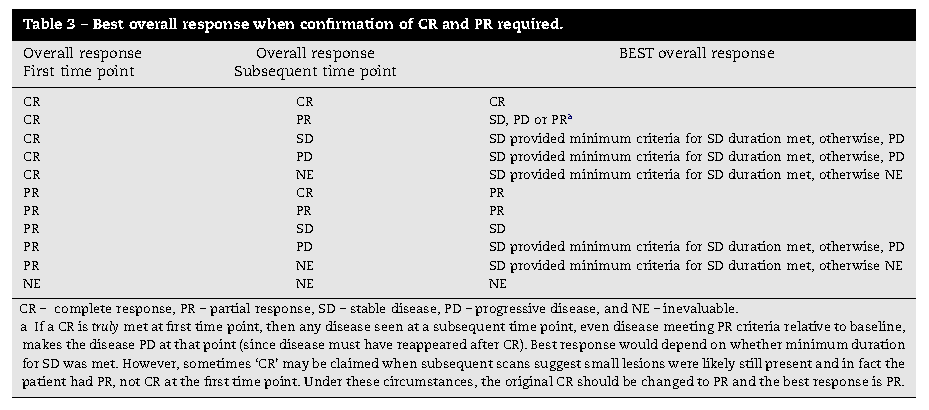


**APPENDIX V Classification of Surgical Complications：Clavien–Dindo Grade**

The severity of complications was graded according to the Clavien-Dindo grading system (Clavien-Dindo grading system), and grade IIIA and above were considered as serious complications.

Ⅰ：Any deviation from the normal postoperative course without the need for pharmacological treatment or surgical, endoscopic, and radiological interventions. Allowed therapeutic regimens are: drugs as antiemetics, antipyretics, analgesics, diuretics, electrolytes, and physiotherapy. This grade also includes wound infections opened at the bedside

Ⅱ：Requiring pharmacological treatment with drugs other than such allowed for grade I complications Blood transfusions and total parenteral nutrition are also included.

Ⅲ：Requiring surgical, endoscopic or radiological intervention. Ⅲa：Intervention not under general anesthesia；Ⅲb：Intervention under general anesthesia.

Ⅳ：Life-threatening complication (including CNS complications)* requiring IC/ICU management. Ⅳa：Single organ dysfunction (including dialysis)；Ⅳb：Multiorgan dysfunction.

Ⅴ：Death of a patient.

*Brain hemorrhage, ischemic stroke, subarachnoid bleeding, but excluding transient ischemic attacks. CNS, central nervous system; IC, intermediate care; ICU, intensive care unit.
